# m^6^A‐mRNA Reader *YTHDF2* Identified as a Potential Risk Gene in Autism With Disproportionate Megalencephaly

**DOI:** 10.1002/aur.3314

**Published:** 2025-01-30

**Authors:** Sierra S. Nishizaki, Nicholas K. Haghani, Gabriana N. La, Natasha Ann F. Mariano, José M. Uribe‐Salazar, Gulhan Kaya, Melissa Regester, Derek Sayre Andrews, Christine Wu Nordahl, David G. Amaral, Megan Y. Dennis

**Affiliations:** ^1^ Genome Center University of California Davis CA USA; ^2^ Autism Research Training Program University of California Davis CA USA; ^3^ Department of Psychiatry and Behavioral Sciences University of California Davis CA USA; ^4^ MIND Institute University of California Davis CA USA; ^5^ Department of Biochemistry & Molecular Medicine University of California Davis CA USA; ^6^ Postbaccalaureate Research Education Program University of California Davis California USA

**Keywords:** autism, disproportionate megalencephaly, genetics, m^6^A‐RNA modification, YTHDF2, zebrafish

## Abstract

Among autistic individuals, a subphenotype of disproportionate megalencephaly (ASD‐DM) seen at three years of age is associated with co‐occurring intellectual disability and poorer prognoses later in life. However, many of the genes contributing to ASD‐DM have yet to be delineated. In this study, we identified additional ASD‐DM candidate genes with the aim to better define the genetic etiology of this subphenotype of autism. We expanded the previously studied sample size of ASD‐DM individuals ten fold by including probands from the Autism Phenome Project and Simons Simplex Collection, totaling 766 autistic individuals meeting the criteria for megalencephaly or macrocephaly and revealing 154 candidate ASD‐DM genes harboring *de novo* protein‐impacting variants. Our findings include 14 high confidence autism genes and seven genes previously associated with DM. Five impacted genes have previously been associated with both autism and DM, including *CHD8* and *PTEN*. By performing functional network analysis, we expanded to additional candidate genes, including one previously implicated in ASD‐DM (*PIK3CA*) as well as 184 additional genes connected with ASD or DM alone. Using zebrafish, we modeled a *de novo* tandem duplication impacting *YTHDF2*, encoding an N6‐methyladenosine (m^6^A)‐mRNA reader, in an ASD‐DM proband. Testing zebrafish CRISPR knockdown led to reduced head/brain size, while overexpressing *YTHDF2* resulted in increased head/brain size matching that of the proband. Single‐cell transcriptomes of *YTHDF2* gain‐of‐function larvae point to reduced expression of Fragile‐X‐syndrome‐associated FMRP‐target genes globally and in the developing brain, providing insight into the mechanism underlying autistic phenotypes. We additionally discovered a variant impacting a different gene encoding an m^6^A reader, *YTHDC1*, in our ASD‐DM cohort. Though we highlight only two cases to date, our study provides support for the m^6^A‐RNA modification pathway as potentially contributing to this severe form of autism.

**Summary**

- Autism (ASD) has become increasingly prevalent in children in recent years.
- While we know ASD is associated with hundreds of genes, there is much to learn about what genes are involved and how they contribute to its diverse presentation of traits and behaviors.
- By focusing on autistic individuals exhibiting enlarged brains (disproportionate megalencephaly), we identified new candidate genes possibly contributing to ASD and brain size.
- An autistic‐patient‐identified duplication of one gene in particular, *YTHDF2*, implicates a novel pathway related to RNA modifications with ASD and human brain size for the first time.

## 1 Introduction

Autism is a group of neurodevelopmental traits characterized by difficulties with communication, social interactions, and behavioral challenges, prevalent in 1 out of 36 children in the United States (Maenner 2023). Autism is highly heritable, with 50%–90% of cases estimated to be driven by genetics alone (Sandin et al. 2014, 2016; Castelbaum et al. 2020). Autism is also highly heterogeneous with large‐scale whole exome sequencing (WES) of > 63,000 autistic probands identifying 125 high confidence autism genes, with the predicted number of genes left to be discovered exceeding 1000 (Satterstrom et al. 2020; Fu et al. 2022; Leblond et al. 2021). In particular, leveraging genomic data from autism families—including parents and unaffected siblings—in the Simons Simplex Collection (SSC) (Fischbach and Lord 2010) has identified coding *de novo* variants estimated to contribute to 30% of diagnoses (Iossifov et al. 2014; Sanders et al. 2012; Belyeu et al. 2021). More recently, whole genome sequencing (WGS) of SSC *de novo* noncoding mutations implicates risk in an additional 4.3% of autism cases (An et al. 2018; Zhou et al. 2019). Despite the combined efforts to sequence tens of thousands of genomes, known genes still only account for 5%–20% of cases, and further work is required to fully elucidate genes and pathways contributing to autism etiology (Satterstrom et al. 2020; Fu et al. 2022; Antaki et al. 2022; Warrier et al. 2022; Zhou et al. 2022; Trost et al. 2022; Rylaarsdam and Guemez‐Gamboa 2019; Wang et al. 2022; Rolland et al. 2023; Wilfert et al. 2021; Cirnigliaro et al. 2023). Combining *de novo* variation with autism sub‐phenotyping has been used to address the heterogeneity of autism and identify susceptibility loci for comorbid phenotypes in an acute way (Liu, Paterson, and Szatmari 2008).

Brain enlargement that is disproportionate to height, known as disproportionate megalencephaly (DM), is enriched in autistic probands with 15% of autistic boys falling under the DM subphenotype (ASD‐DM) compared to 6% in typically developing boys (Amaral et al. 2017). This comorbidity is associated with more severe cognitive phenotypes, including lower IQ and language use, as well as higher rates of language regression (Chawarska 2011; Nordahl et al. 2011; Sacco et al. 2007). This robust enrichment and distinct presentation support DM as a sub‐phenotype of ASD, likely due to a shared genetic etiology between autism and DM. While a handful of genes have been associated with DM—including known autism genes impacting cell cycle and proliferation during embryonic development (e.g., *CHD8* and *PTEN*)—mutations of known candidate genes make up only 3% of megalencephaly in autism probands. This leaves the genetic etiology of a majority of ASD‐DM cases undiscovered (Hormozdiari et al. 2015; Krishnan et al. 2016; O'Roak et al. 2012; Willsey et al. 2013). A study using WES from 46 autistic families with macrocephaly (ASD‐M)—defined as > 2 standard deviations above the mean head circumference for typically developing sex‐ and age‐matched children—successfully identified mutations in one novel and several known autism candidate genes (Wu et al. 2020), demonstrating the power of sub‐phenotyping ASD‐DM leading to genetic discoveries even for reduced sample size.

Zebrafish ( *Danio rerio* ) are an attractive model for studying neurodevelopmental traits given their rapid development, large number of progeny, transparent bodies, and that ~70% of gene orthologs are shared with humans (Abreu et al. 2020; Howe et al. 2013; Weinschutz Mendes et al. 2023). Previous studies of known ASD‐DM genes recapitulate macrocephaly and DM phenotypes in zebrafish knockdown and knockout experiments for *CHD8* and *KMT2E*, respectively (Bernier et al. 2014; Thyme et al. 2019). This method of knocking down candidate ASD‐M genes has also been used systematically to identify the contributing gene in the chromosome 16p11.2 locus in zebrafish (Golzio et al. 2012). Further, novel technologies such as the VAST BioImaging System allow for the rapid characterization of zebrafish knockout models through the generation of high resolution standardized images (Pulak 2016). We demonstrated the utility of this approach by examining the knockdown of two genes associated with autism and microcephaly, *SLC7A5* and *SYNGAP1*, by assessing head‐size phenotypes in CRISPR‐generated zebrafish knockout line embryos at three and 5 days post fertilization (dpf) (Colón‐Rodríguez et al. 2020).

In this study, we leveraged high‐coverage WGS data from two cohorts, 11 ASD‐DM probands from the UC Davis MIND Institute Autism Phenome Project (APP) specifically identified using magnetic resonance imaging (MRI) data at around 3 years of age, and 755 ASD‐M probands with head circumference data available from the SSC cohort. Together, this represents a > 10‐fold increase in probands compared with the previous largest study of increased head circumference associated genes in ASD (Wu et al. 2020). Using this sub‐phenotype‐to‐genotype analytic strategy, we identified candidate ASD‐DM and ASD‐M genes harboring *de novo* likely gene‐disruptive variants, including high‐confidence *CHD8* and *PTEN* as well as novel candidates. We subsequently modeled a *de novo YTHDF2* partial tandem duplication identified in an ASD‐DM proband using zebrafish resulting in macrocephaly in zebrafish following embryonic microinjection of mRNA encoding the gene. Together our sub‐phenotyping approach provides a powerful strategy to identify novel ASD‐DM candidate genes and validate their role in brain development using a zebrafish model system.

## 2 Methods

### 2.1 Megalencephaly and Macrocephaly Phenotypes

APP probands and determinations of megalencephaly were previously determined as part of the APP study (Amaral et al. 2017). Acquisition of MRI data for megalencephaly measurements were made during natural nocturnal sleep for children at study enrollment (time point 1), between the ages of 2 and 3.5 (Lee et al. 2021). This research was prospectively reviewed and approved by the UC Davis Institutional Review Board. Blood collected during this time point was sequenced using 30× coverage WGS through a collaboration with MSSNG (Chan et al. 2022; Yuen et al. 2017). Raw data including FASTQ and VCF files can be accessed through the MSSNG access agreement: https://research.mss.ng.

Macrocephaly cases from the SSC were defined using a permissive cutoff of a head circumference > 1.5 standard deviations (90%) above the mean of age‐matched controls (Klein, Sharifi‐Hannauer, and Martinez‐Agosto 2013). Age‐matched typically developing head circumference data (Rollins, Collins, and Holden 2010) and height data (Centers for Disease Control and Prevention, National Center for Health Statistics n.d.) from ages 4–17 were derived from publicly available standards. For males in the SSC cohort 551/1601 (34%) met the criteria for macrocephaly, for females 108/245 (45%) met this criteria. We identified three types of macrocephaly: (1) somatic overgrowth (SO) with head circumference and height percentiles > 90%; (2) disproportionate macrocephaly (DMac) with height percentiles over head circumference percentile < 0.7; and (3) relative macrocephaly (RM) with height percentiles over head circumference percentile > 0.7.

### 2.2 Variant Annotation

Whole‐genome sequencing, read mapping, and variant identification were performed for APP families as part of the MSSNG consortium (Trost et al. 2022; Chan et al. 2022; Yuen et al. 2017). *De novo* variants were identified in APP probands as those unique to the proband and absent from either parent via string matching (grep ‐Fvxf). We considered likely gene‐disruptive variants as those predicted to lead to a frameshift, nonsense, or splice site mutation. Rare variants were identified using dbSNP as those with a minor allele frequency (MAF) < 0.2% in all five 1000 Genomes Project ancestry‐based populations (Sherry et al. 2001; 1000 Genomes Project Consortium et al. 2015). The presence of all rare and *de novo* variants identified in the APP cohort were validated by visual inspection of sequencing data via the Integrated Genomics Viewer (IGV) (Thorvaldsdóttir, Robinson, and Mesirov 2013). For SSC, *de novo* single‐nucleotide variants (SNVs) and indels were previously identified (An et al. 2018). SSC *de novo* copy‐number variants (CNVs) identified as precise and exonic (impacting no more than two genes) from a recent MSSNG study (Trost et al. 2022) were also included in this analysis.

### 2.3 Network and Ontology Analyses

Network analysis of known ASD‐DM genes and candidate ASD‐DM genes from this study were completed using the STRING database and visualized via Cytoscape (Szklarczyk et al. 2015; Shannon et al. 2003). Gene ontology (GO) analysis was completed for known ASD‐DM genes and candidate ASD‐DM genes as seed genes along with their top 10 gene interactors determined using the STRING database, similar to previous studies (Willsey et al. 2013). Similar STRING database molecular function GO terminology was pooled. GO was completed using database for annotations, visualization, and integrated discovery (DAVID) software (Sherman et al. 2022). Human genes were used as background for GO analyses.

### 2.4 Zebrafish CRISPR and mRNA Models

The protocols and procedures employed related to zebrafish were ethically reviewed and approved by the UC Davis Institutional Animal Care and Use Committee (accredited by the Association for Assessment and Accreditation of Laboratory Animal Care with Animal Welfare Assurance Number D16‐00272 (A3433‐01)). Guide RNAs (gRNAs) were selected as having a CRISPRScan score of 35 or higher (Table S1) (Moreno‐Mateos et al. 2015). crRNAs were synthesized by Integrated DNA Technologies. Injection mixes of ribonucleic protein (RNP) consisting of four pooled gRNAs (annealed crRNA and tracrRNA) and SpCas9 Nuclease (New England Biolabs, M0386M) in order to achieve 90% knockdown efficiency (Wu et al. 2018), and were prepared as previously described (Colón‐Rodríguez et al. 2020). Pooled gRNAs (4 μM total concentration) were microinjected into single cell NHGRI‐1 or transgenic zebrafish embryos to a volume of 0.5 nL/cell as previously described using a Pneumatic MPPI‐2 Pressure Injector (Jao, Wente, and Chen 2013). Scrambled injection RNP mix contained a single gRNA designed to have no target in the zebrafish genome. gRNA efficiencies were tested post‐injection using pooled genomic extractions of four embryos and PCR amplification of targeted loci followed by 7.5% polyacrylamide gel visualization (Uribe‐Salazar et al. 2022). These same amplicons were also subject to Illumina sequencing and the total alleles identified/quantified using the CrispRvariants R package (Figure S1, Table S1, and Data S1) (Lindsay et al. 2016).

Human mRNA was generated using cDNA plasmids (Horizon *YTHDF2*, MHS6278‐202827242; *GMEB1*, MHS6278‐202827172) (Gerhard et al. 2004) and prepared via the in vitro transcription kit mMESSAGE mMACHINE SP6 Transcription Kit (Thermo Fisher Scientific, AM1340). Mixes of 100 ng/μL mRNA and 0.05% phenol red were prepared, as previously described, and injected in single‐cell zebrafish embryos at a volume of 0.5 nL/cell (Yuan and Sun 2009).

### 2.5 Zebrafish Morphometric Measurements

Dorsal and ventral images of 3 days post fertilization (dpf) embryos were obtained using the Union Biometrica VAST Bioimaging System with LP Sampler via the built‐in camera and manufacturer settings (Pulak 2016). Zebrafish features were identified and quantified from VAST using FishInspector software 2.0 (Teixidó et al. 2019). FishInspector images were assessed for total area (contourDV_regionpropsArea), embryo length (contourDV_regionpropsLengthOfCentralLine), distance between the center of the eyes (YdistanceCenter_eye1DV_eye2DV), and telencephalon distance (YdistanceEdge_eye1DV_eye2DV). Statistical analysis was performed in R using the *ggsignif* package with the Wilcoxon test option (Gerhard et al. 2004; Ahlmann‐Eltze and Patil 2021).

Fluorescent images were acquired using the Andor Dragonfly High Speed Confocal Platform with the iXon Ultra camera. Human *YTHDF2*‐mRNA‐microinjected Tg(HuC:eGFP) strain zebrafish, which harbor a green‐fluorescent protein (GFP) fluorescent pan‐neuronal marker, were bathed in 0.003% 1‐phenyl‐2‐thiourea (PTU) in 10% Hank's saline between 20 and 24 hpf for 24 h (Fisher Scientific, 5001443999). At 3 dpf, zebrafish embryos were embedded in 1% low melt agarose (Thermo Fisher Scientific, BP160‐100) and Z‐stacks of 10 μm slices were taken across entire larval brains with a 20× objective lens and a GFP filter. Image processing was performed using Fiji by generating maximum intensity projections from hyperstacks with blinded quantification of total midbrain and forebrain area across all experimental groups.

### 2.6 Zebrafish RNA Extraction and RT‐qPCR

Whole zebrafish larvae were collected at 3 dpf and stored in 50 μL RNALater at −80°C until RNA extraction. Three biological replicate samples were prepared for both *ythdf2* KO and scrambled control larvae, each containing 15 larvae, for pooled RNA extraction using an RNeasy Plus Mini kit (Qiagen). Briefly, larvae were resuspended in 350 μL of the buffer RLT and vortexed until homogenized. Instructions from the RNeasy Plus Mini kit were followed for DNA Removal using the gDNA Eliminator column. Samples were quantified using a Qubit Broad Range kit and normalized to 4 ng/μL for RT‐qPCR following the instructions from the NEB Luna kit.

### 2.7 Single‐Cell Transcriptomics

Transcriptional differences across *YTHDF2* zebrafish models were assessed using single‐cell (sc)RNA‐seq. Cells were prepared from *ythdf2* knockdown and SpCas9‐scrambled‐gRNA‐injected controls as well as *YTHDF2*‐injected and eGFP‐mRNA‐injected controls. At 3 dpf, larval heads from each group were dissected after euthanasia in cold tricaine (0.025%), pooling 15 heads together per sample with three samples per group. Groups with low initial counts (*ythdf2* knockdown and eGFP‐mRNA) were repeated with an additional three samples. Cells from each sample were washed with 1 mL of cold 1× PBS twice and immediately incubated at 28°C in a mix of 480 μL of Trypsin–EDTA (0.25%) and 20 μL of Collagenase P (100 mg/mL) for a total of 15 min with gentle pipetting every 5 min to induce dissociation. To stop dissociation, 800 μL of cold DMEM with 10% FBS was mixed with each sample and immediately centrifuged at 4°C for 5 min at 700 g. The supernatant was carefully removed from the cell pellet and cells were washed in cold 1x PBS and centrifuged at 4°C for 5 min at 700 g, followed by another wash of cold DMEM with 10% FBS. Cells were then filtered into Eppendorf tubes using a P1000 pipette and a Flowmi 40 μm cell strainer (Sigma Aldrich, St. Louis, MO). Ten microliter of sample was then mixed with 10 μL of trypan blue solution and counted using a Countess II (Thermo Fisher, Waltham, MA) to record cell viability. All samples processed were confirmed to show viability above 70%.

Cell fixation and library preparation were performed following the sci‐RNA‐seq3 protocol (Cao and Shendure n.d.) using DSP/methanol. After the combinatorial indexing and PCR amplification steps, all wells were pooled together to ensure sufficient library yield before purification. The pooled libraries were then purified using AMPure XP beads to remove any remaining small fragments and primers. The quality and concentration of the libraries were assessed using a Bioanalyzer (Agilent Technologies, Santa Clara, CA) to ensure they met the required size distribution and concentration thresholds. Final libraries were size‐selected using the Pippin HT system (Sage Science, Beverly, MA). The target range was set to 400–500 bp, with the smear cut between approximately 300–600 bp to ensure that only fragments within this desired range were included. The libraries were sequenced with paired‐end read length of 150 bp using the Illumina NovaSeq 6000 platform (Novogene, Sacramento, CA).

FASTQ files were processed according to the sci‐RNA‐seq3 bioinformatic pipeline (https://github.com/JunyueC/sci‐RNA‐seq3_pipeline) and a comprehensive zebrafish transcriptome (Lawson et al. 2020) was used to generate cell‐by‐gene matrices per sample. These matrices were processed into Seurat objects using *Seurat* v5.0.3 (Hao et al. 2021). Cells with mitochondrial or ribosomal percentages above 5%, feature counts below 200 or over two standard deviations from the mean, and predicted doublets according to *DoubletFinder* (McGinnis, Murrow, and Gartner 2019) were removed from subsequent analyses. After quality‐control filtering, an average of 1126 cells per sample (4785 cells per group) were obtained and normalized with the 5000 most variable genes while regressing for ribosomal and mitochondrial percentages using *SCTransform*.

Samples were integrated using a reciprocal PCA reduction (Hao et al. 2021) and nearest‐neighbor graphs were made using the first 30 principal components with the *FindNeighbors* function for subsequent clustering. Hierarchical clustering was initially performed using the Euclidean distance between all cells from principal component embeddings with the tree cut at *k* = 10. Broad marker genes were assigned using the *PrepSCTFindMarkers* and *FindAllMarkers* functions using the wilcox test option (parameters: *logfc*.*threshold* = 0.1, *min.pct* = 0.2, *return.thresh* = 0.01, *only.pos* = TRUE). Brain cells from a single broad cluster were isolated and hierarchical clustering was similarly repeated with the tree cut at *k* = 18. Cell clusters were defined using defined marker genes cross‐referenced with larval zebrafish brain atlases (Zhang et al. 2021; Raj et al. 2020) and the zebrafish information network (ZFIN) (Bradford et al. 2022).

For the differential expression analysis, cells from the *ythdf2* knockdown and *YTHDF2*‐injected groups were randomly sampled with respect to our original cluster distribution to match control cell counts (*downsampleSeurat*). Differentially expressed genes (DEGs) were identified across all and a subset of brain cells, respectively, using the *FindMarkers* function with the wilcox test option (*logfc*.*threshold* = 0.1, *min.pct* = 0.01, *only.pos* = FALSE). GO analysis was performed on DEGs from *FindMarkers* (*p* value < 0.05 and *logfc* < 0.1) using *enrichGO* with a background universe of all expressed genes. A list of 842 high‐confidence Fragile X Syndrome (FXS) protein (FMRP) targets (Darnell et al. 2011) were converted to zebrafish orthologs using the g:Orth search from g:Profiler (Kolberg et al. 2023) to identify FMRP‐target DEGs (adjusted *p* value < 0.05) and assess enrichment using a Benjamini Hochberg (BH)‐adjusted Fisher's exact test. All other FMRP‐target genes expressed across both *ythdf2* knockdown and *YTHDF2* mRNA conditions in at least 0.01% of cells were selected for subsequent analysis (*n* = 675). *scCustomize* (Marsh n.d.) and *dittoSeq* (Bunis et al. 2021) were used for figure creation. Nebulosa (Alquicira‐Hernandez and Powell 2021) was used to visualize individual and joint expression from multiple FMRP‐DEGs using a kernel gene‐weighted density estimation.

## 3 Results

### 3.1 ASD‐DM Candidate Gene Discovery

ASD‐DM individuals were recruited through the UC Davis APP—a longitudinal study focused on the identification of ASD‐subphenotypes (Amaral et al. 2017; Nordahl et al. 2021; Ohta et al. 2016). Using MRI data from the study entry time point (2–3½ years of age) (Nordahl et al. 2021), we selected 11 individuals in the APP cohort that met the criteria for ASD‐DM, defined as a cerebral volume to height ratio > 1.5 standard deviations above the mean compared to typically developing age‐matched controls. Through a collaboration with MSSNG (Trost et al. 2022; Yuen et al. 2017), WGS and variant identification/annotation was performed for the autistic probands and a subset of family members, for which we also had blood specimens, including six trios and five non‐trio probands yielding over 200,000 variants. From this, we identified two exonic, *de novo*, likely gene‐disruptive variants from trio families, including one splice‐site variant impacting *RYR3* and one 109‐kbp duplication of *YTHDF2* and *GMEB1*. From the five individuals with no parental data, we identified a proband harboring a chromosome 1q21.1 microduplication, a CNV previously associated with ASD‐DM (Brunetti‐Pierri et al. 2008), and a single proband with variants in *CHD8* and *KMT2E* (Dolcetti et al. 2013). An additional 10 variants were found in non‐trio proband data to be exonic, likely gene‐disruptive, and rare (not previously recorded in dbSNP) (Sherry, Ward, and Sirotkin 1999) (Table S2). Of these, three impacted genes have SFARI scores of 3S or above (*KMT2E*, *RPS6KA5*, and *TTN*), an additional three genes have known neuronal functions (*DMBT1*, *IARS2*, *FGF12*), and one gene was found recurrently carrying variants in two probands (*SPANXN4*) (Abrahams et al. 2013).

**FIGURE 1:**
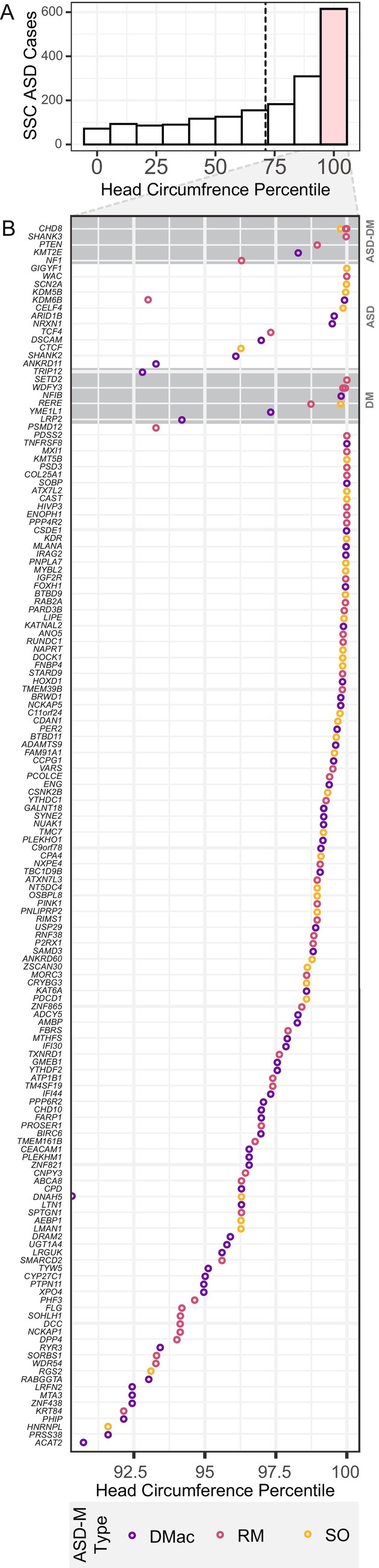
Macrocephaly level of candidate ASD‐DM and ASD‐M genes. (A) A histogram representing the number of SSC probands v. head circumference percentiles shows a skew toward larger head‐sizes compared to age‐ and sex‐matched typically developing children. The red bar designates those meeting the criteria for macrocephaly. The dashed line represents the distribution mean. (B) ASD‐DM and ASD‐M genes listed by their identified proband's head circumference percentiles show genes previously associated with ASD‐DM (first gray quadrant) are more likely to be associated with a higher head circumference percentiles than genes previously associated with autism (second white quadrant) and DM (third gray quadrant) alone. Color represents the macrocephaly type. DMac, disproportionate macrocephaly; RM, relative macrocephaly; SO, somatic overgrowth.

SSC consists of trios and quads of simplex autism families with accompanying genetic and phenotypic information. Due to the lack of MRI data for SSC participants, we used ASD‐M as a proxy for ASD‐DM. SSC head circumference and age data was used to determine ASD‐M status (head circumference > 1.5 standard deviations above the mean for typically developing sex‐ and age‐matched children) for 756 of 1847 SSC probands (40%) (Fischbach and Lord 2010) (Table S3, Figure S2). Considering only SNVs, indels, and CNVs, ASD‐M *de novo* likely gene‐disruptive variants were identified from published results (An et al. 2018; Trost et al. 2022), overlapping a total of 151 genes (Figure 1, Table S4). Of note, five genes were found recurrently mutated in ASD‐M, including *GALNT18*, *KDM6B*, *LTN1*, *RERE*, and *WDFY3*, as well as *CHD8*, which was disrupted in three probands.

In total, we identified 154 genes containing a likely gene‐disruptive variant across the APP ASD‐DM and SSC ASD‐M datasets. Rates of harboring a likely gene‐disruptive *de novo* variant in ASD‐DM probands were in line with previous predictions (19.4%) and nominally enriched compared to typically developing SSC siblings (16.5%), though not statistically significant (chi‐squared *p* value = 0.1) (Iossifov et al. 2014; Wu et al. 2020). Over a third of identified candidate genes (53/154) had a pLI score of > 0.9, suggesting intolerance to variation (Karczewski et al. 2020) (Table S4). Examining *de novo* missense variants, which have been shown to exhibit overall enrichments in affected probands versus unaffected siblings (Samocha et al. 2017; Koire et al. 2021), we did not observe an enrichment in our likely gene‐disruptive candidate genes in ASD‐M probands compared with their typically developing siblings (Fisher's exact *p* values = 0.59). However, we did find a statistically significant increase in *de novo* missense variants in our candidate genes when comparing ASD‐M to ASD‐without‐macrocephaly (ASD‐N) probands, siblings of ASD‐N probands, and between ASD‐N probands compared to their typically developing siblings (Fisher's exact *p* values = 0.03, 0.003, 0.02, respectively).

### 3.2 ASD‐DM Candidate Gene Network Analysis

**FIGURE 2:**
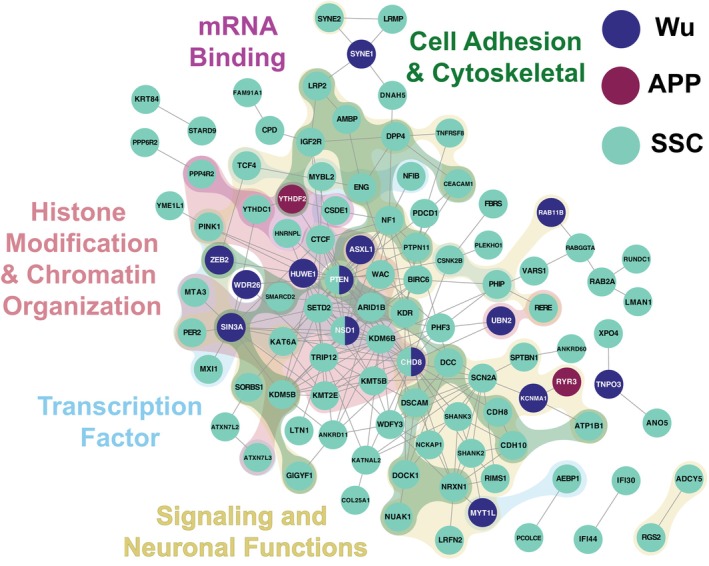
Network analysis and gene ontology (GO) of ASD‐DM candidate genes. ASD‐DM candidate genes from SSC (teal), APP (purple), and Wu (navy) probands are connected in a network via active interactions as determined by STRING (Szklarczyk et al. 2015). Background colors represent shared GO molecular functions. Disconnected gene nodes are not included.

Identifying shared patterns of molecular functions and ontologies of impacted ASD‐DM genes may point to additional gene candidates. Due to the highly heterogeneous nature of ASD, this type of analysis expands our ability to identify disrupted biological mechanisms and spatio‐temporal expression patterns implicated in autism (Willsey et al. 2013). Here, we used as seeds 167 previously known and identified‐in‐this‐study ASD‐DM genes to identify active interactions using the STRING database (Figure 2) (Wu et al. 2020; Szklarczyk et al. 2015). This analysis uncovered ontology groups enriched in our dataset previously reported for ASD, including proteins involved in histone modification and chromatin organization, transcription factors, cell signaling (e.g., SMAD and E‐box binding), functions key to neuronal activity (e.g., synapse assembly and excitatory postsynaptic potential), cell adhesion and cytoskeletal proteins, and mRNA binding (Table S5) (Lasalle 2013; Hoffmann and Spengler 2021; Pinto et al. 2014; Brooks‐Kayal 2010). Out of our original ASD‐DM candidate seed genes, 55.6% (93/167) fall under one of these ontologies.

We next used the database for annotations, visualization, and integrated discovery (DAVID) to identify unique ontologies enriched in the 167 known and candidate ASD‐DM genes compared to ontologies enriched in SSC ASD‐N proband likely gene‐disruptive genes, versus genes previously associated with DM (Sherman et al. 2022; Pirozzi, Nelson, and Mirzaa 2018). While there are many commonalities between the subphenotype and ASD‐N, including histone methyltransferase activity, ASD‐DM is uniquely enriched for terms such as autism spectrum disorder, chromatin remodeling, and cytoskeletal structure (spectrin repeats) (Table S5).

To identify putative additional candidate ASD‐DM genes, we expanded our network to include the top 10 interactors for each ASD‐DM seed gene defined by STRING as having known protein interactions, shared homology, and co‐expression patterns (Szklarczyk et al. 2015) (Table S6). Of the ASD‐DM candidate gene interactors, 52.4% (963/1837) fall under one of the ontologies found in our ASD‐DM network. Interestingly, one of these genes has previously been associated with both autism and DM individually, *PIK3CA* (Table S7). PIK3CA functions as a catalytic subunit of the mTOR pathway and has previously been found to be associated with developmental delay and DM, including one individual diagnosed with autism (Yeung et al. 2017).

In this ASD‐DM interactor set, 21 genes are high‐confidence autism genes not previously associated with DM (*ANK2*, *ASXL3*, *CTNNB1*, *CUL3*, *DLG4*, *DYRK1A*, *GNAI1*, *GRIN2B*, *KCNMA1, KMT2A*, *NCOA1*, *NIPBL*, *NLGN1, NRXN1*, *PHF12*, *POGZ*, *PPP1R9B*, *SIN3A, SMARCC2*, *TBL1XR1*, *UBR1*). Eighteen additional genes from this interactor set have been implicated in DM and as SFARI putative autism candidate genes (*ANK3*, *CHD2*, *CHD3*, *FRMPD4*, *HCFC1*, *HDAC4*, *HRAS*, HUWE1, *PAK1*, *PIK3R2*, *RAC1*, *SETD1A*, *SLC25A1*, *SMAD4*, *TBL1X*, *TRIO*, *USP7*, and *USP9X*), and 189 more have been implicated in DM or have a SFARI score. Especially promising are the 21 genes that contain missense variants in the SSC ASD‐M probands, but not in ASD‐N probands or their typically developing siblings, including *ABI2*, *ANK3*, *SRC*, *SRCAP*, *ATP12A*, *BAIAP2*, *CHD13*, *CH815*, *FGG*, *JUP*, *KDM2A*, *KIF20A*, *MAPK8*, *PDGFRB*, *RING1*, *SCN4A*, *SHANK1*, *SMC3*, *TCF3*, *WDR5*, and *ZC3H3*. Together, network analysis and ontology point to these genes as promising ASD‐DM candidate genes going forward.

### 3.3 Modeling a *YTHDF2* Duplication Identified in an ASD‐DM Proband Using Zebrafish

**FIGURE 3:**
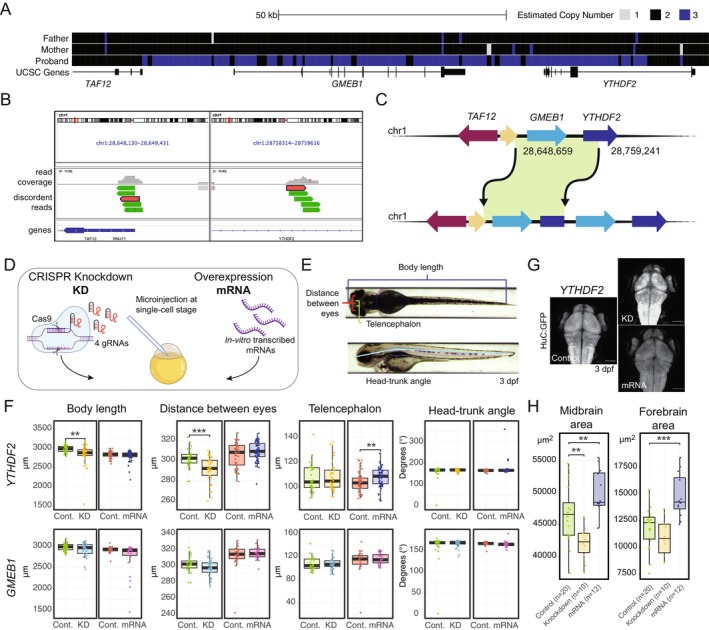
Disrupting *ythdf2* in zebrafish is associated with head and brain size phenotypes. (A) Copy‐number‐estimate plot (QuickMer2) using sequencing data from the APP proband harboring a *de novo* 109‐kb duplication on chromosome 1 compared with their parents harboring two diploid copies. (B) IGV plot showing discordant reads in the APP proband supporting a tandem duplication. (C) An illustration of the tandem duplication on chromosome 1 in an APP proband encompassing *GMEB1* and all but the last exon of *YTHDF2*. (D) Cartoon depicting the CRISPR‐based knockdown (KD) and overexpression using in vitro transcribed mRNAs (mRNA) experimental paradigms by injection of nucleic acid into single‐cell zebrafish embryos. (E) Morphometric measurements were produced using VAST platform images and automated feature extraction via FishInspector (Teixidó et al. 2019) of body length, distance between the eyes, telencephalon width, and head‐trunk angle. (F) Features were quantified in 3 dpf larvae by comparing *ythdf2* knockdown (KD, *n* = 37) versus scrambled gRNA controls (Cont., *n* = 34) and *YTHDF2* overexpression (mRNA, *n* = 55) versus injection controls (Cont., *n* = 55) (top). Similar comparisons were made for *gmeb1* KD (*n* = 33) versus scrambled gRNA controls (*n* = 34) and *GMEB1* mRNA (*n* = 26) vs. injection controls (*n* = 26) (bottom). (G) Knockdown and mRNA injected zebrafish harboring a pan neuronal marker (HuC:eGFP) reveal brain size differences at 3 dpf. Representative control, knockdown, and mRNA injected zebrafish images of transgenic larvae. Scale bar is 100 μm. (H) *ythdf2* knockdown embryos show significantly decreased midbrain volume (Wilcoxon *t* test, *p* value = 0.006). *YTHDF2* mRNA‐injected embryos show both significantly increased midbrain (Wilcoxon *t* test, *p* value = 0.014) and forebrain (Wilcoxon *t* test, *p* value = 0.001). All *p* values are adjusted for multiple‐testing using Bonferroni correction and only significant comparisons depicted as: ≤ 0.05*, ≤ 0.01**, ≤ 0.001***.

To narrow in on ASD‐DM genes contributing to a head‐size phenotype, we generated CRISPR knockout F_0_ embryos (or “crispants”) via microinjection of four gRNAs targeting exonic regions of seven candidate genes (*RYR3*, *GMEB1*, *YTHDF2, IARS2*, *RPS6KA, CHD8*, and *FAM91A1*; Figures S1, S3, and S4). This approach has been shown to result in near complete mosaic knockout of genes with little off‐target effects (Wu et al. 2020; Colón‐Rodríguez et al. 2020; Kroll et al. 2021). At 3 dpf, *ythdf2* crispants exhibited the most obvious morphological differences compared with negative scrambled‐gRNA injection controls (Wilcoxon *t* test *p* values < 0.01). The APP ASD‐DM proband carried a *de novo* 109‐kbp duplication harboring the entire *GMEB1* gene and the first five of six exons of *YTHDF2* that we verified using sequence read depth (QuicK‐mer2) (Thorvaldsdóttir, Robinson, and Mesiro 2013; Shen and Kidd 2020) (Figure 3A). Split reads falling at the identified breakpoints indicated that the duplication was inserted in tandem at the 3′ untranslated region (UTR) of the noncoding divergent transcript of *TAF12*, directly upstream of *GMEB1* (Figure 3B,C). Using available microarray data produced from mRNA derived from whole venous blood, we found that both *GMEB1* and *YTHDF2* exhibited increased expression > 3 standard deviations from the mean in the APP proband harboring the duplication compared to other APP participants (Stamova et al. 2013).

*GMEB1* is an auxiliary factor in parvovirus replication known to inhibit apoptosis in neurons and previously associated with schizophrenia (Nakagawa et al. 2008; Singh et al. 2022), and *YTHDF2* is a member of the m^6^A‐containing mRNA degradation complex known to be downregulated in neuronal fate determination (Sokpor et al. 2021), making both of these attractive potential ASD‐DM candidate genes. Based on their known functions, duplication of either gene could plausibly result in neurodevelopmental effects. Therefore, we quantified gross morphometric features, including body length as well as distance between the center of the eyes and telencephalon width (as proxies for head size), of *ythdf2* and *gmeb1* knockdown crispants, respectively (Figure 3D–F). *ythdf2* crispants exhibited significantly reduced body length (*p* value = 0.004, ~0.96 fold change) and distance between the eyes (*p* value = 0.001, ~0.97 fold change) versus scrambled‐gRNA controls. Measuring the head‐trunk angle, an indicator of developmental timing (Kimmel et al. 1995), resulted in no significant differences in crispants versus controls suggesting the observed features are not a product of developmental delay. We also modeled increased expression of *YTHDF2* and *GMEB1* by microinjecting human in vitro transcribed mRNA into single‐cell stage zebrafish embryos compared with dye‐injected controls. For *YTHDF2*, counter to the overall smaller features observed in *ythdf2* crispants, we observed increased telencephalon width compared to dye‐injection controls (*p* value = 0.005, ~1.05 fold change) consistent with increased head size but no significant differences to body length, matching the proband phenotype. No obvious morphological features were impacted in *GMEB1* crispant or overexpression larvae (Figure 3F).

As we encountered some inconsistencies in head size measurements between *ythdf2* knockdown and *YTHDF2* mRNA models when using distance between the eyes versus telencephalon, we sought to measure brain size directly. To do this, we repeated the experiment in the zebrafish transgenic line HuC:eGFP (Park et al. 2000), which harbors a green‐fluorescent protein (GFP) pan‐neuronal marker (Figure 3G,H). These embryos displayed brain size differences, with *ythdf2* knockdown embryos showing significantly reduced midbrain, and *YTHDF2* mRNA injected “overexpression” embryos exhibiting significantly increased midbrain and forebrain compared to injection controls. Together, the knockdown and mRNA overexpression zebrafish models provide evidence that increased dosage of *YTHDF2* is associated with DM while its loss leads to microcephaly.

### 3.4 Transcriptomic Impacts of *YTHDF2* Dosage in Zebrafish

**FIGURE 4:**
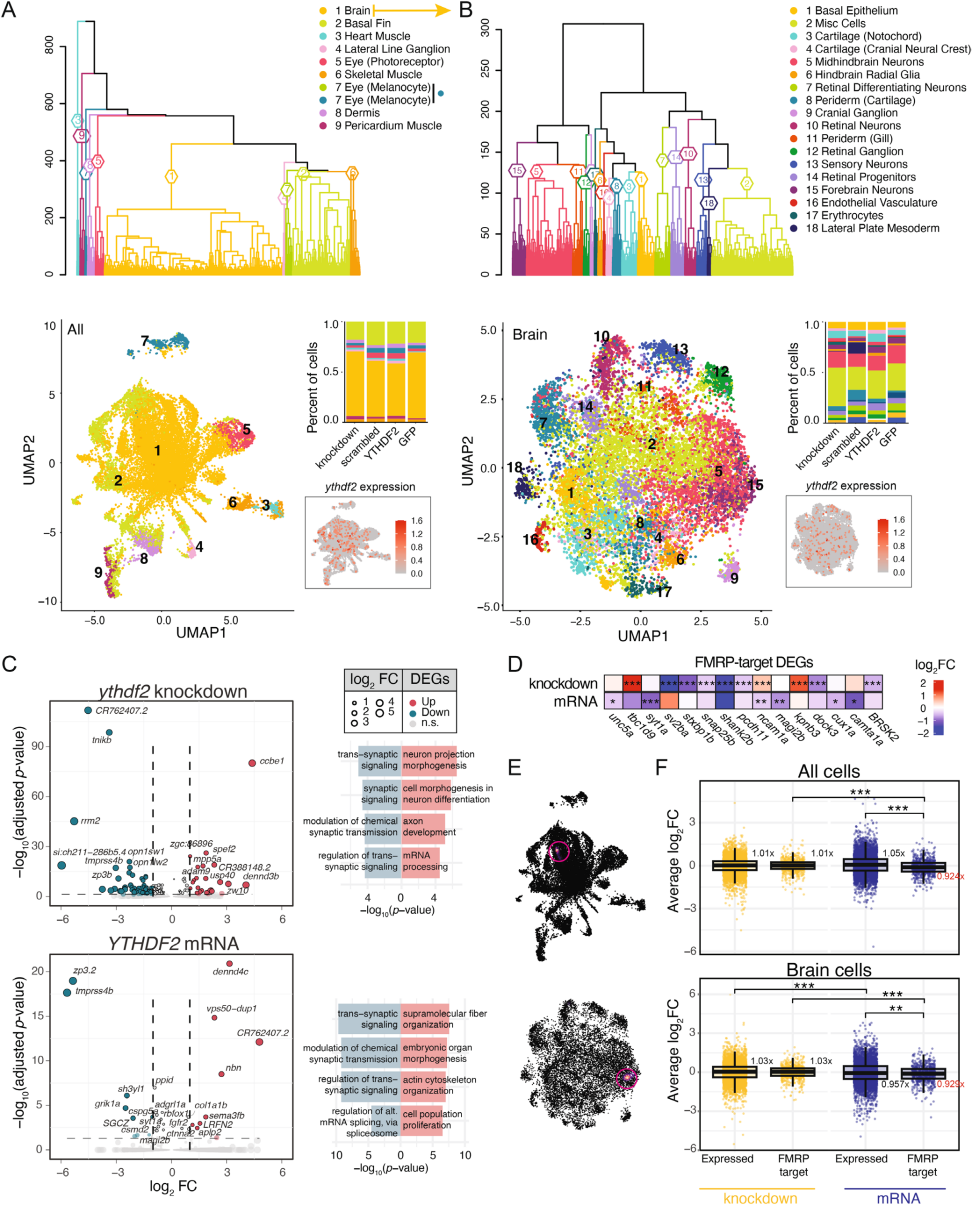
Single‐cell transcriptomes of *YTHDF2* zebrafish models. (A) Hierarchical clustering of 19,141 cells across *ythdf2* knockdown crispant and YTHDF2 mRNA overexpression models and associated controls (scramble gRNA and eGFP mRNA) into broad cell types based on the expression of gene markers. Dendrograms were created to cluster cells with similar expression profiles and visualized via UMAP plots, with colors indicating assigned cluster IDs. The proportion of cells assigned to each cluster per model is a percentage of total cells indicated as a barplot. Cells with *ythdf2* transcripts are colored red based on a continuous scale of natural log normalized expression (Hao et al. 2024). (B) Hierarchical sub‐clustering of 12,066 brain cells across all conditions into 18 cell types based on the expression of gene markers. Proportion of cells and *ythdf2* expression are also plotted, as described in (A) for the brain sub cluster. (C) Volcano plots showing DEGs across all cell‐types within *ythdf2* knockdown (top) and *YTHDF2* mRNA overexpression (bottom) models relative to controls, with fold change (FC) plotted versus adjusted *p* value. DEGs with absolute log_2_FC ≥ 1 and adjusted *p* value ≤ 0.05 are colored (upregulated as red, downregulated as blue). A subset of significantly enriched gene ontologies (adjusted *p* value ≤ 0.01) are depicted as bar plots for upregulated and downregulated DEGs next to each respective volcano plot for knockdown and overexpression models. (D) Average log_2_FC for 15 significant FMRP‐target DEGs identified in knockdown or overexpression models with respect to controls are shown. (E) Joint kernel density estimation was calculated from all 15 FMRP‐target DEGs (*Nebulosa*) highlighting higher expression within a sub‐type of brain cells. (F) The average log_2_FC per expressed gene (0.01% of cells) was plotted across all cells and brain cells (see Section 2) for all and 675 FMRP‐target genes expressed in both groups for the *ythdf2* knockdown and *YTHDF2* mRNA zebrafish models. Comparisons were made using *t* tests either paired (between models) or unpaired (within models). Median fold changes versus respective controls (scrambled for knockdown and eGFP for mRNA) are indicated next to plots. All *p* values in this figure are represented as: ≤ 0.05*, ≤ 0.01**, < 0.001***.

Exploring the *YTHDF2* models further, we verified gene knockdown in our crispant larvae, showing a significant ~0.37 fold change (FC) in *ythdf2* expression versus scrambled gRNA controls through quantitative RT‐PCR analysis (*p* value < 0.001; Figure S5). To better characterize neurodevelopmental phenotypes, we next performed sci‐RNA‐seq (Cao et al. 2019) of knockdown and overexpression models at 3 dpf, profiling 19,141 single cells from mechanically isolated heads (average of 4785 cells per group or 1126 cells per biological replicate; Table S8). Using known marker genes (Zhang et al. 2021; Raj et al. 2020), we identified nine broad clusters and observed widespread localization of *ythdf2* across cell types (Figure 4A, Figure S6, Table S9). This agrees with its reported general expression in humans (GTEx Consortium 2020) and zebrafish (Kontur et al. 2020; Yang et al. 2020) (Figure S7), which begins early in development at 3 hpf (White et al. 2017). We further sub clustered 12,066 cells classified as brain into 18 cell types, again showing broad *ythdf2* expression (Figure 4B, Figure S6, Table S9).

Differential pseudo‐bulk gene expression analysis across all cells (with counts balanced relative to controls, see Section 2) revealed 131 significant DEGs in our *ythdf2* knockdown and 33 DEGs in the *YTHDF2* mRNA overexpression model versus respective controls (adjusted *p* value cutoff = 0.05, log_2_FC cutoff = 0.1; Figure 4C, Table S10). Significant enrichment (adjusted *p* value < 0.01) of GO terms related to synaptic signaling was found for downregulated DEGs in both knockdown and mRNA models. Upregulated DEGs in *ythdf2* knockdown were enriched in functions related to morphogenesis of differentiated neurons, including neuron projections and axon development. Conversely, *YTHDF2* mRNA upregulated DEGs were enriched for terms related to cell proliferation and actin cytoskeleton organization. Interestingly, DEGs related to mRNA processing were found for both models but in opposite directions (up for the knockdown and down for mRNA overexpression larvae); examples include *rbfox1*, *nova2*, and *celf4* with human orthologs implicated in neurodevelopmental conditions (*CELF4* mutated in our ASD‐M cohort; Figure 1) (O'Leary et al. 2022; Salamon et al. 2023; Halgren et al. 2012; Mattioli et al. 2020). We identified fewer significant DEGs when considering only brain cells (*ythdf2* knockdown *n* = 94 and *YTHDF2* mRNA *n* = 12; Table S10) resulting in no significant GO enrichments for either model (adjusted *p* value < 0.01; Table S11).

Recent studies have suggested that FMRP and YTHDF2 compete for binding to m^6^A‐methylated RNA targets impacting their stability (Darnell et al. 2011; Shu et al. 2020). Considering significant DEGs identified from all cells in both our models, we observed an enrichment of high‐confidence Fragile X Syndrome (FXS) protein (FMRP) targets (3.6% expected versus 7.2% observed enrichment of DEGs considering 842 target genes; Fisher's exact test BH‐adjusted *p* value = 0.008; Figure 4D). This list includes genes with known functions in neurodevelopment (*ncam1a*, *cux1a*, *unc5a*, *tbc1d9*, *camta1a*, *magi2b*, *syt1a*, *sv2bc*, and *stxbp1b)*. Overlapping expression of the 15 FMRP‐target DEGs through joint density profiles shows strongest expression in forebrain and midbrain cells (Figures 4E and S8). Considering all FMRP‐target genes, we observed significantly reduced expression in *YTHDF2* mRNA compared with *ythdf2* knockdown larvae considering all cells (*p* value = 2.4 × 10^−7^, Figure 4F) and only brain cells (*p* value = 3.2 × 10^−6^, Figure 4F). These combined results are consistent with previous studies implicating YTHDF2 as preferentially binding to FMRP target genes resulting in mRNA degradation and global downregulation.

## 4 Discussion

The autism sub‐phenotype ASD‐DM, which occurs in approximately 15% of autistic boys, is associated with lower language ability at age three and slower gains in IQ across early childhood resulting in a higher proportion with IQs in the range of intellectual disability by age six (Amaral et al. 2017). Clues at the underlying etiology of ASD‐DM can be found in high‐confidence genes such as *CHD8*, a chromatin remodeler important in early brain development (Bernier et al. 2014; Weissberg and Elliott 2021), and *PTEN*, a tumor suppressor gene that functions in cell proliferation (Klein, Sharifi‐Hannauer, and Martinez‐Agosto 2013). While variants impacting these two genes alone are estimated to contribute in up to 15% of all ASD‐M cases (Wu et al. 2020), a majority remain unsolved. Here, we examined the genomes of 766 ASD‐DM and ASD‐M trios and quads from the APP and SSC cohorts to identify 154 ASD‐DM candidate genes containing *de novo* likely gene‐disruptive variants. Ontologies of affected genes largely matched those previously implicated in ASD (Moyses‐Oliveira et al. 2020).

When compared with genes implicated in ASD‐N and DM‐alone, functions related to autism spectrum disorder, histone methyltransferase activity, and cytoskeletal structure stand out in ASD‐DM alone (Table S5). To further disentangle mechanisms shared and unique to ASD‐DM, we collectively categorized the identified genes from our study and previously published (Wu et al. 2020) as high‐confidence ASD‐DM (*n* = 5), ASD‐N (*n* = 14), and DM‐alone (*n* = 7), as well as those with uncertain disease relevance (*n* = 128) (Table 1). Perhaps unsurprisingly, 16% of high‐confidence disease risk genes exhibit recurrence in our cohort, including *CHD8* with three probands affected, while only two genes (*GALNT18* and *LTN1*) in the uncertain “Other” category. These latter genes represent compelling ASD‐DM risk candidates, with *GALNT18* (Polypeptide *N*‐Acetylgalactosaminyltransferase 18) functioning in O‐linked glycosylation, and *LTN1* (Listerin E3 Ubiquitin Protein Ligase) encoding a RING‐finger protein and E3 ubiquitin ligase (Bernier et al. 2014; Stolerman et al. 2019; Fregeau et al. 2016; Le Duc et al. 2019; Coit et al. 2020; Doamekpor et al. 2016).

**TABLE 1:** Candidate ASD‐DM genes.

Category	Candidate genes
ASD‐DM (*n* = 5)	*CHD8* ^3^, *KMT2E* ^ASD‐N^, *NF1* ^DP^, *PTEN* ^DP^, *SHANK3* ^DP^
ASD (*n* = 14)	*ANKRD11* ^ASD‐N^, *ARID1B* ^ASD‐N^, *CELF4*, *CTCF*, *DSCAM* ^DP, ASD‐N^, *GIGYF1* ^DP, ASD‐N^, *KDM5B* ^DP^, *KDM6B* ^2, DP^, *NRXN1* ^DP^, *SCN2A* ^DP, ASD‐N^, *SHANK2*, *TCF4* ^DP^, *TRIP12*, *WAC* ^DP, ASD‐N^
DM (*n* = 7)	*LRP2* ^DP^, *NFIB* ^NO^, *PSMD12*, *RERE* ^2, DP^, *SETD2*, *WDFY3* ^2^, *YME1L1* ^DP^
Other (*n* = 128)	*ABCA8* ^DP^, *ACAT2*, *ADAMTS9*, *ADCY5* ^ASD‐N^, *AEBP1* ^DP^, *AMBP*, *ANKRD60* ^NO^, *ANO5* ^DP^, *ATP1B1* ^DP, ASD‐N^, *ATXN7L2* ^DP^, *ATXN7L3*, *BIRC6*, *BRWD1*, *BTBD11* ^DP^, *BTBD9*, *C11orf24*, *C9orf78*, *CAST*, *CCPG1*, *CDAN1*, *CDH10* ^DP^, *CEACAM1*, *CNPY3*, *COL25A1*, *CPA4* ^DP^, *CPD* ^DP^, *CRYBG3* ^NO^, *CSDE1*, *CSNK2B*, *CYP27C1*, *DCC*, *DNAH5*, *DOCK1*, *DPP4*, *DRAM2* ^DP^, *ENG* ^DP^, *ENOPH1*, *FAM91A1*, *FARP1*, *FBRS*, *FLG* ^NO^, *FNBP4*, *FOXH1*, *GALNT18* ^2, DP^, *GMEB1*, *HIVEP3* ^DP^, *HNRNPL*DP, *HOXD1*, *IFI30* ^DP^, *IFI44* ^DP^, *IGF2R*, *KAT6A*, *KATNAL* ^DP^, *KDR*, *KMT5B*, *KRT84* ^DP^, *LIPE* ^DP^, *LMAN1*, *LRFN2* ^DP^, *LRGUK*, *LRMP*, *LTN1* ^2^, *MLANA* ^NO^, *MORC3* ^DP^, *MTA3*, *MTHFS* ^DP^, *MXI1*, *MYBL2* ^DP^, *NAPRT*, *NCKAP1* ^ASD‐N^, *NCKAP5* ^NO^, *NT5DC4*, *NUAK1* ^DP^, *NXPE4* ^DP^, *OSBPL8*, *P2RX1*, *PARD3B* ^DP^, *PCOLCE* ^DP^, *PDCD1* ^NO^, *PDSS2*, *PER2*, *PHF3*, *PHIP*, *PINK1*, *PLEKHM1*, *PLEKHO1* ^DP^, *PNLIPRP2* ^NO^, *PNPLA7* ^DP^, *PPP4R2* ^DP^, *PPP6R2* ^DP^, *PROSER1*, *PRSS38* ^NO^, *PSD3*, *PTPN11* ^DP^, *RAB2A*, *RABGGTA*, *RGS2*, *RIMS1* ^DP, ASD‐N^, *RNF38*, *RUNDC1*, *RYR3*, *SAMD3* ^NO^, *SMARCD2*, *SOBP* ^DP^, *SOHLH1* ^NO^, *SORBS1*, *SPTBN1*, *STARD9*, *SYNE2* ^DP^, *TBC1D9B*, *TM4SF19* ^DP^, *TMC7* ^NO^, *TMEM161B*, *TMEM39B*, *TNFRSF8* ^NO^, *TXNRD1*, *TYW5*, *UGT1A4* ^DP^, *USP29* ^DP^, *VARS*, *WDR54*, *XPO4*, *YTHDC1*, *YTHDF2*, *ZNF438*, *ZNF821*, *ZNF865*, *ZSCAN30* ^NO^

*Note*: ^2,3^ number of occurrences in cohort, if more than 1.

Abbreviations: ASD‐N, LoF variant in ASD‐N SSC proband; DP, duplicate paralogs in zebrafish; NO, no ortholog in zebrafish.

As 43% of *de novo* likely gene‐disruptive variants in probands have been estimated to contribute to an autism diagnosis, we anticipate that many of the genes identified in this study contribute to ASD‐DM or autism in general (Iossifov et al. 2014). Nevertheless, only a single variant was discovered for a majority of candidates limiting our ability to narrow in on true causal genes. As a result, we tested the functions of a subset of candidate genes in zebrafish neurodevelopment and narrowed in on *ythdf2*, with CRISPR‐mediated loss‐of‐function leading to smaller brain size. To appropriately model the identified patient *YTHDF2* duplication for which we hypothesize gene gain‐of‐function effects, we overexpressed human *YTHDF2* in zebrafish recapitulating both increased head and brain sizes (Figure 3). To our knowledge, *YTHDF2* gain‐of‐function has not previously been characterized in vivo. Alternatively, published knockout models of the gene in mice (Li et al. 2018a) and zebrafish (Zhao et al. 2017) using TALENs and morpholinos that target maternal *ythdf2* transcripts exhibit severe phenotypes and large rates of embryonic death. Generally, the gene exhibits considerable functional constraint between species (e.g., 95% homology with mouse and 72% with zebrafish) and across hundreds of thousands of sequenced humans, with a significant depletion of likely gene‐disruptive SNVs discovered to date (gnomAD pLI score of 1, LOUEF score 0.132) (Karczewski et al. 2020). Further, assessment of individuals from the 1000 Genomes Project (*n* = 2504), SSC families (*n* = 9068), and gnomAD (*n* = 464,297) did not identify any CNVs impacting *YTHDF2*. Combined, these results highlight the rarity of the 109‐kbp duplication impacting *YTHDF2*, identified in a single ASD‐DM proband, and suggests that variants impacting this gene could plausibly lead to disease pathogenicity and/or lethality.

In addition to its high conservation, *YTHDF2* is a feasible contributor to ASD‐DM based on its previously implicated functions in neurodevelopment (Li et al. 2018a, 2018b). The encoded protein exists in the cytoplasm where it designates m^6^A‐labeled transcripts for degradation (Wang et al. 2014) through the recruitment of protein complexes that deadenylate and de‐cap mRNA (Wang et al. 2014; Du et al. 2016). It has over 3000 target transcripts, including some previously associated with ASD such as *CREBBP* (Sokpor et al. 2021). Studies using induced pluripotent stem cells show that the gene is required for neuronal fate determination, with *YTHDF2* knockdown leading to delayed mitotic entry (Heck et al. 2020; Fei et al. 2020) and inhibited pluripotency (Wu et al. 2019). Conditional knockout mouse models of *Ythdf2* exhibit decreased cortical thickness as a result of reduced neurogenesis in early development (Li et al. 2018a). Together, these results suggest that *YTHDF2* duplication leads to delayed neuronal fate determination, resulting in an overabundance of neuronal progenitor stem cells followed by increased neurogenesis.

While functions of *YTHDF2* in cell cycle and proliferation mechanistically associate it with brain‐size phenotypes, evidence of its interactions with mRNA‐binding FMRP provide plausible connections with ASD. FXS, caused by loss of FMRP function, represents the most common single‐gene cause of ASD, accounting for 2%–6% of diagnosed cases (Kaufmann et al. 2017). Interestingly, FXS has a similar increased prevalence of macrocephaly as in ASD (Sacco, Gabriele, and Persico 2015; Hazlett et al. 2012). Several studies have shown FMRP preferentially binds modified RNAs through recognition of m^6^A consensus motifs resulting in protection of transcripts from YTHDF2‐mediated degradation (Zhang et al. 2018, 2022; Hsu et al. 2019), possibly through direct interactions of the two proteins. This is evident in mouse neuroblastoma cells, where the loss of Fmrp is associated with reduced m^6^A‐modified transcripts, while knockdown of *Ythdf2* leads to increased stability and longer half lives of modified RNAs (Zhang et al. 2018).

**FIGURE 5:**
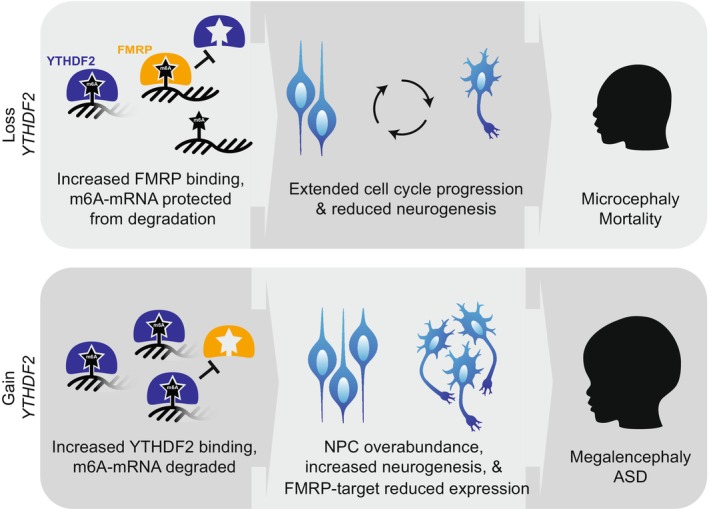
A potential role for *YTHDF2* in ASD‐DM. Proposed model of *YTHDF2* loss‐ or gain‐of‐function phenotypes, with respect to FXS protein FMRP. We hypothesize *YTHDF2* loss‐of‐function would lead to microcephaly due to increased FMRP binding and lack of m^6^‐mRNA degradation, extended cell cycle progression, and reduced neurogenesis. As the gene is highly conserved and knockout models are embryonic lethal, likely loss‐of‐function mutations in humans lead to disease pathogenicity or are incompatible with life. Inversely, *YTHDF2* duplication would lead to megalencephaly following increased m^6^A‐mRNA degradation as YTHDF2 outcompetes FMRP, neural progenitor cell (NPC) overabundance, and increased neurogenesis.

In our transcriptomic analysis of *YTHDF*2 zebrafish models, we found significant enrichment of FMRP‐target DEGs when considering both knockdown and mRNA‐overexpression larvae, with several genes exhibiting opposing effects in knockdown versus overexpression (Figure 4D). One example is *ncam1* (Neural adhesion molecule 1), where we observed significant upregulation in *ythdf2*‐knockdown and downregulation in *YTHDF2*‐mRNA larvae; this is in line with several studies in humans connecting depressed *NCAM1* expression with ASD (Plioplys, Hemmens, and Regan 1990; Purcell et al. 2001; Gomez‐Fernandez et al. 2018; Yang et al. 2019). Further, we show that overexpressing *YTHDF2* in zebrafish is associated with decreased expression of FMRP‐target genes across all cells and in the brain (Figure 4F), likely due to increased degradation of m^6^A‐modified mRNAs. Based on these collective findings, we propose a model in which *YTHDF2* loss‐of‐function results in microcephaly and possibly mortality, by increasing stability of m^6^A‐labeled RNAs resulting in extended cell‐cycle progression and a reduction in neurogenesis (Figure 5). Alternatively, *YTHDF2* gain‐of‐function results in increased degradation of m^6^A‐modified transcripts, possibly contributing to megalencephaly through increased neurogenesis and ASD through reduced FMRP‐target gene transcripts (Flanagan et al. 2022).

Expanding beyond *YTHDF2*, we identified an additional *de novo* likely gene‐disruptive variant in an SSC ASD‐M proband impacting another YTH‐domain‐containing m^6^A‐RNA reader, *YTHDC1*. The encoded protein promotes recruitment of splicing factors and facilitates nuclear export of modified transcripts (Xiao et al. 2016). Where gain of *YTHDF2* may lead to a decrease in overall m^6^A‐mRNA by promoting transcript degradation, conversely the loss of *YTHDC1* would likely result in a depletion of spliced and cytoplasmic transcripts (Gokhale and Horner 2017). Interestingly, FMRP also facilitates nuclear export of m^6^A‐labeled RNAs prevalent during neural differentiation (Hsu et al. 2019; Edens et al. 2019; Kim, Imam, and Siddiqui 2021). While m^6^A‐RNA regulation genes were not significantly enriched in our identified ASD‐DM candidate genes, none were observed in SSC ASD‐N or in typically developing siblings (Jiang et al. 2021), suggesting that additional genes within this pathway may contribute to ASD‐DM. Indeed, FMRP has been shown to repress translation of m^6^A RNAs, through competition for binding and inhibition of m^6^A reader *YTHDF1* (Zou et al. 2023). These ties between m^6^A mRNA readers and FMRP suggest a fine balance of select transcripts, in which up‐ or down‐regulation may impact early neurodevelopment and autistic phenotypes. Further, the m^6^A‐labeled mRNA flavivirus ZIKA is associated with severe congenital microcephaly, with *YTHDF2* found to bind and destabilize viral RNA (Lichinchi et al. 2016; Gokhale et al. 2020). These antiviral functions are controlled in part by the *METTL3* methyltransferase, which labels viral RNA for degradation, and whose knockout models are also associated with a reduced brain size in mice (Wang et al. 2018). Together the m^6^A‐mRNA pathway—including YTH‐domain proteins and m^6^A de/methyltransferases—represents a compelling future area of study in regard to ASD and brain‐size phenotypes.

Though new insights were achieved from our study, we would like to highlight some limitations. Due to the dearth of MRI evidence, not all SSC ASD‐M probands included in this study will meet the criteria for ASD‐DM. We do expect the overlap to be significant, as macrocephaly has previously been found to be highly correlated with megalencephaly, with an increased correlation in young children (Bartholomeusz, Courchesne, and Karns 2002). This is supported by the majority of APP ASD‐DM probands also meeting the criteria for macrocephaly (82%). Additionally, zebrafish, with a forebrain that most closely resembles the mammalian neocortex (Cheng, Jesuthasan, and Penney 2014), may not be a suitable model for all ASD‐DM candidate genes. Nevertheless, characterizing hundreds of genes implicated in ASD and other neurodevelopmental conditions in zebrafish has been successfully demonstrated (reviewed by Veenstra‐VanderWeele et al. 2023; Rea and Van Raay 2020; Tayanloo‐Beik et al. 2022; Dreosti et al. 2020; Sakai, Ijaz, and Hoffman 2018).

Overall, this study represents a significant increase in the number ASD‐DM and ASD‐M proband genomes analyzed in search of candidate genes. The 154 candidate genes introduced here greatly expand our knowledge of the genetic factors specifically contributing to this severe subphenotype of ASD. With this list, network analysis can now be leveraged to identify additional candidate genes with similar gene functions to known ASD‐DM genes. Our study introduces a novel ASD‐DM candidate gene *YTHDF2* connected with head‐size phenotypes in a zebrafish model system and 142 novel unvalidated ASD‐DM candidate genes (Table 1). Finally, our research highlights zebrafish as a viable model in performing functional characterization of putative risk genes.

## Disclosure

This study involves no clinical trials.

## Conflicts of Interest

The authors declare no conflicts of interest.

## Supporting information


Figures S1–S8.

Data S1.

Tables S1–S11.

## Data Availability

Raw sequencing data of patients, including FASTQ and VCF files, can be accessed through the MSSNG access agreement (https://research.mss.ng) and the Simons Simplex Collection through SFARI Base (https://www.sfari.org/resource/sfari‐base/). Transcriptomic data from zebrafish mutants is available through the European Nucleotide Archive (Accession number PRJEB83709).
