# Supplementary material for: m6A‐mRNA Reader YTHDF2 Identified as a Potential Risk Gene in Autism With Disproportionate Megalencephaly
Source: Autism Res. 2025 Jan 30;18(5):966–82. doi: 10.1002/aur.3314 (PMC12123175; doi:10.1002/aur.3314)
Supplement: Supplementary file 1 — Figures S1–S8. [file AUR-18-966-s001.pdf]

SUPPLEMENTARY MATERIALS

m^{6}A-mRNA reader *YTHDF2* identified as a potential risk gene in autism with disproportionate megalencephaly

Short running title: Megalencephaly autism risk genes

Sierra S. Nishizaki[1,2,3,4], Nicholas K. Haghani[1,4,5], Gabriana N. La[1,5], Natasha Ann F. Mariano[1,4,5,6], José M. Uribe-Salazar[1,4,5], Gulhan Kaya[1,4,5], Melissa Regester[3,4], Derek Sayre Andrews[2,3,4], Christine Wu Nordahl[2,3,4], David G. Amaral[2,3,4], Megan Y. Dennis[1,2,4,5†]

[1]Genome Center, [2]Autism Research Training Program, [3]Department of Psychiatry and Behavioral Sciences, [4]MIND Institute, [5]Department of Biochemistry & Molecular Medicine, [6]Postbaccalaureate Research Education Program, University of California Davis, CA, USA

[†]Corresponding author:
Megan Y. Dennis, Ph.D.
University of California, Davis
School of Medicine
One Shields Avenue
Genome Center, 4303 GBSF
Davis, CA 95616
Email: mydennis@ucdavis.edu

SUPPLEMENTARY FIGURES

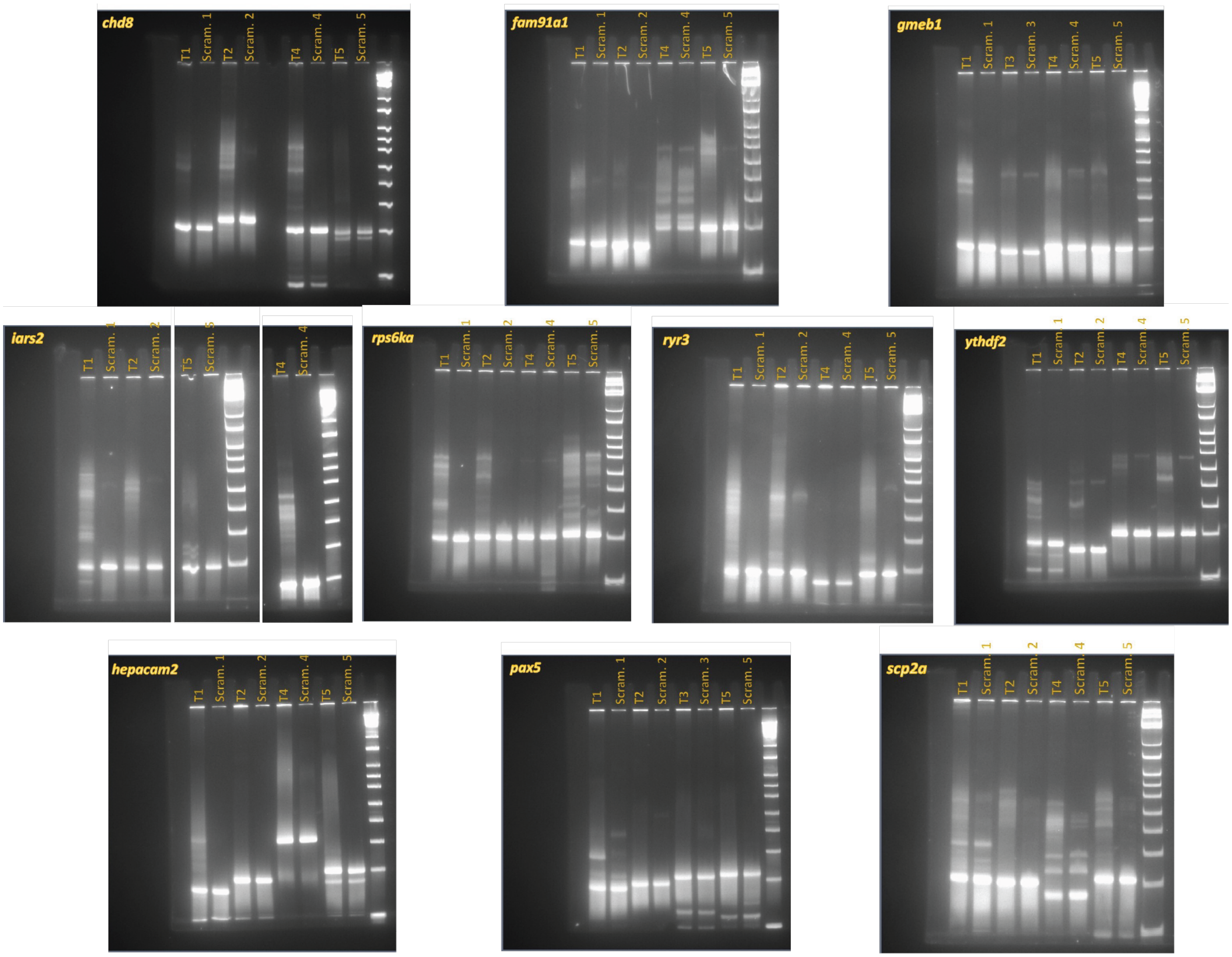

Figure S1. Mutational efficiencies of CRISPR targets in zebrafish embryos. Polyacrylamide gels showing qualitatively the efficiency of each of the four CRISPR gRNAs chosen for the ten genes tested in zebrafish. Targets (T1-5) for each gene refer to the primer pair used to test respective gRNAs listed in Table S1. Respectively, Scram 1-5 serve as controls and a 1kb+ ladder is used for size comparison. Efficiency of gRNAs in F_0 mosaic crispants is observed as a "smear" on the PAGE gel, which indicates formation of heteroduplexes, due to base pair insertions and deletions introduced via CRISPR mutagenesis, during the annealing step of PCR amplification. Further information regarding gRNA efficiency from CrisprVariants scoring methods can be found in Table S1 and Data S1.

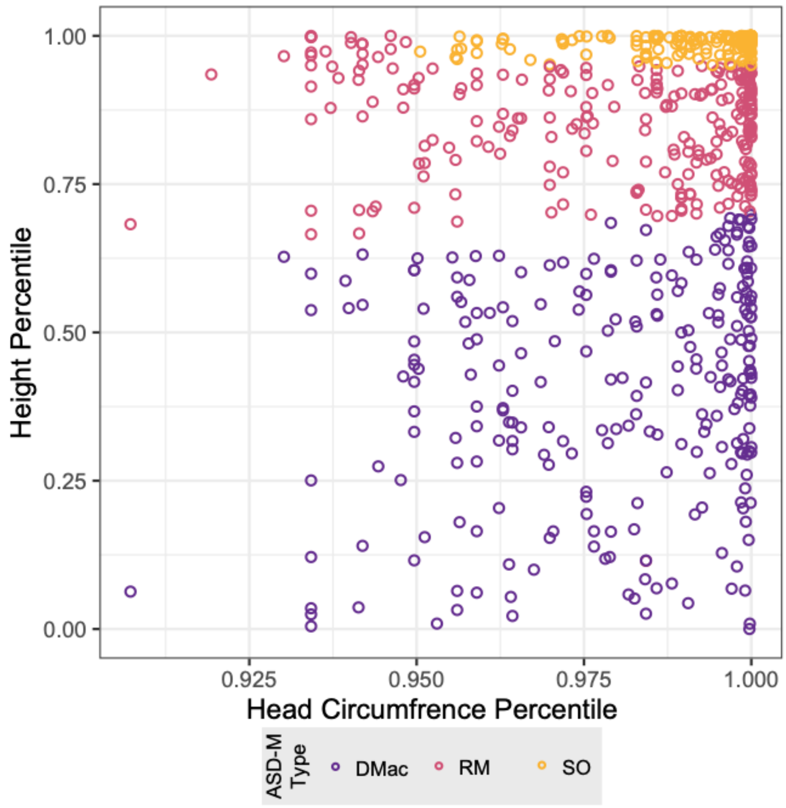

Figure S2. Head circumference v. height percentile of ASD-M SSC probands.

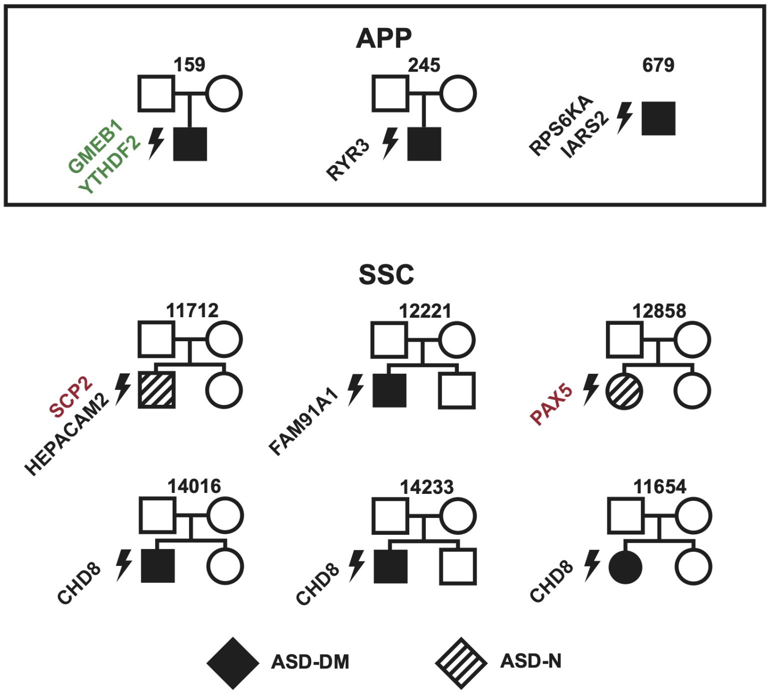

Figure S3. ASD-DM and ASD-M families with *de novo* variants. Families were from the Autism Phenome Project (APP) and the Simons Simplex Cohort (SSC) with impacted genes that were screened using zebrafish in this study. Green font signifies a duplication and red font signifies a deletion.

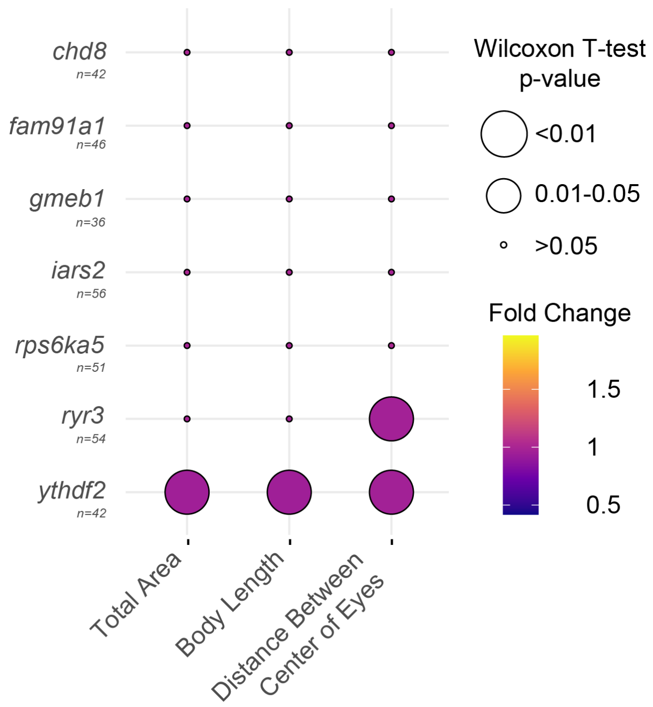

Figure S4. **Zebrafish CRISPR knockdown embryos for the rapid validation of ASD-DM genes.** Phenotyping knockdown zebrafish morphometric measurements using the VAST BioImaging System identified *ythdf2* as having most obvious morphometric differences compared to a negative scrambled control.

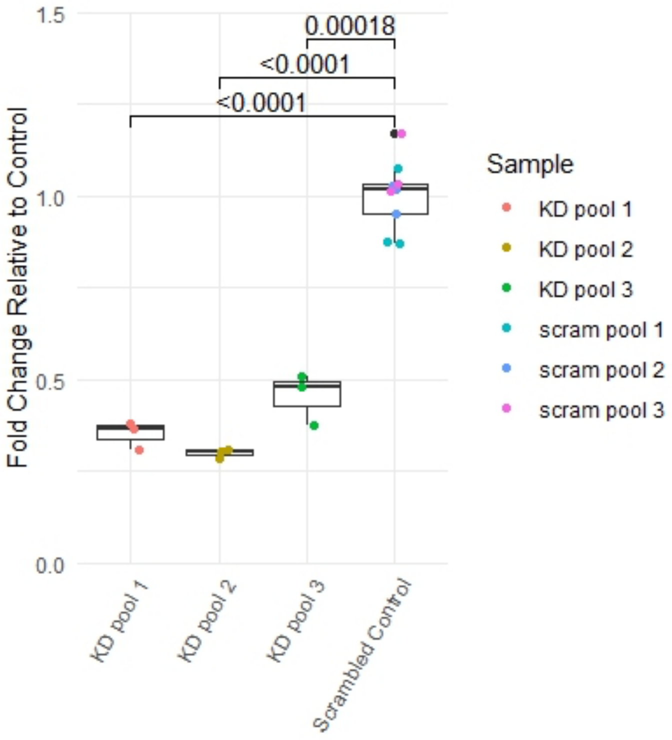

Figure S5. Gene expression analysis of *ythdf2* in crispant mutants. RT-qPCR results for across *ythdf2* knockdown (KD) and scrambled control biological replicates. All samples demonstrate consistent *act1b* expression, while the pooled *ythdf2* KD samples demonstrate lower *ythdf2* expression than the scrambled control. *p*-values were calculated using a Student's T-test.

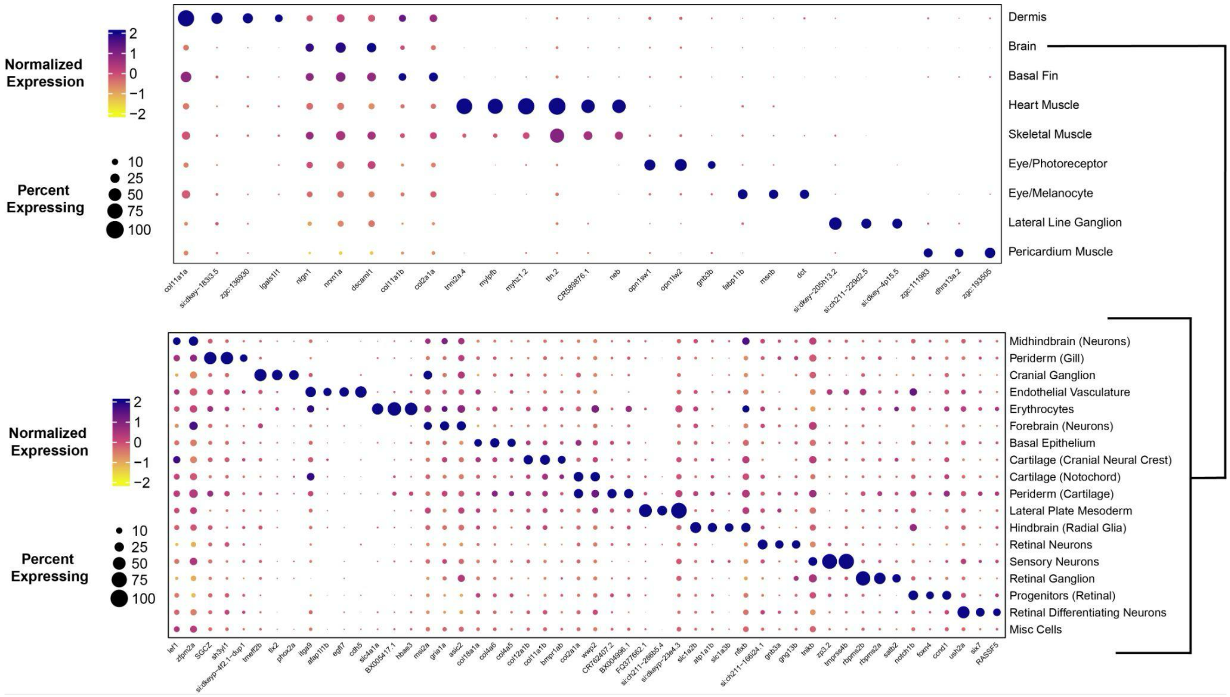

Figure S6. Single-cell transcriptomic markers. Top three gene markers by cell cluster utilizing *FindAllMarkers* and *Extract_Top_Markers* from *Seurat* and *scCustomize* expressed in at least 20 percent of marked cluster for all cells (top) and brain cells (bottom).

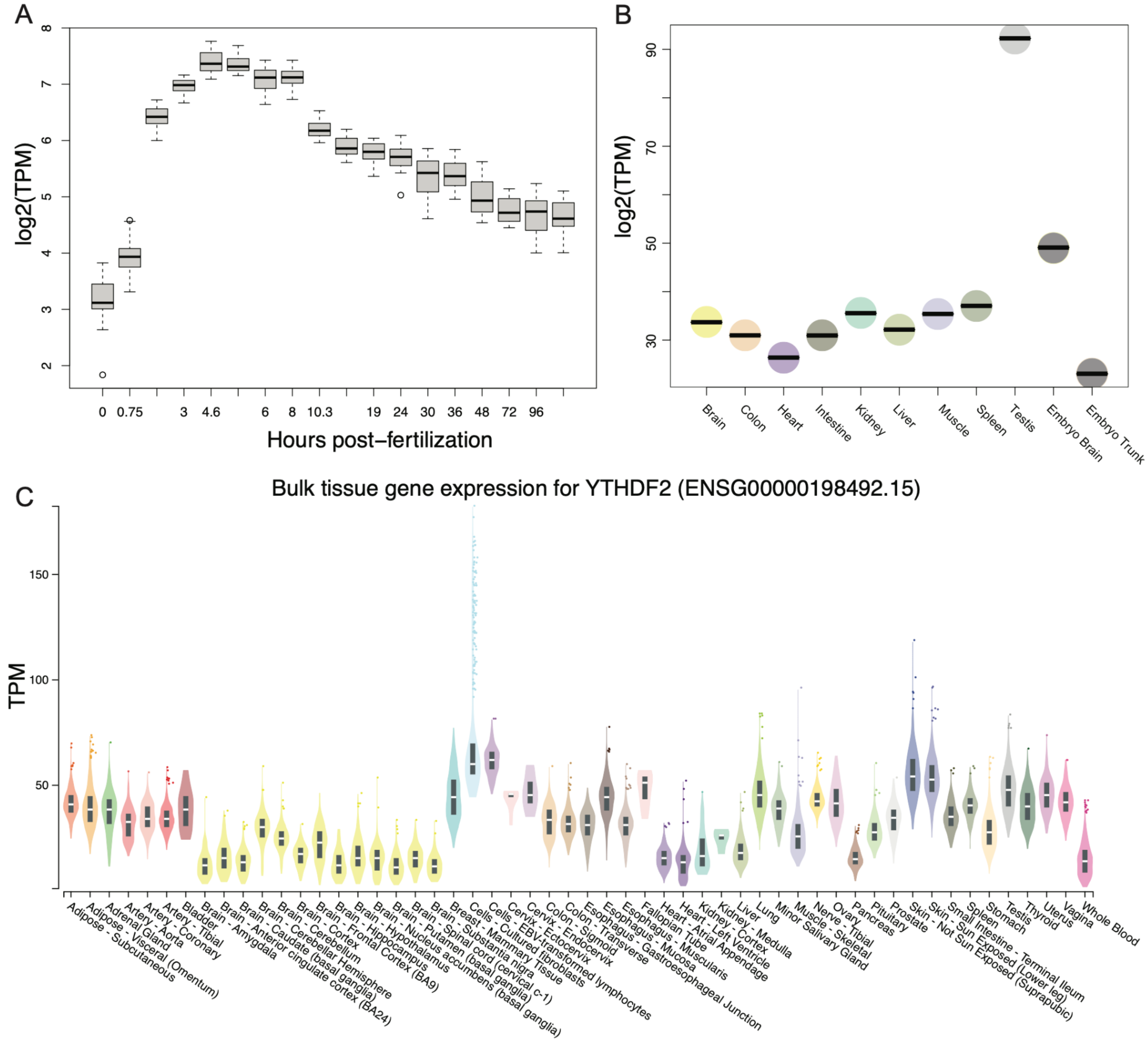

Figure S7. Expression of *YTHDF2* in zebrafish and humans. (**A**) Expression of *ythdf2* showing increased transcript per million (TPM) over early developmental time, peaking at ~5 hours post fertilization [1]. (**B**) Bulk tissue expression of *ythdf2* in zebrafish showing broad expression, highest in adult testis and embryo brain [2]. (**C**) Bulk tissue gene expression of *YTHDF2* from the Genotype-Tissue Expression project (GTEx) showing high broad expression in humans [3].

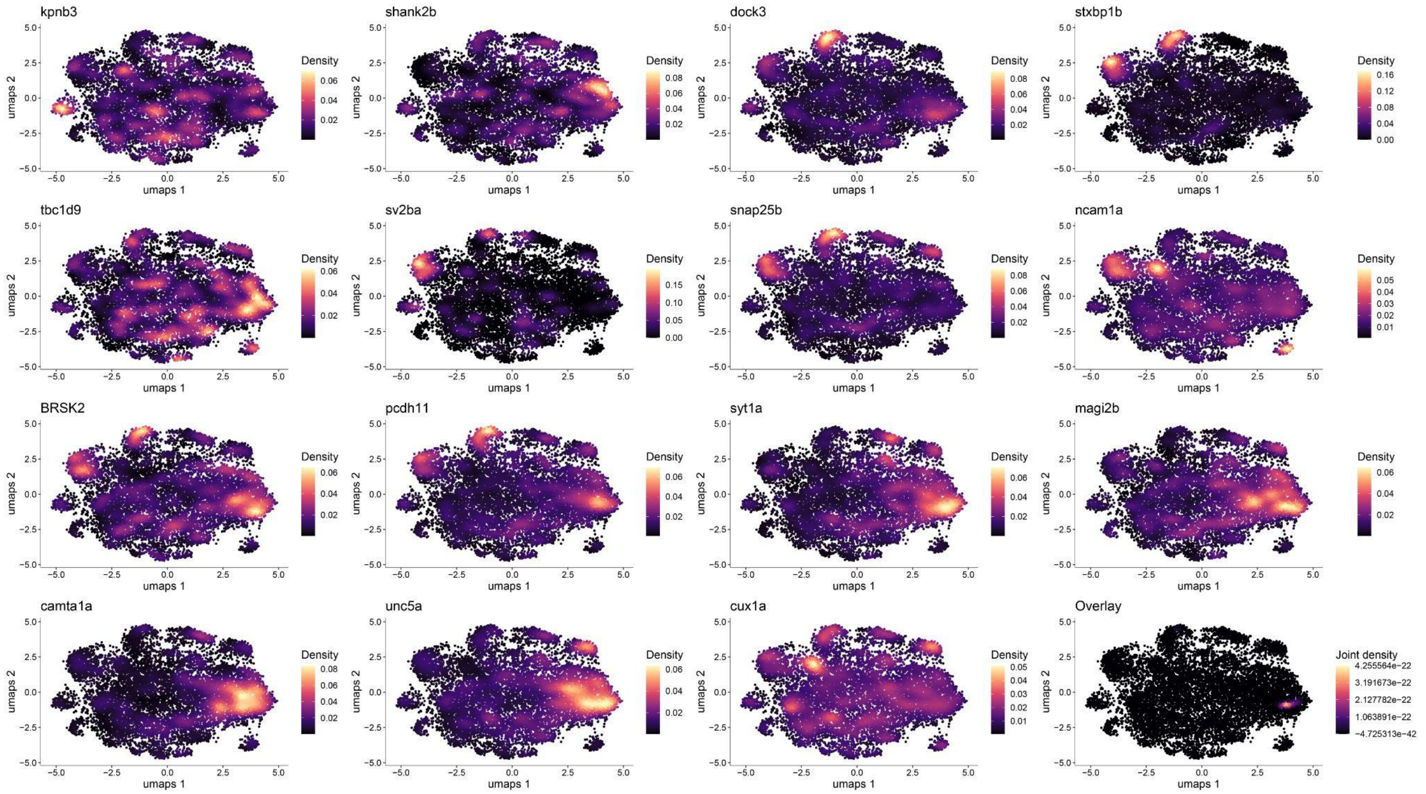

Figure S8. FMRP targets gene expression signals. Expression profiles across brain cell types of 15 significantly differentially expressed FMRP target genes, and the overlay of all genes (bottom right), utilizing weighted kernel density estimation. Plots were created using *plot_density* from the *Nebulosa* package.

SUPPLEMENTARY REFERENCES

1. White RJ, Collins JE, Sealy IM, Wali N, Dooley CM, Digby Z, et al. A high-resolution mRNA expression time course of embryonic development in zebrafish. doi:10.1101/107631

2. Yang H, Luan Y, Liu T, Lee HJ, Fang L, Wang Y, et al. A map of cis-regulatory elements and 3D genome structures in zebrafish. Nature. 2020;588: 337–343.

3. GTEx Consortium. The GTEx Consortium atlas of genetic regulatory effects across human tissues. Science. 2020;369: 1318–1330.
