# Supplementary figures and images for: m6A‐mRNA Reader YTHDF2 Identified as a Potential Risk Gene in Autism With Disproportionate Megalencephaly
Source: Autism Res. 2025 Jan 30;18(5):966–82. doi: 10.1002/aur.3314 (PMC12123175; doi:10.1002/aur.3314)

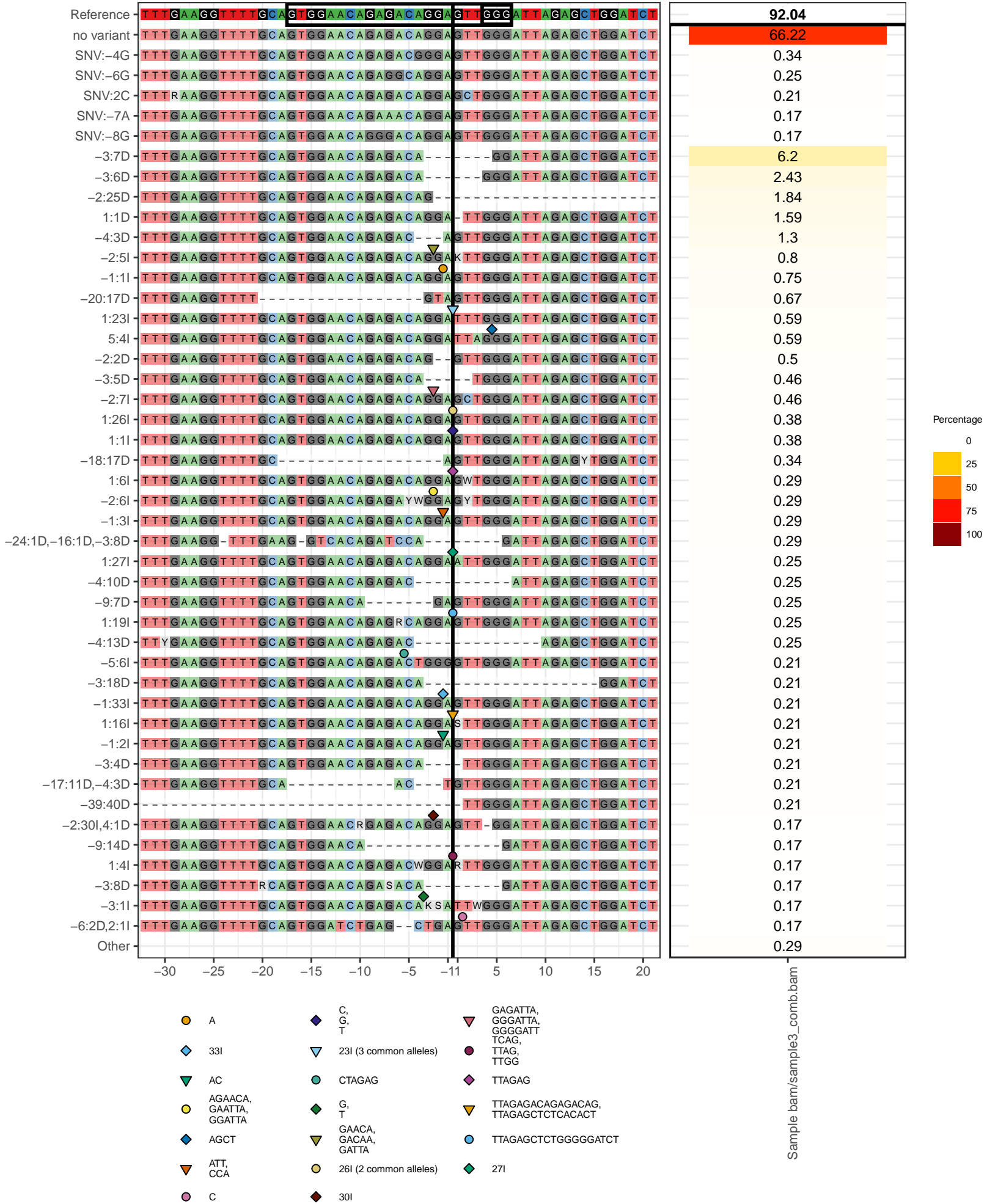

Supplement: Supplementary file 2 — Data S1. [file AUR-18-966-s002.zip › chd8.4.pdf]

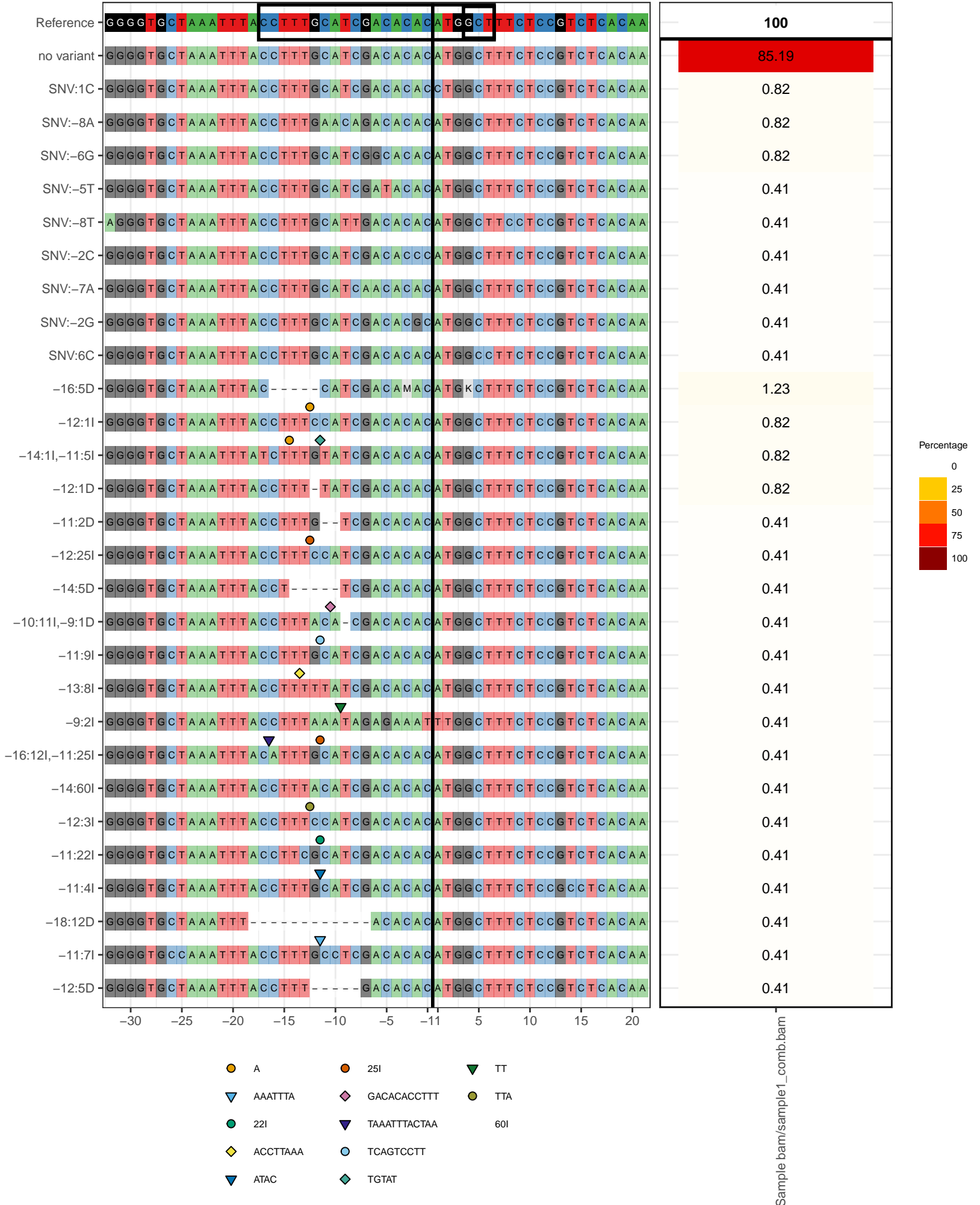

Supplement: Supplementary file 2 — Data S1. [file AUR-18-966-s002.zip › chd8.1.pdf]

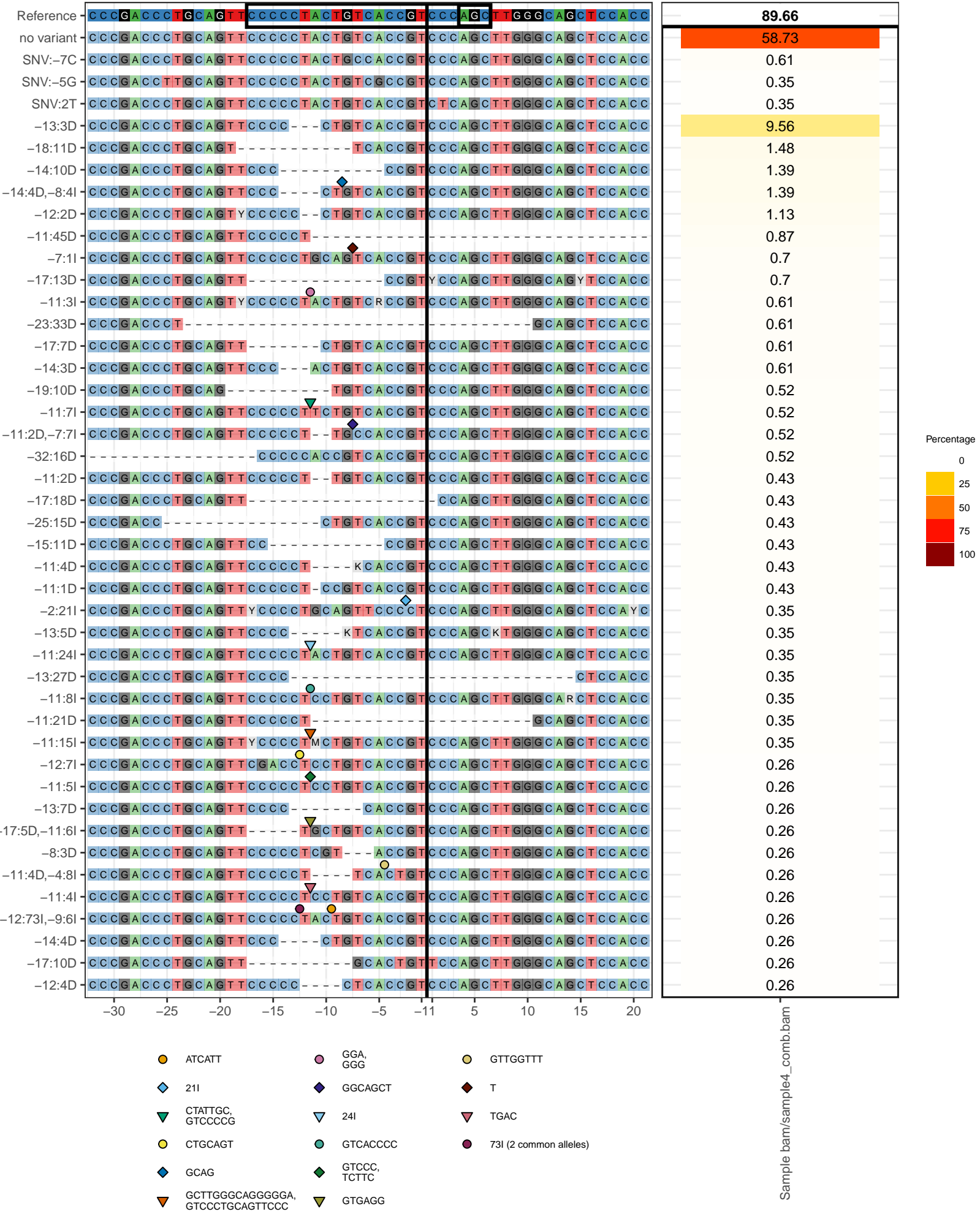

Supplement: Supplementary file 2 — Data S1. [file AUR-18-966-s002.zip › chd8.5.pdf]

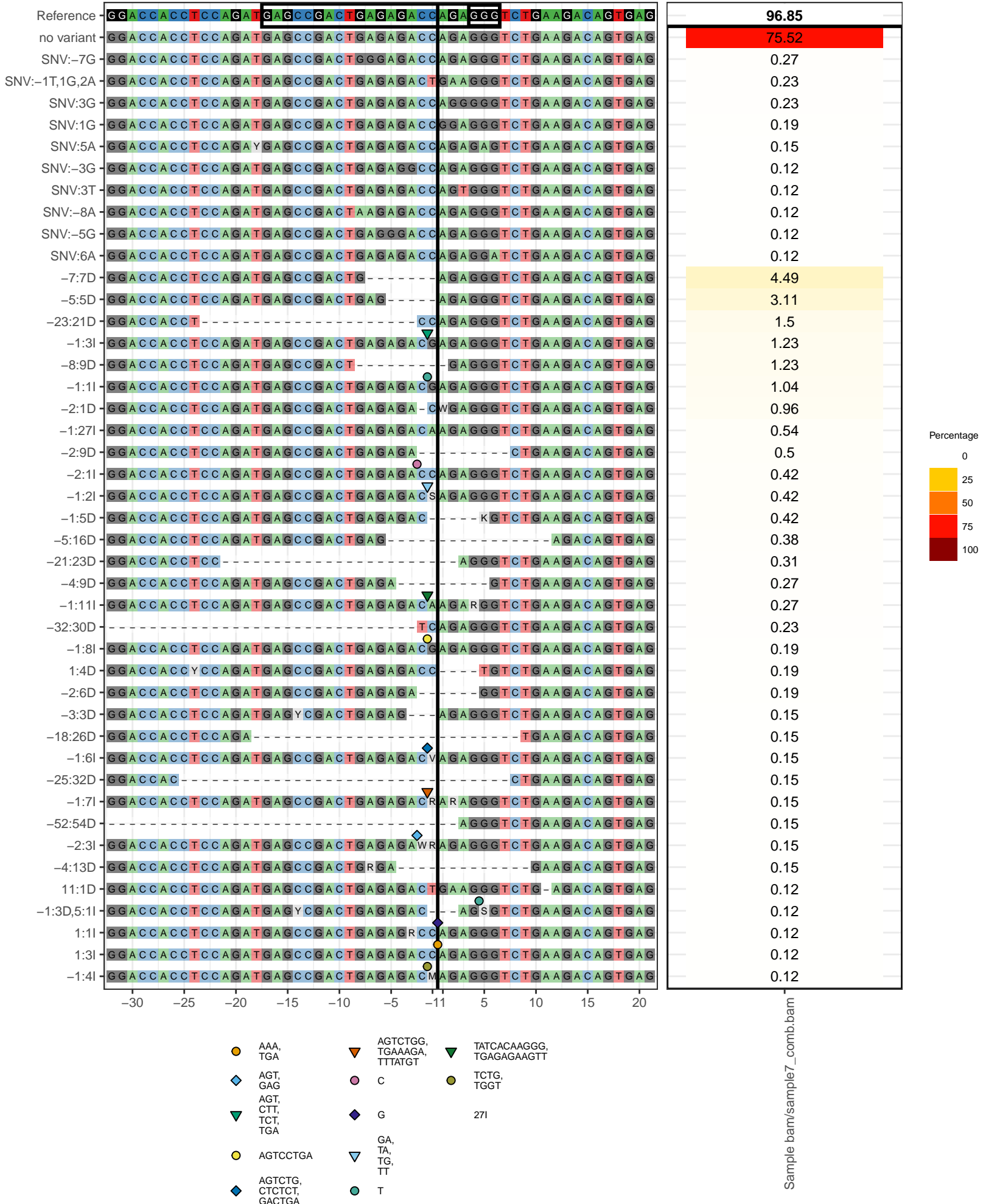

Supplement: Supplementary file 2 — Data S1. [file AUR-18-966-s002.zip › cnksr2a.5.pdf]

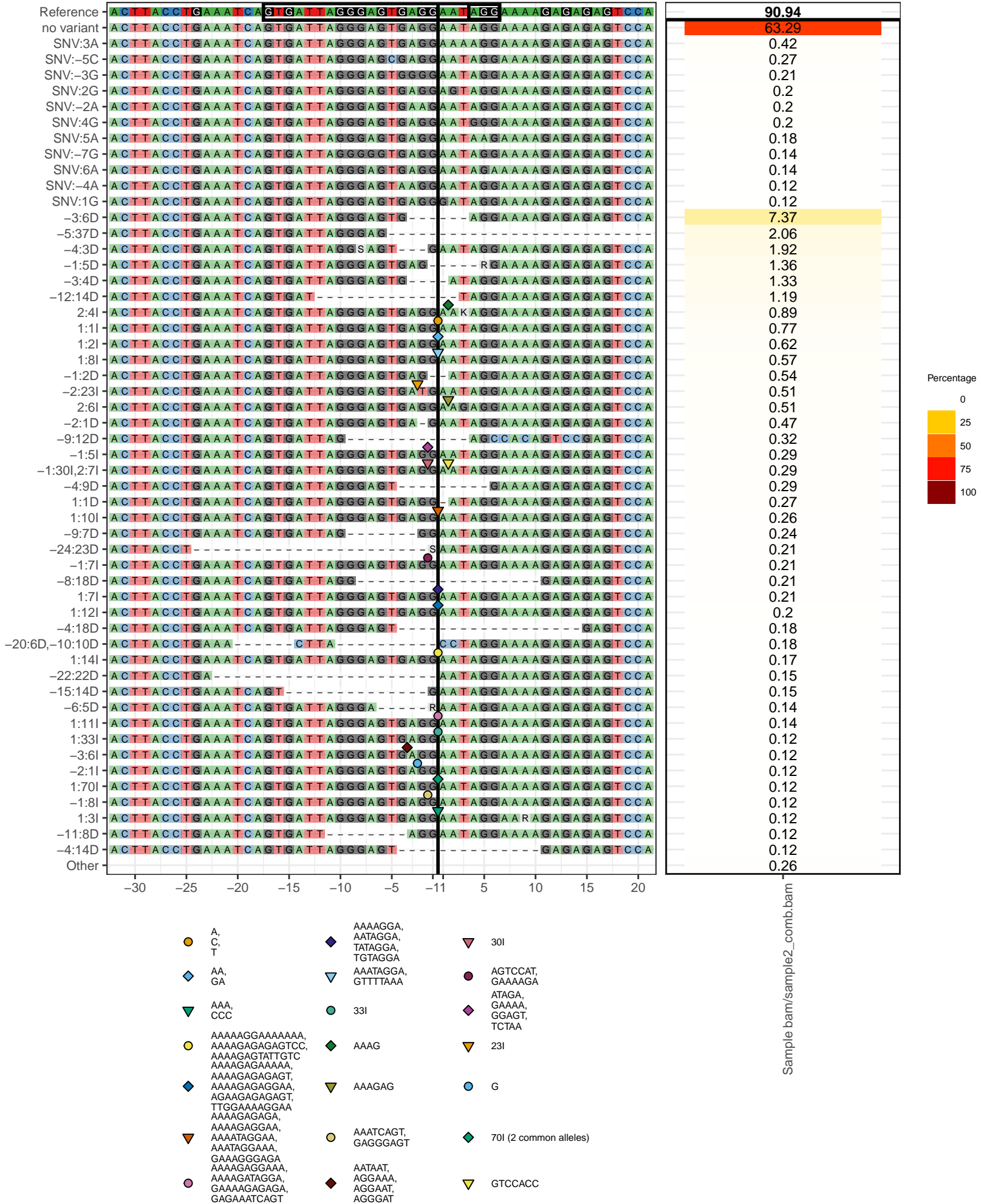

Supplement: Supplementary file 2 — Data S1. [file AUR-18-966-s002.zip › chd8.2.pdf]

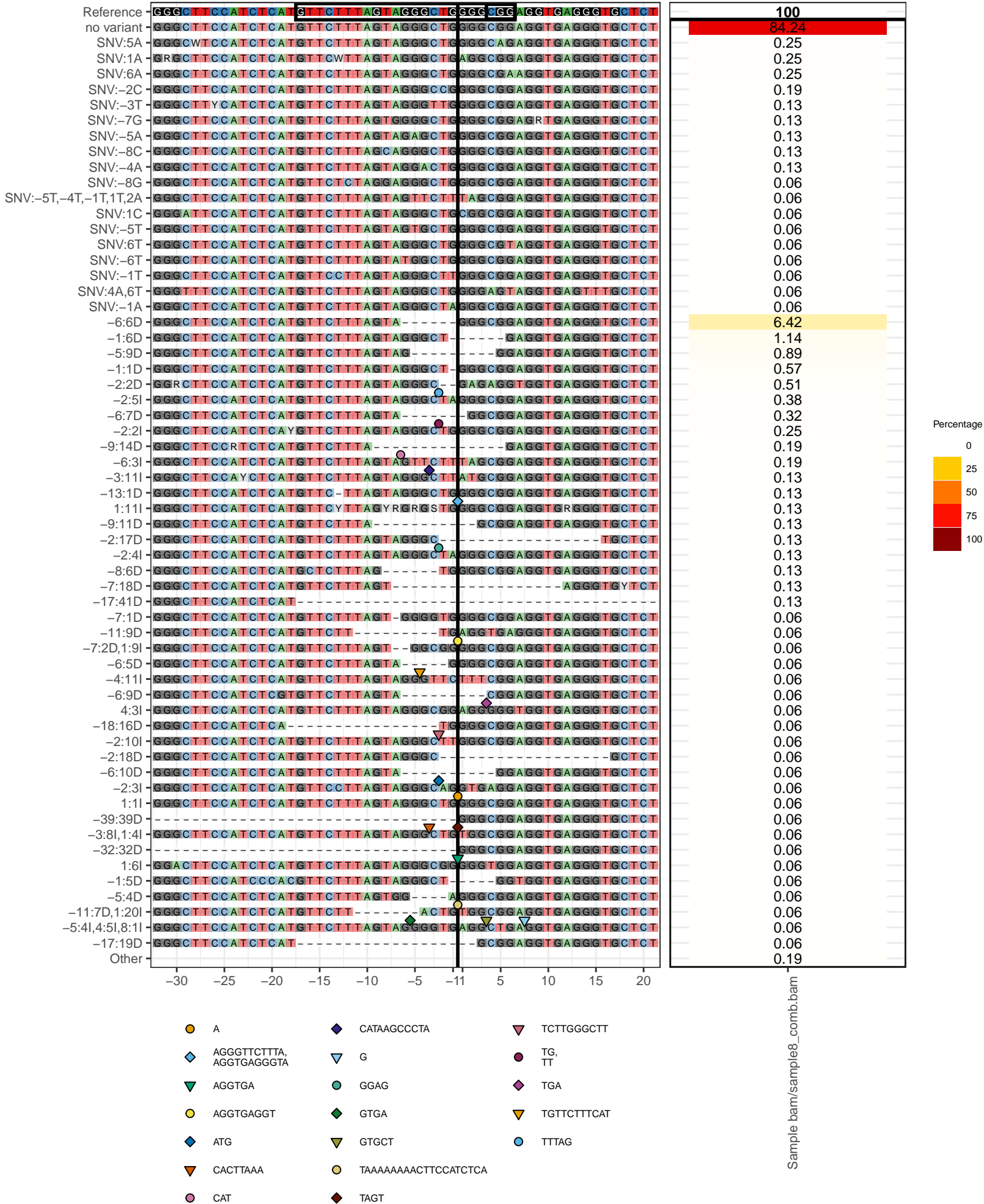

Supplement: Supplementary file 2 — Data S1. [file AUR-18-966-s002.zip › cnksr2a.6.pdf]

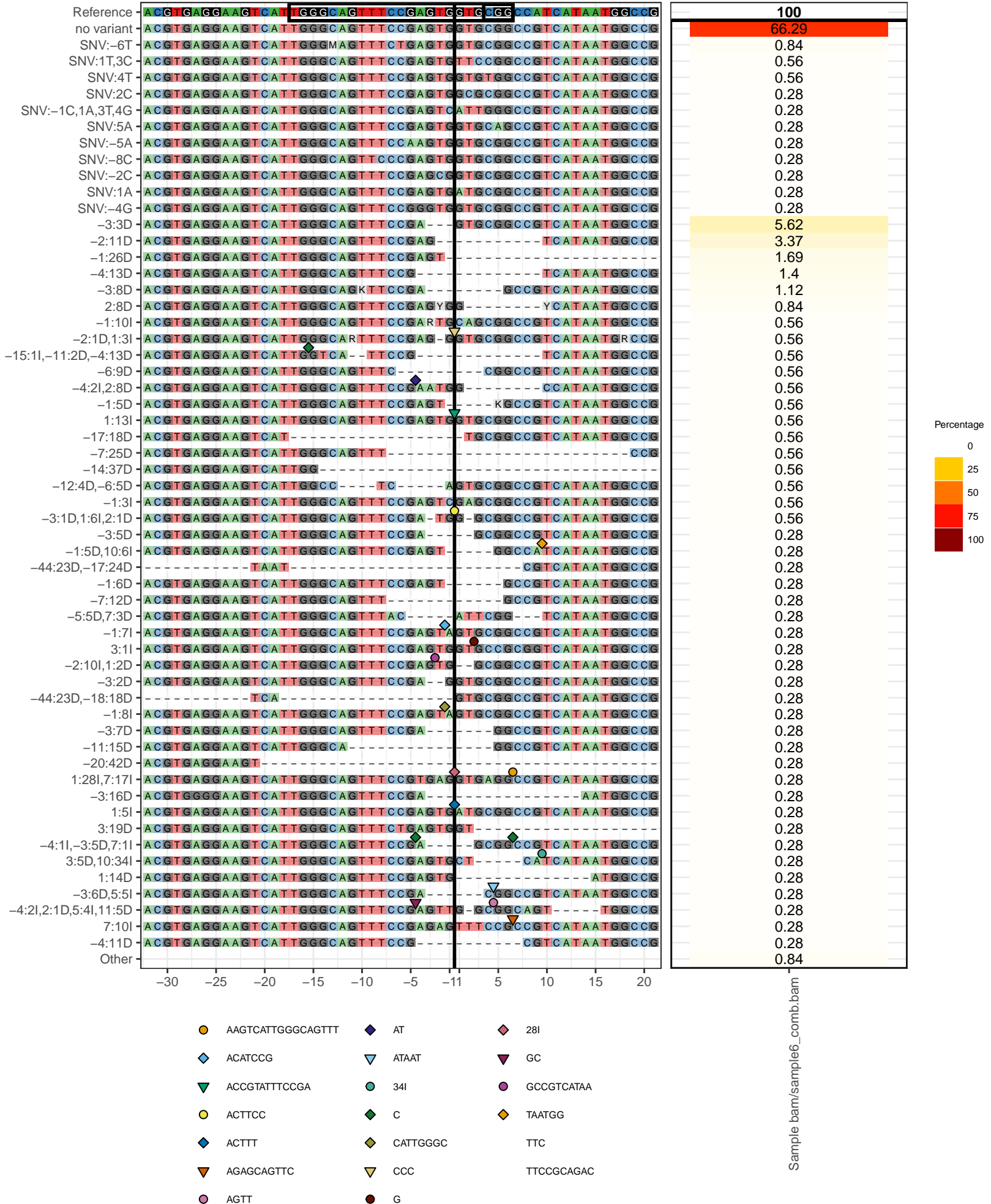

Supplement: Supplementary file 2 — Data S1. [file AUR-18-966-s002.zip › cnksr2a.2.pdf]

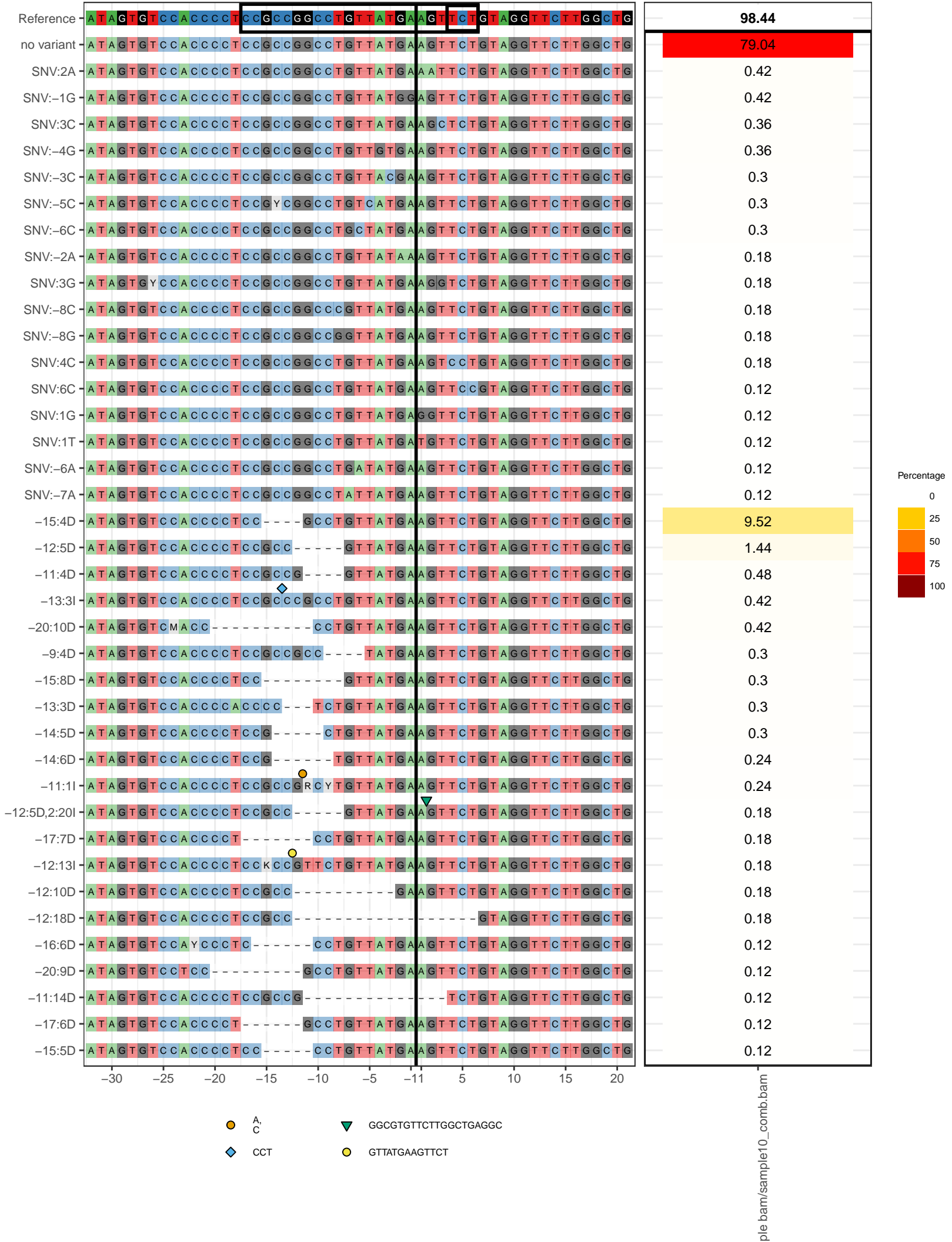

Supplement: Supplementary file 2 — Data S1. [file AUR-18-966-s002.zip › cnksr2b.2.pdf]

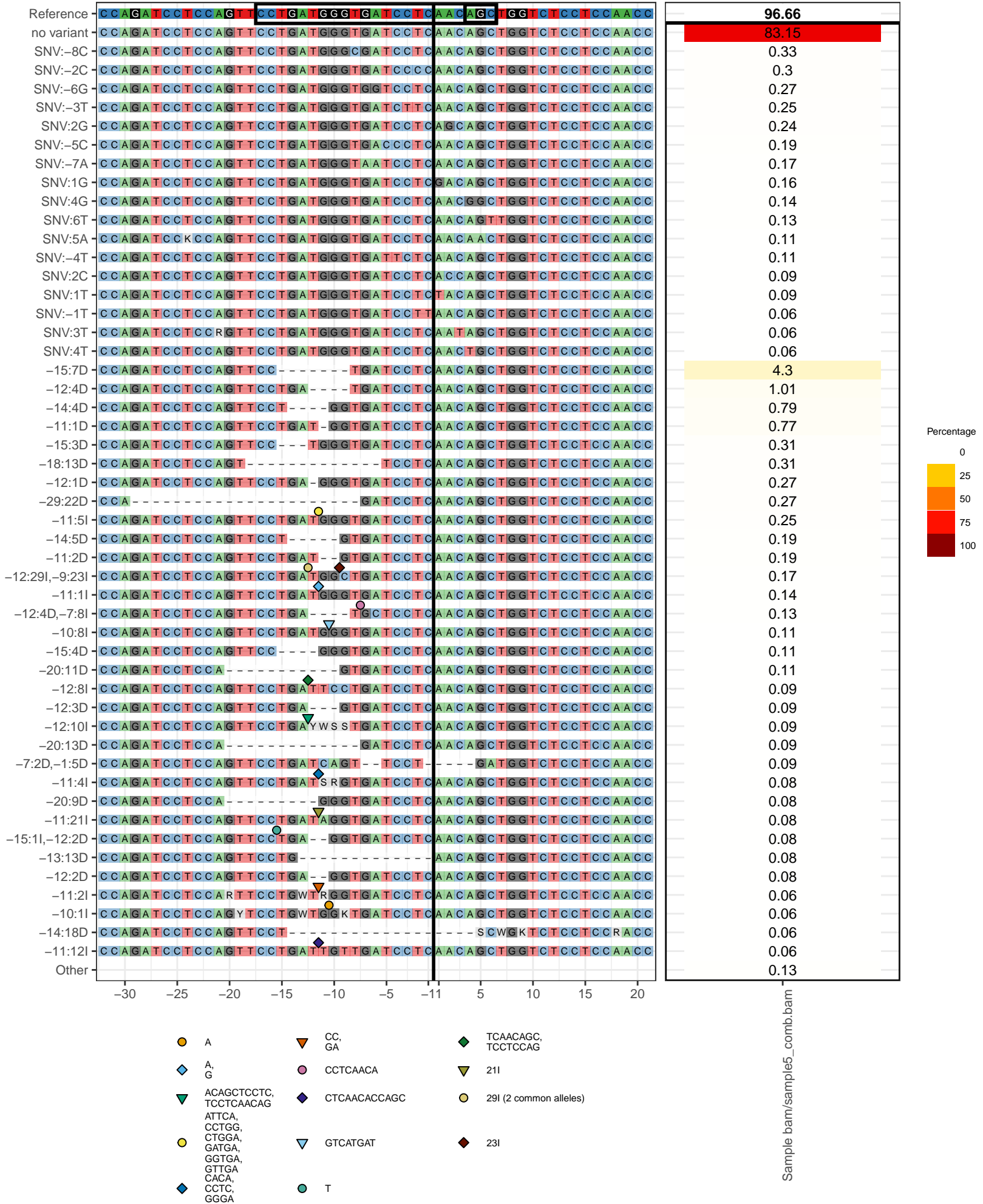

Supplement: Supplementary file 2 — Data S1. [file AUR-18-966-s002.zip › cnksr2a.1.pdf]

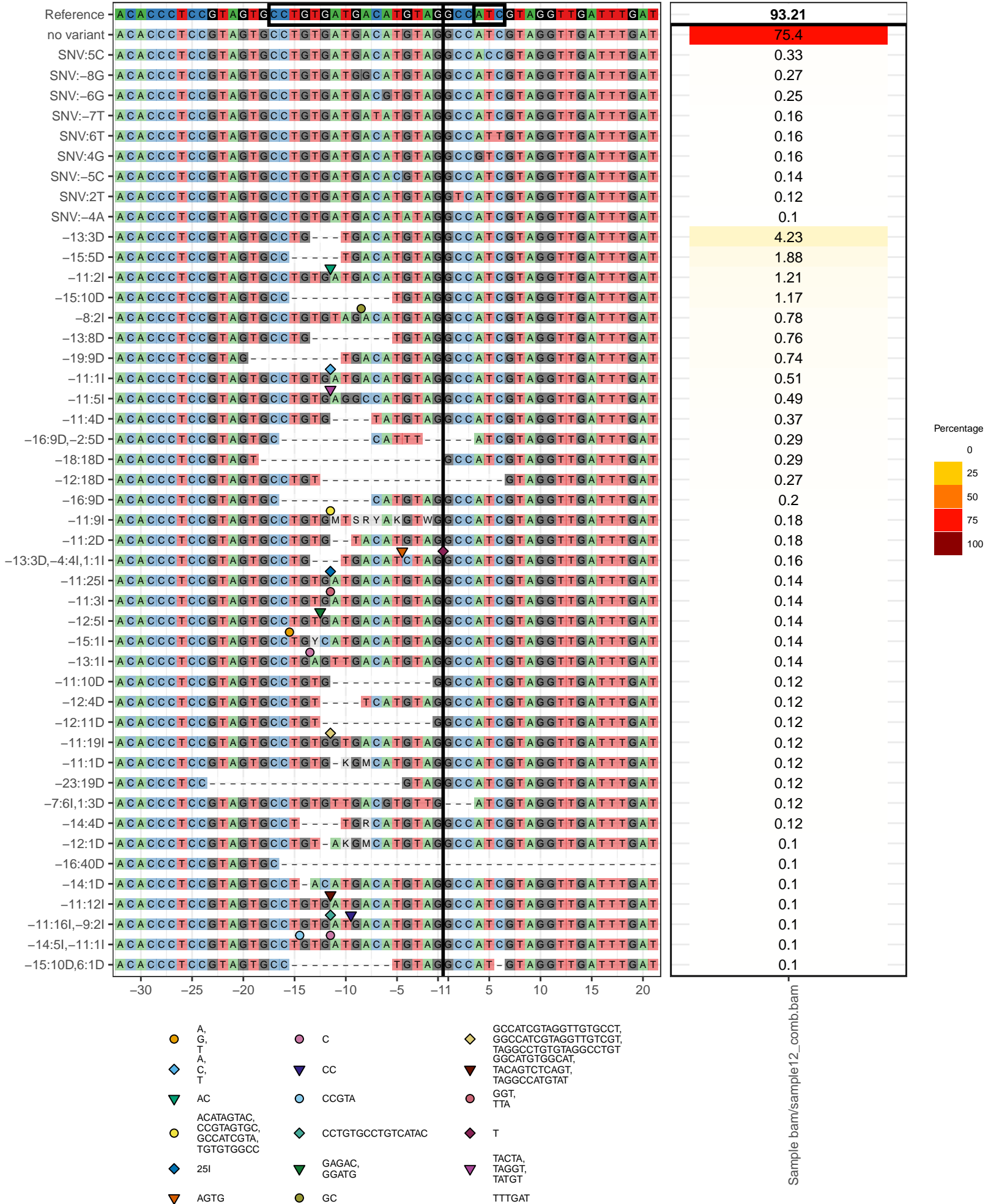

Supplement: Supplementary file 2 — Data S1. [file AUR-18-966-s002.zip › cnksr2b.6.pdf]

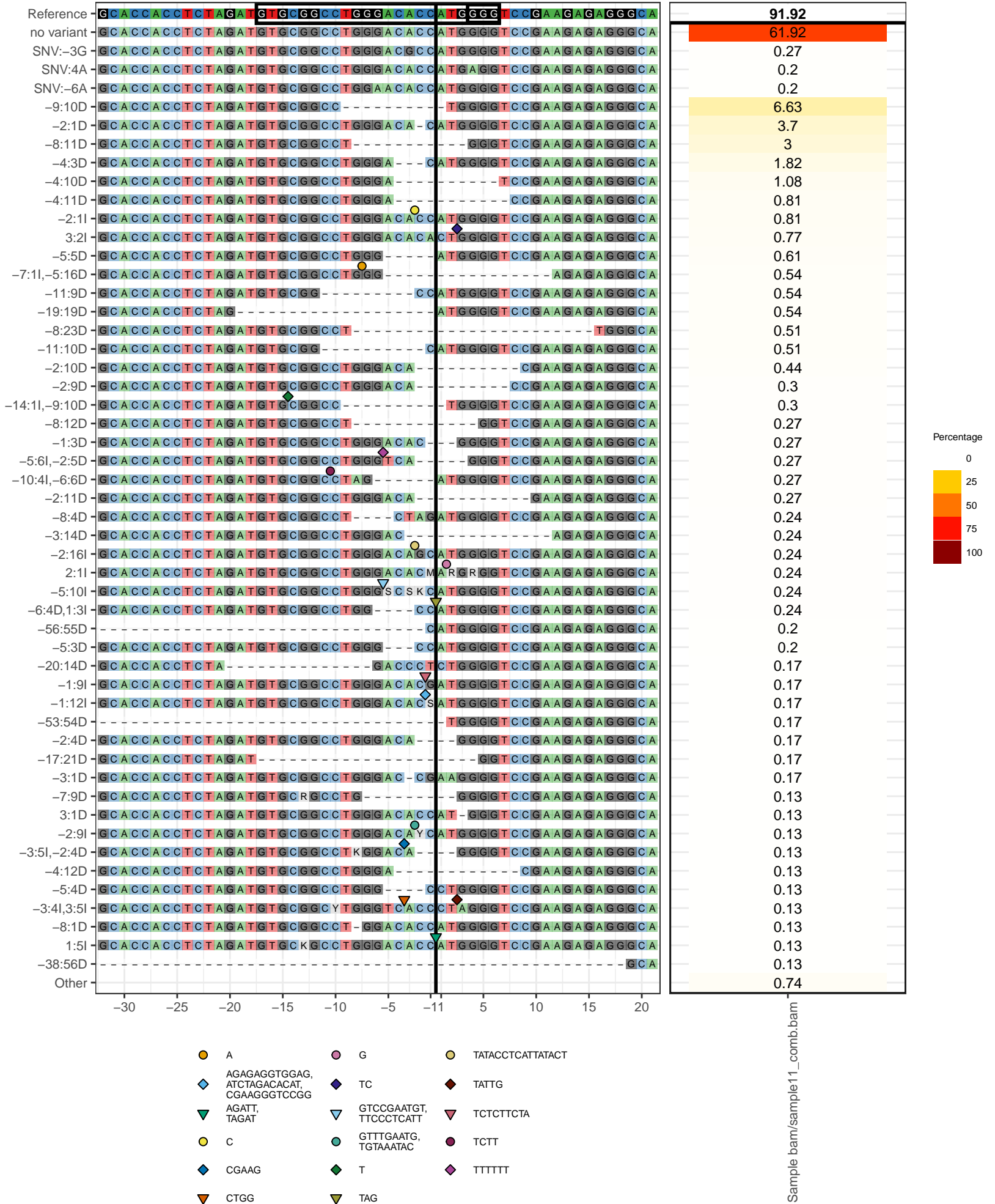

Supplement: Supplementary file 2 — Data S1. [file AUR-18-966-s002.zip › cnksr2b.5.pdf]

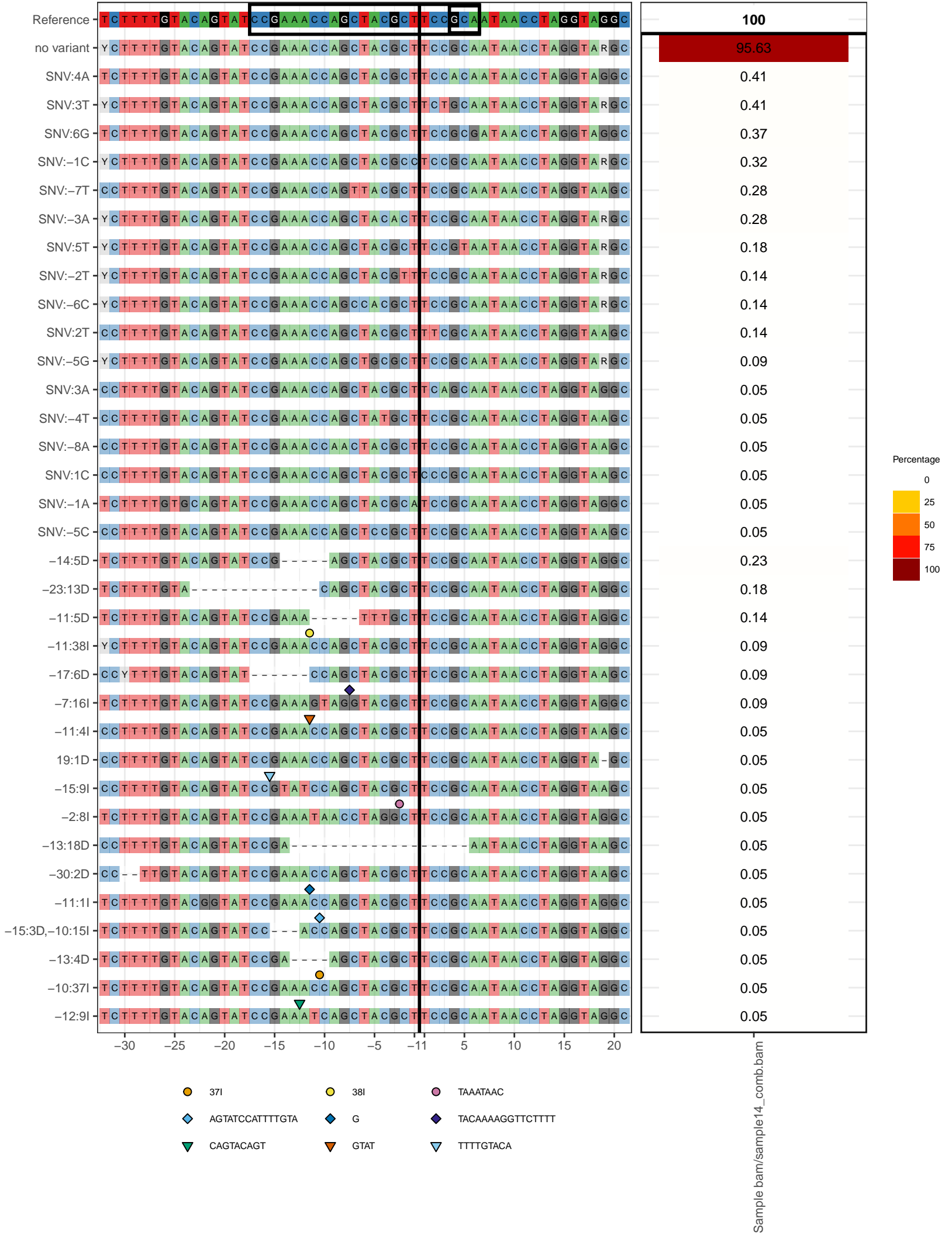

Supplement: Supplementary file 2 — Data S1. [file AUR-18-966-s002.zip › fam91a1.2.pdf]

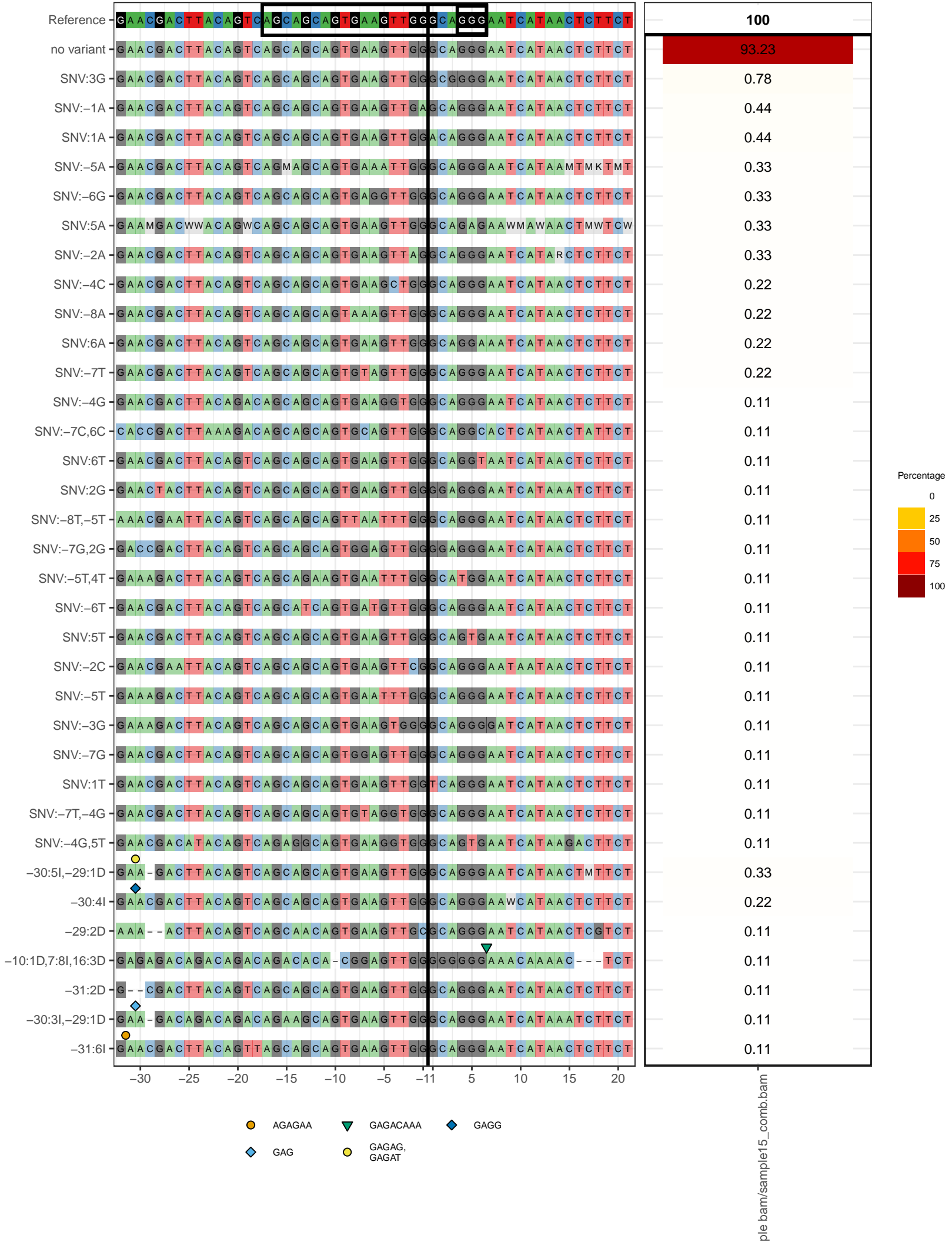

Supplement: Supplementary file 2 — Data S1. [file AUR-18-966-s002.zip › fam91a1.4.pdf]

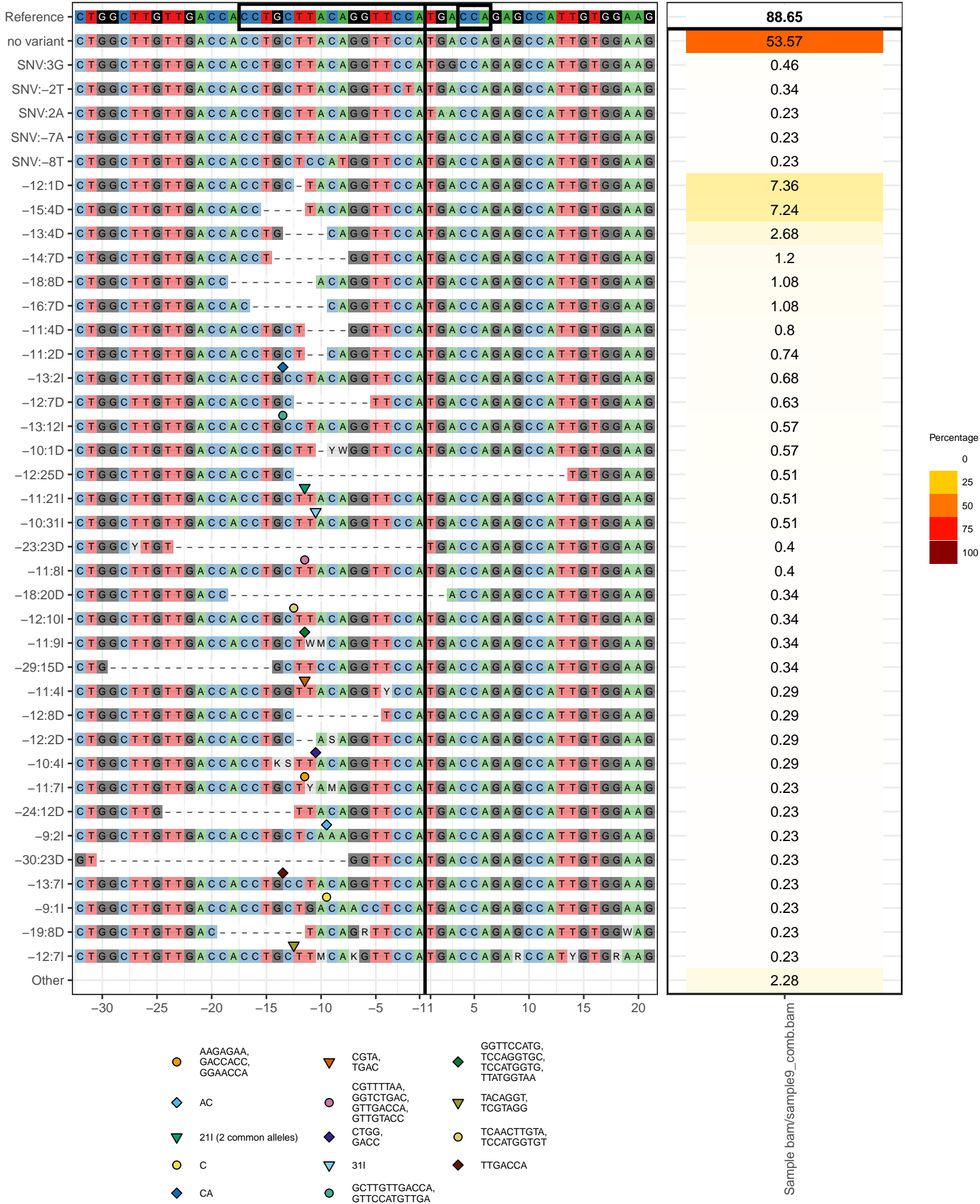

Supplement: Supplementary file 2 — Data S1. [file AUR-18-966-s002.zip › cnksr2b.1.pdf]

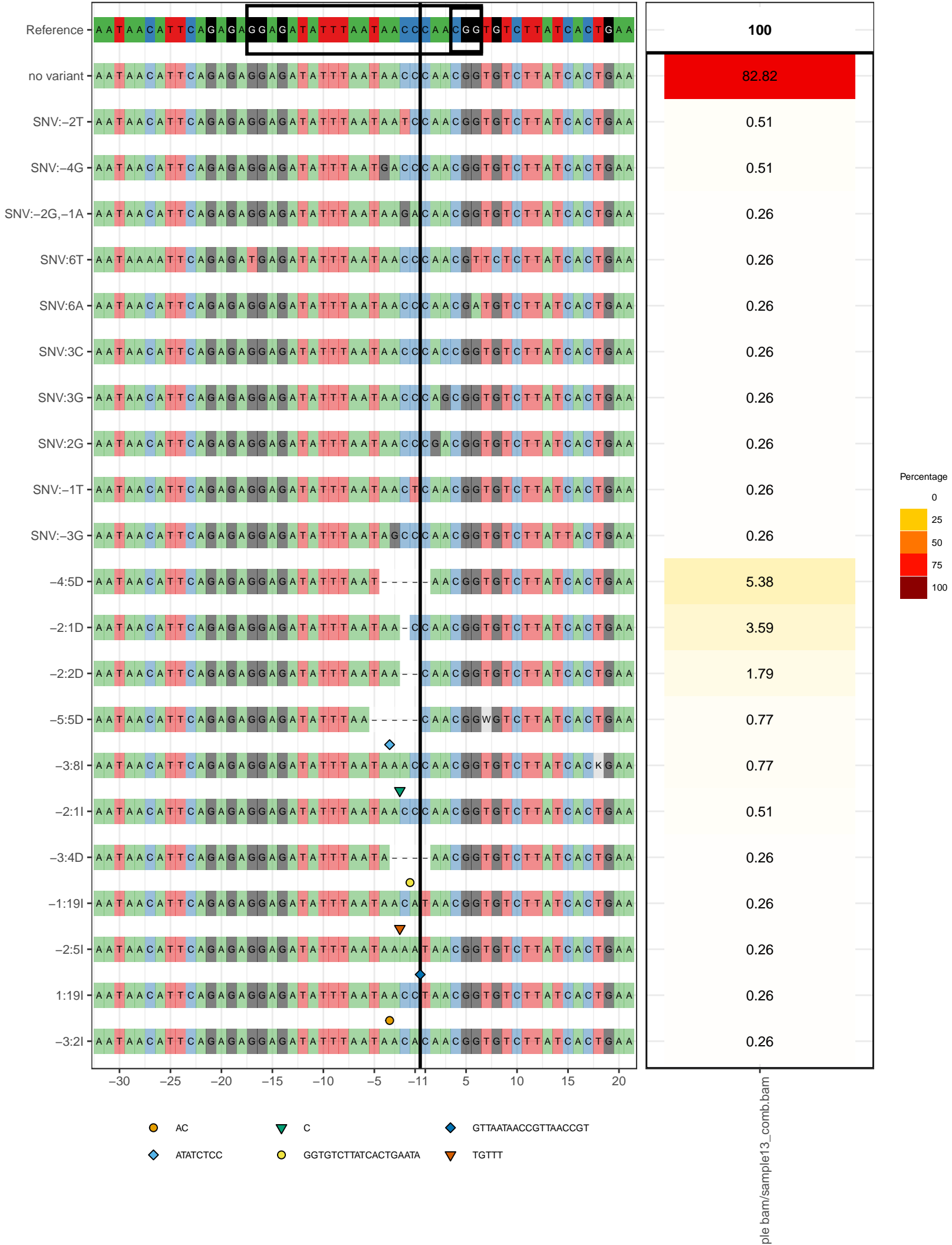

Supplement: Supplementary file 2 — Data S1. [file AUR-18-966-s002.zip › fam91a1.1.pdf]

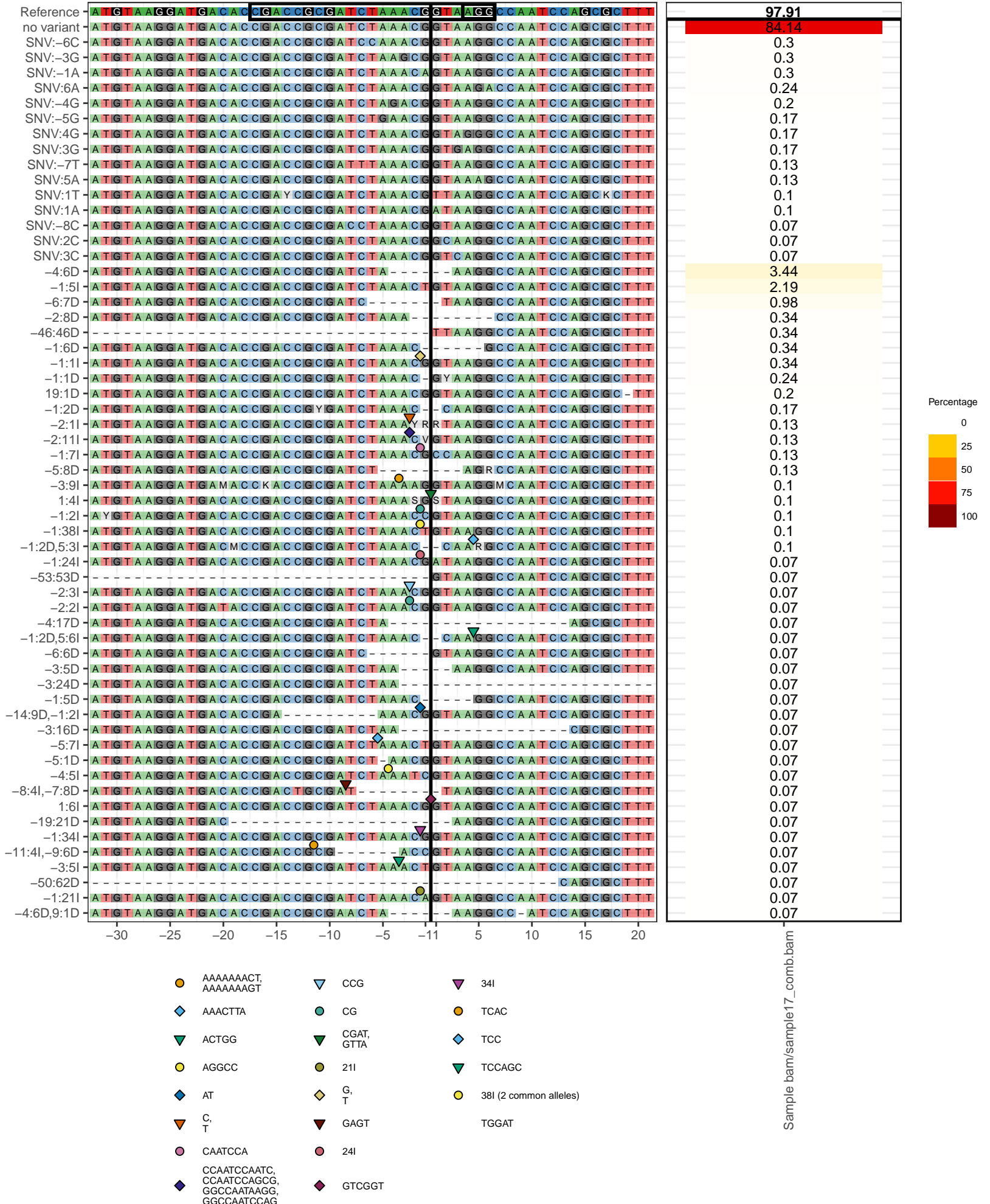

Supplement: Supplementary file 2 — Data S1. [file AUR-18-966-s002.zip › gmeb1.1.pdf]

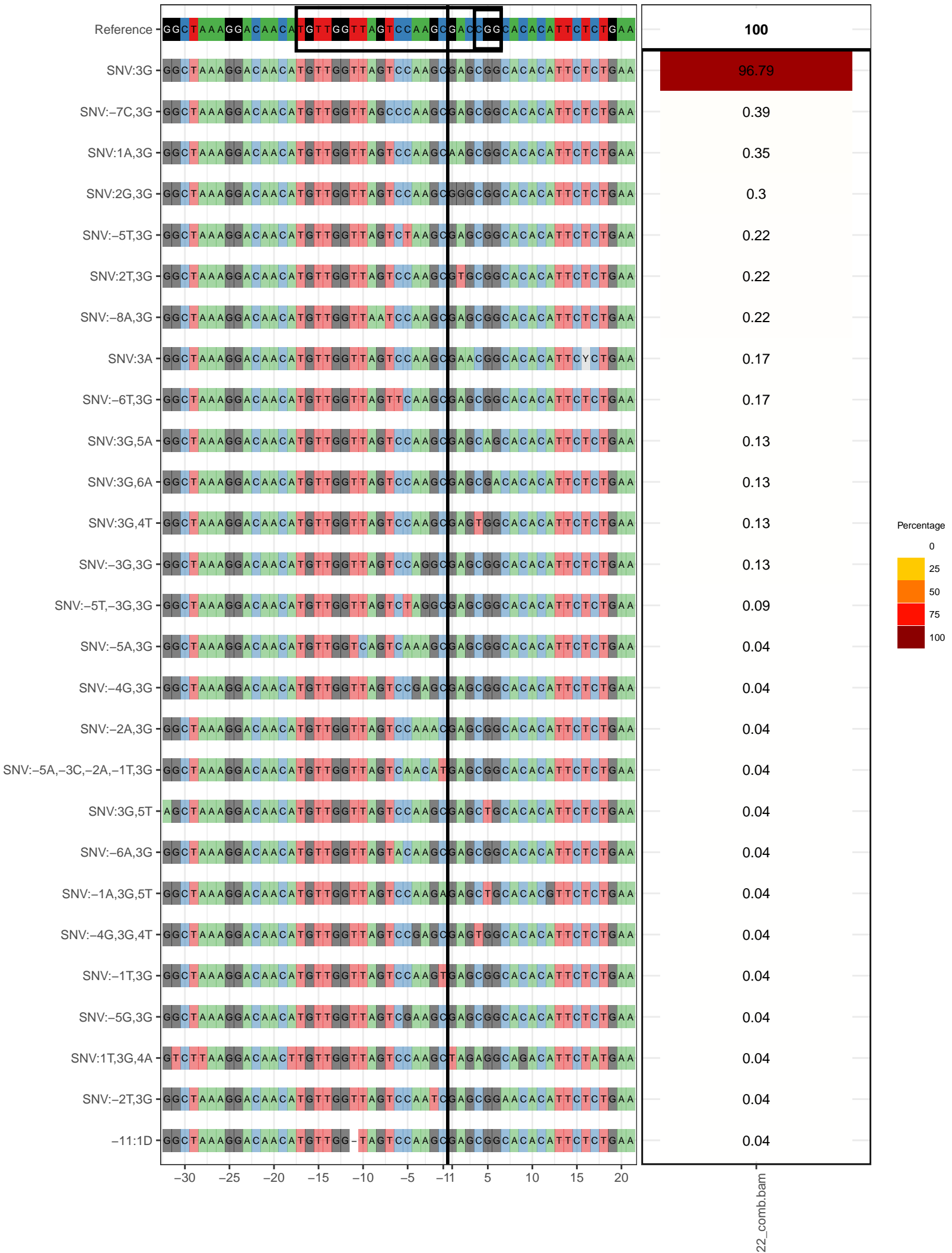

Supplement: Supplementary file 2 — Data S1. [file AUR-18-966-s002.zip › hepacam2.2.pdf]

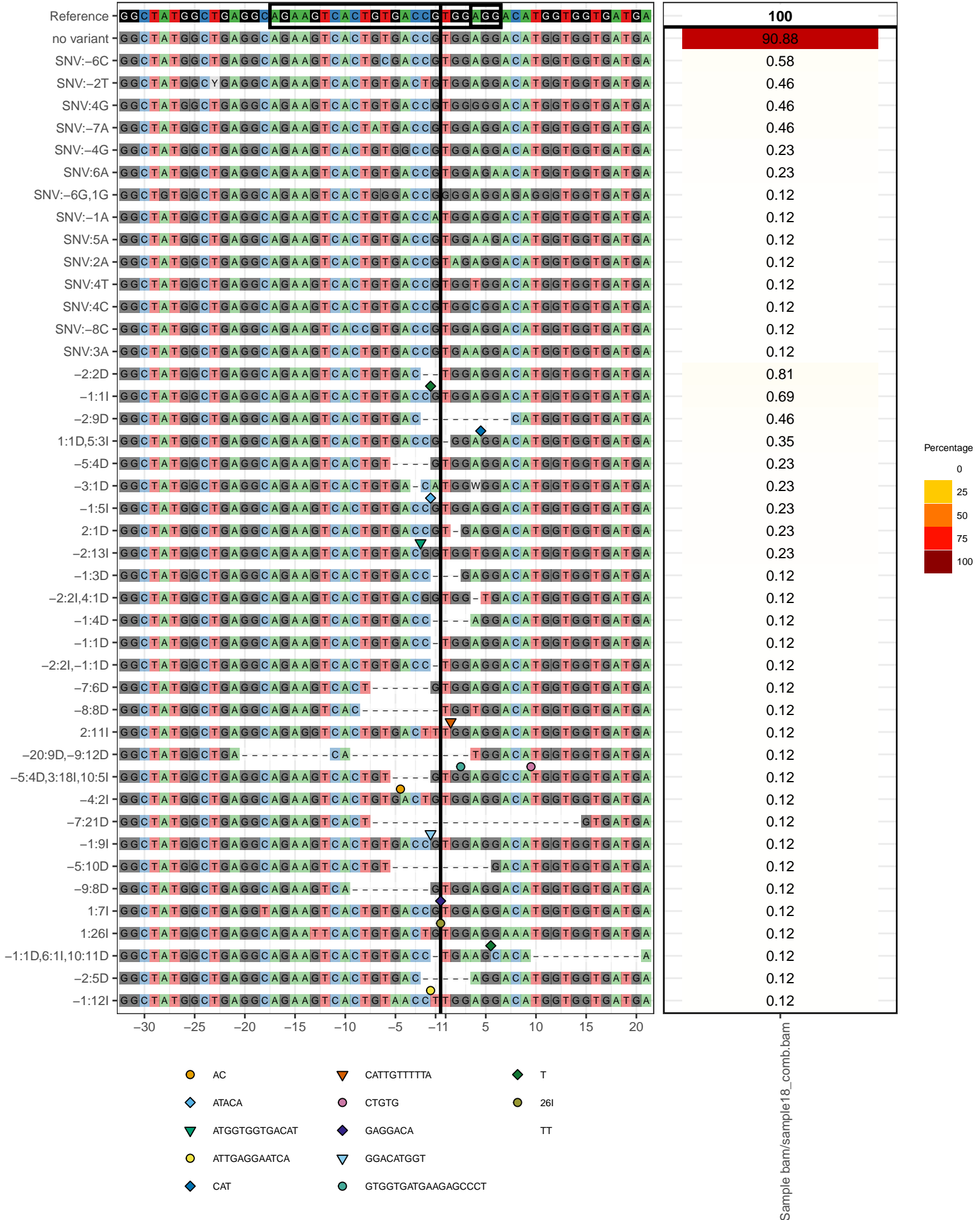

Supplement: Supplementary file 2 — Data S1. [file AUR-18-966-s002.zip › gmeb1.3.pdf]

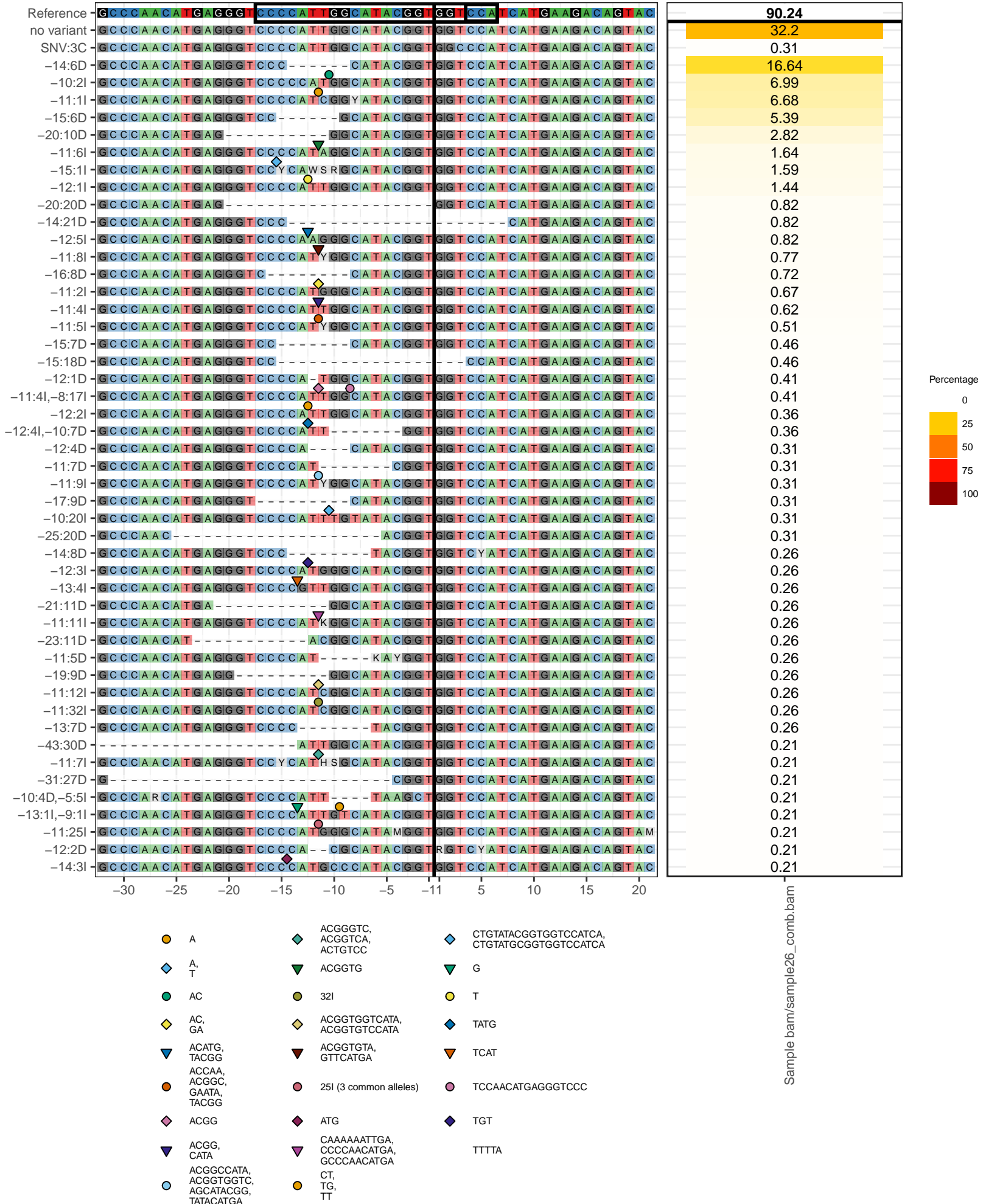

Supplement: Supplementary file 2 — Data S1. [file AUR-18-966-s002.zip › iars2.2.pdf]

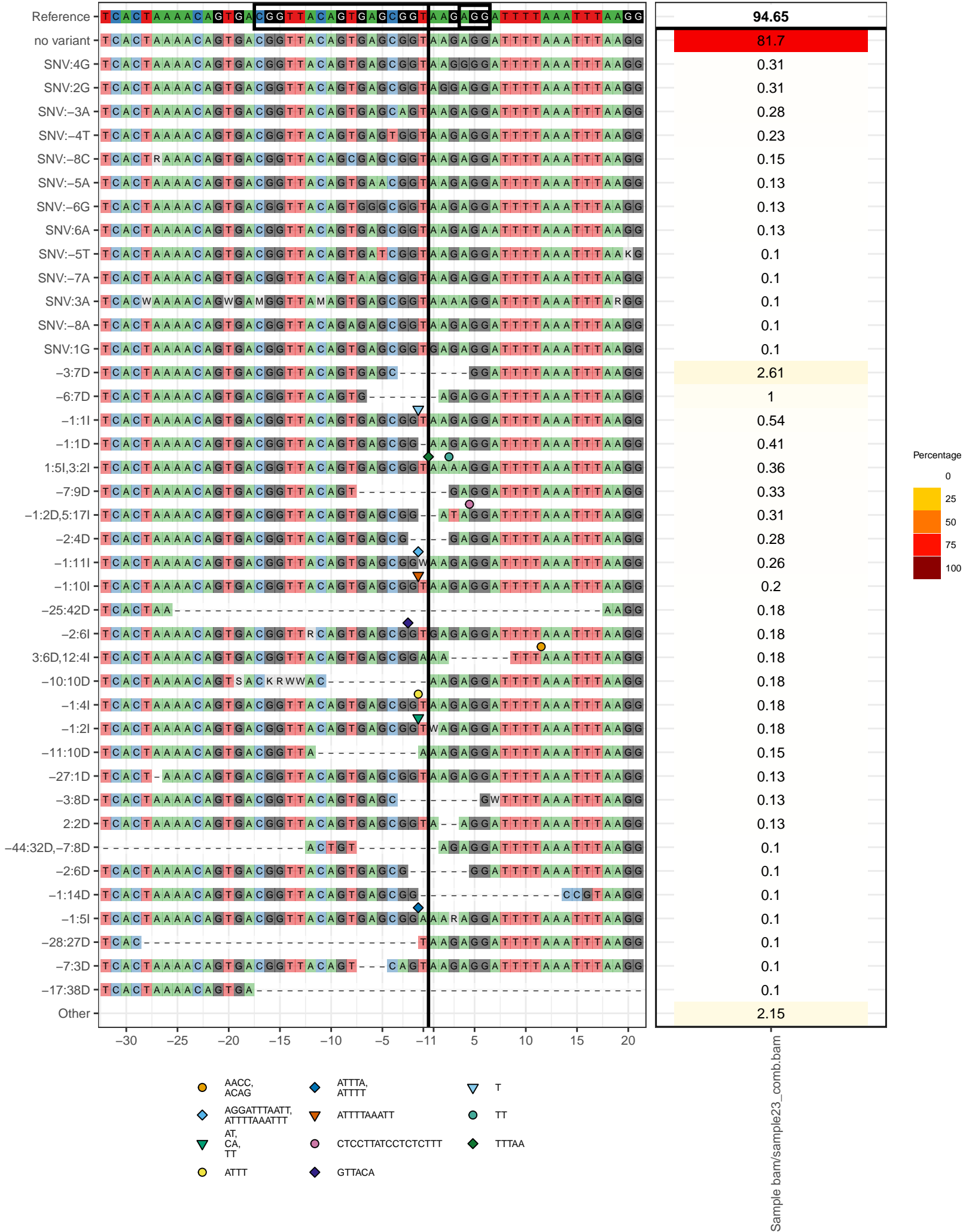

Supplement: Supplementary file 2 — Data S1. [file AUR-18-966-s002.zip › hepacam2.4.pdf]

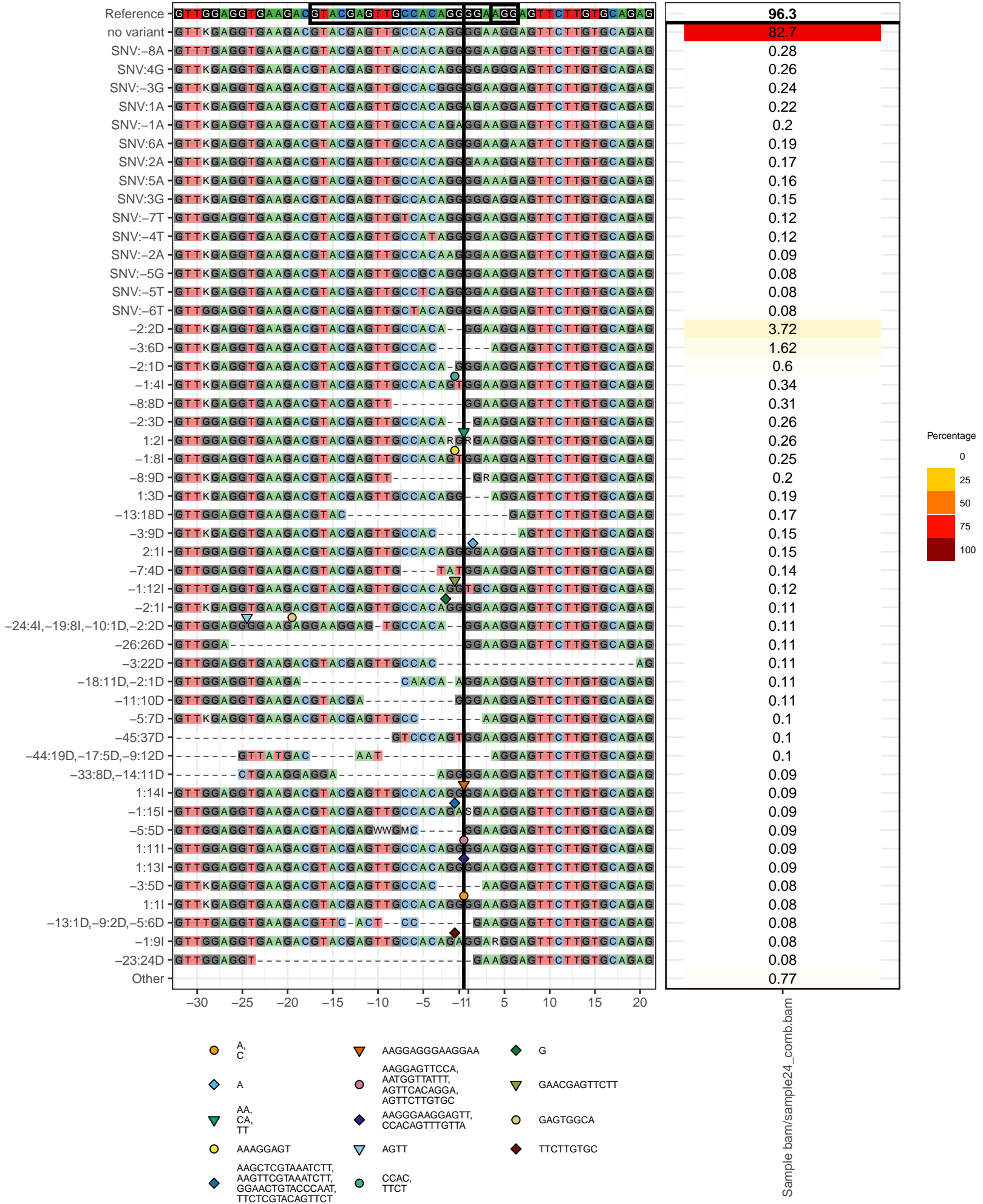

Supplement: Supplementary file 2 — Data S1. [file AUR-18-966-s002.zip › hepacam2.5.pdf]

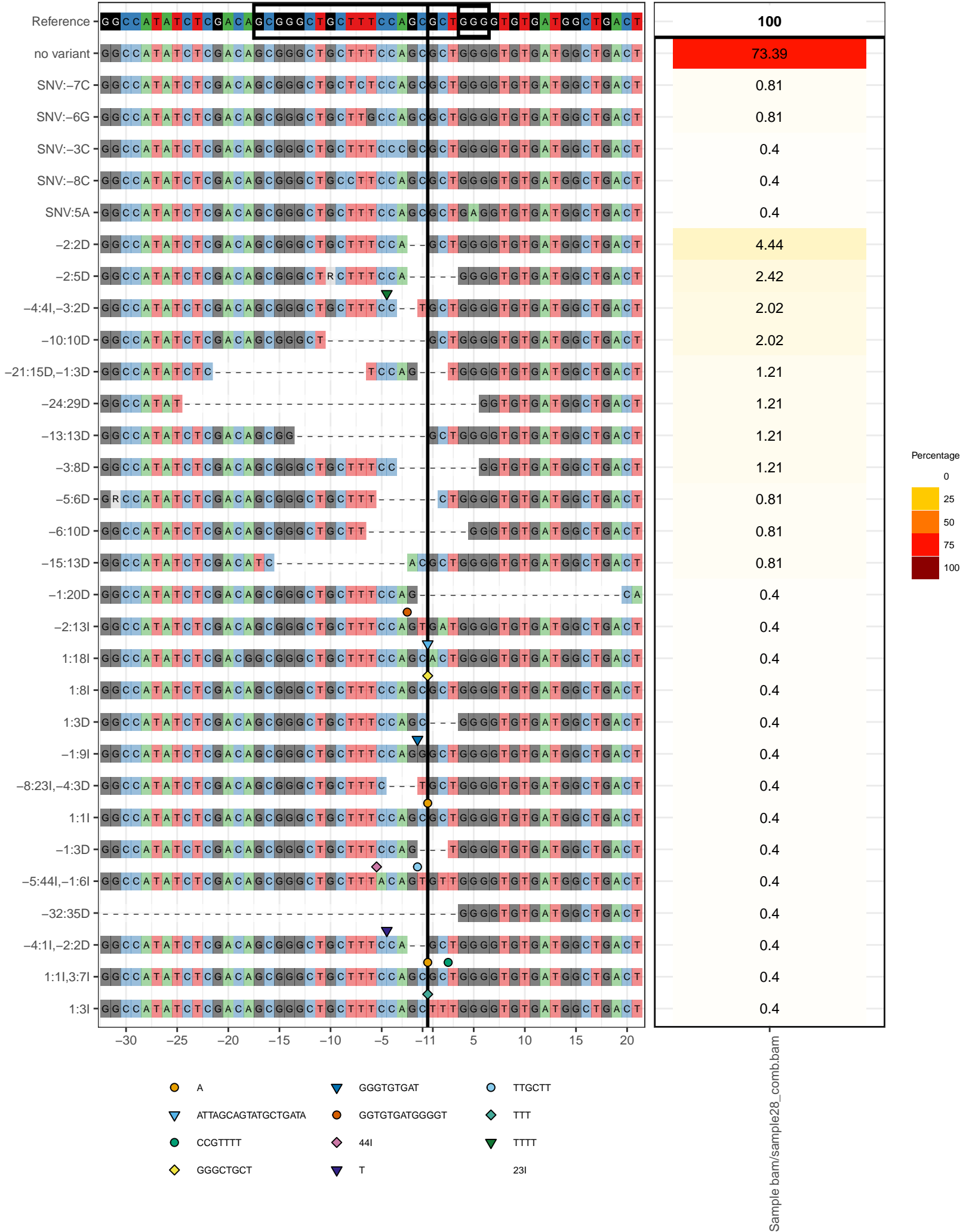

Supplement: Supplementary file 2 — Data S1. [file AUR-18-966-s002.zip › iars2.5.pdf]

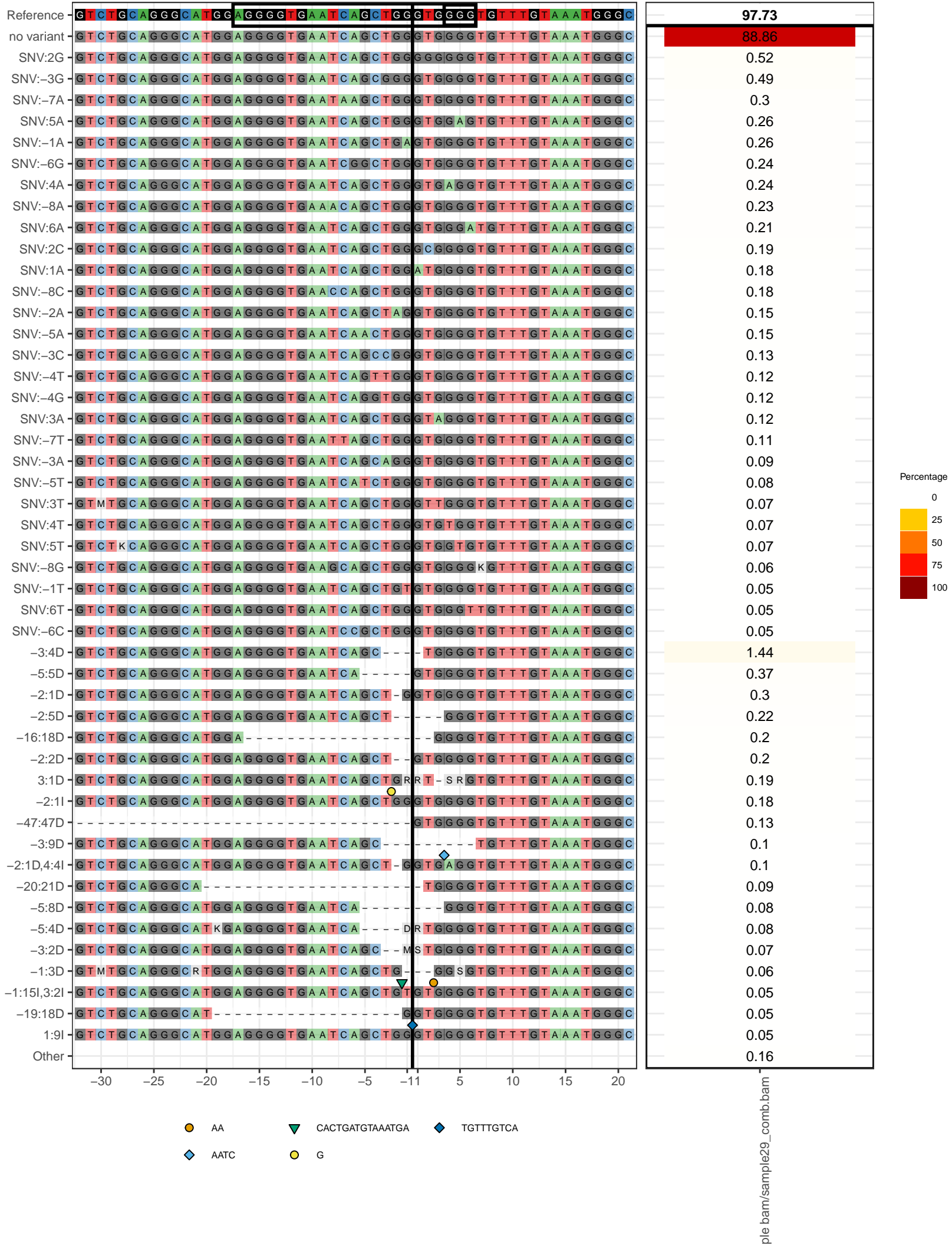

Supplement: Supplementary file 2 — Data S1. [file AUR-18-966-s002.zip › pax5.1.pdf]

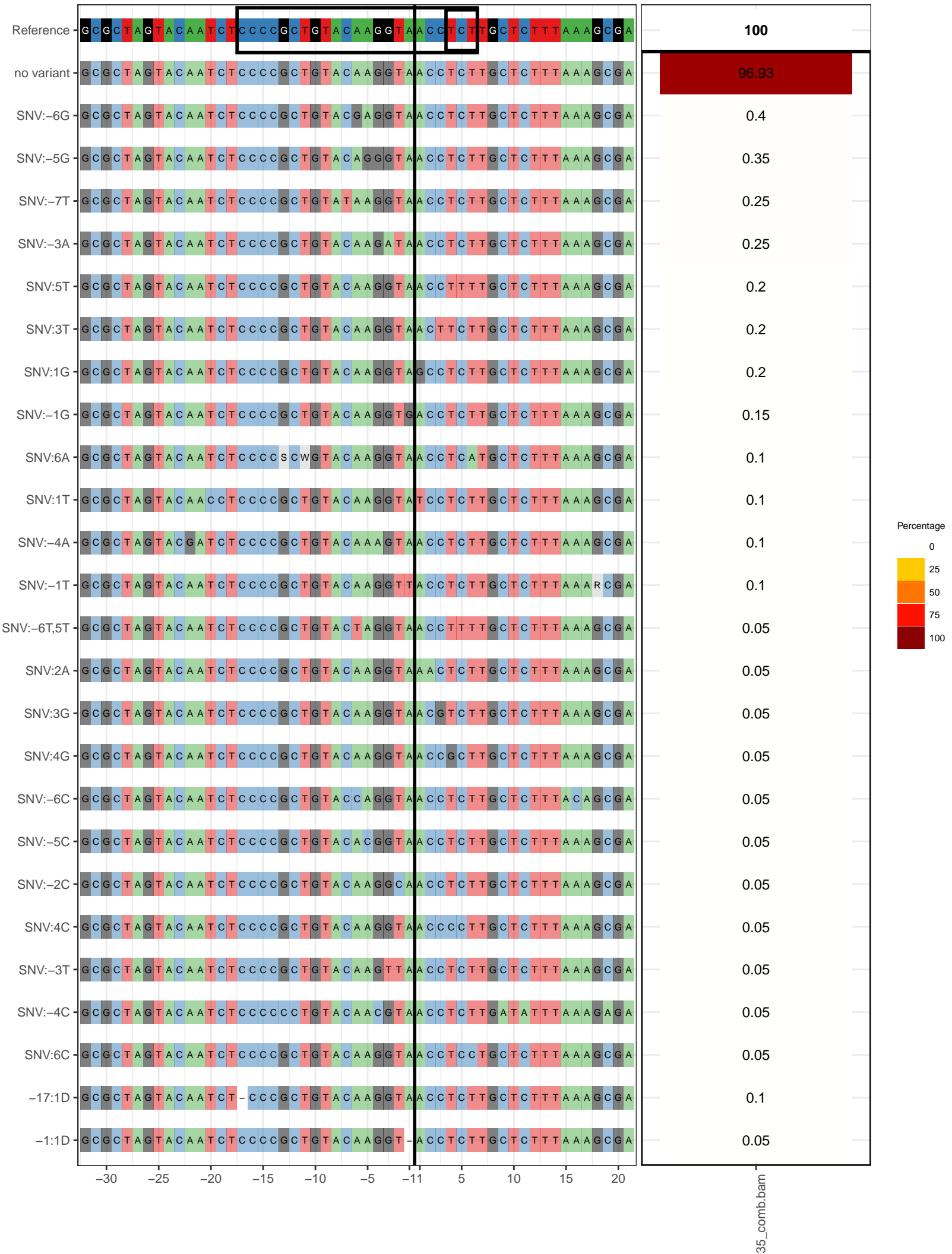

Supplement: Supplementary file 2 — Data S1. [file AUR-18-966-s002.zip › rps6ka.4.pdf]

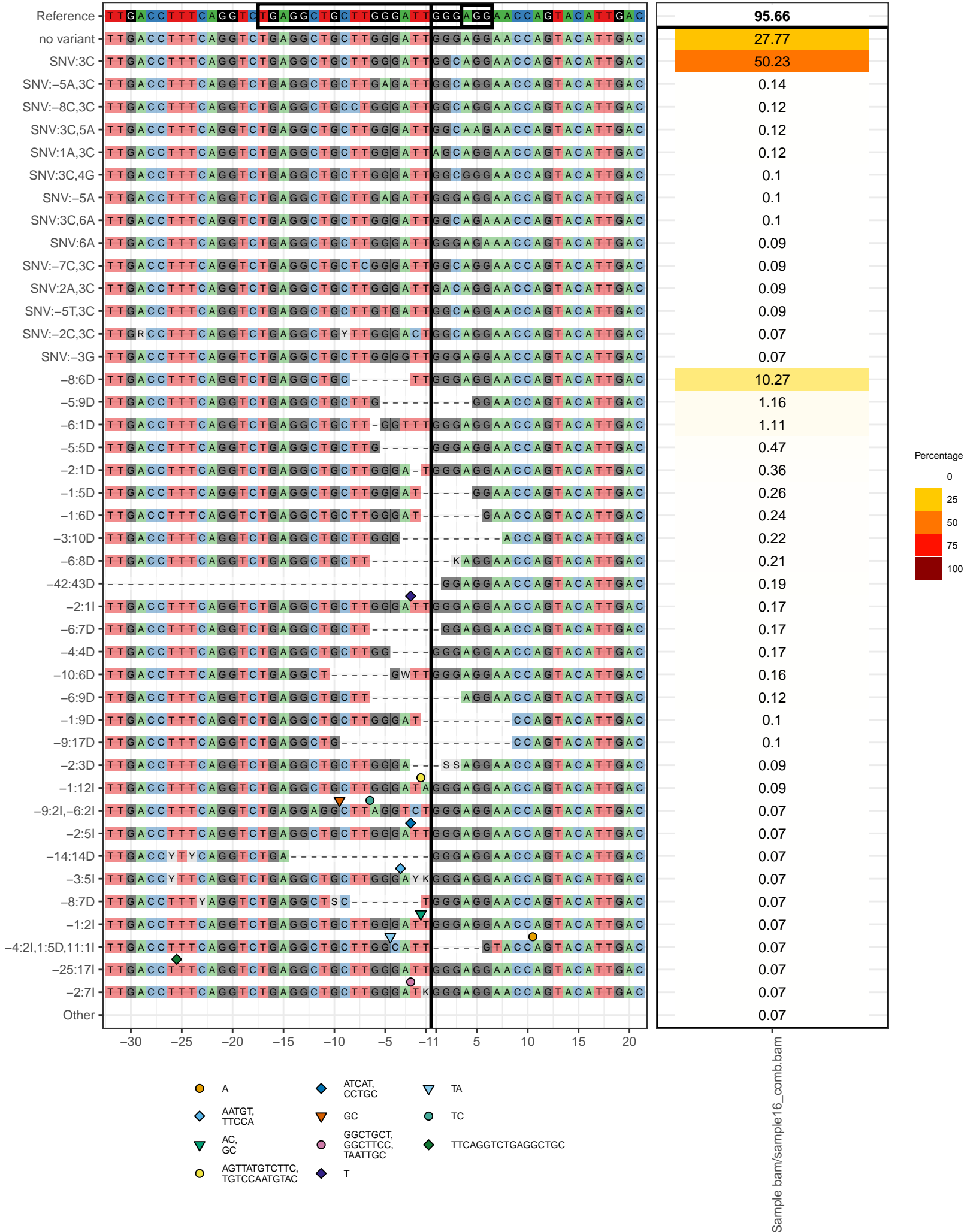

Supplement: Supplementary file 2 — Data S1. [file AUR-18-966-s002.zip › fam91a1.5.pdf]

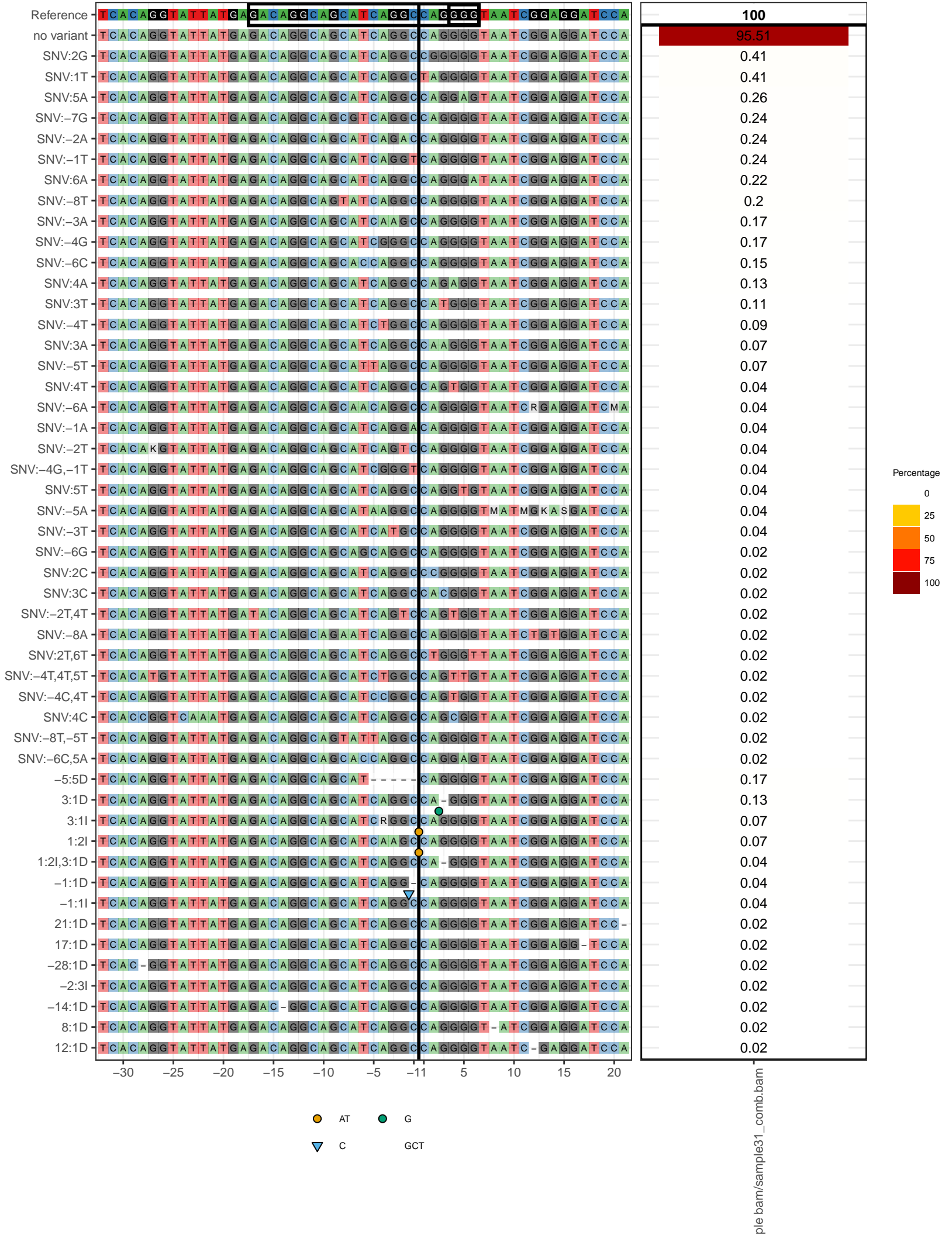

Supplement: Supplementary file 2 — Data S1. [file AUR-18-966-s002.zip › pax5.3.pdf]

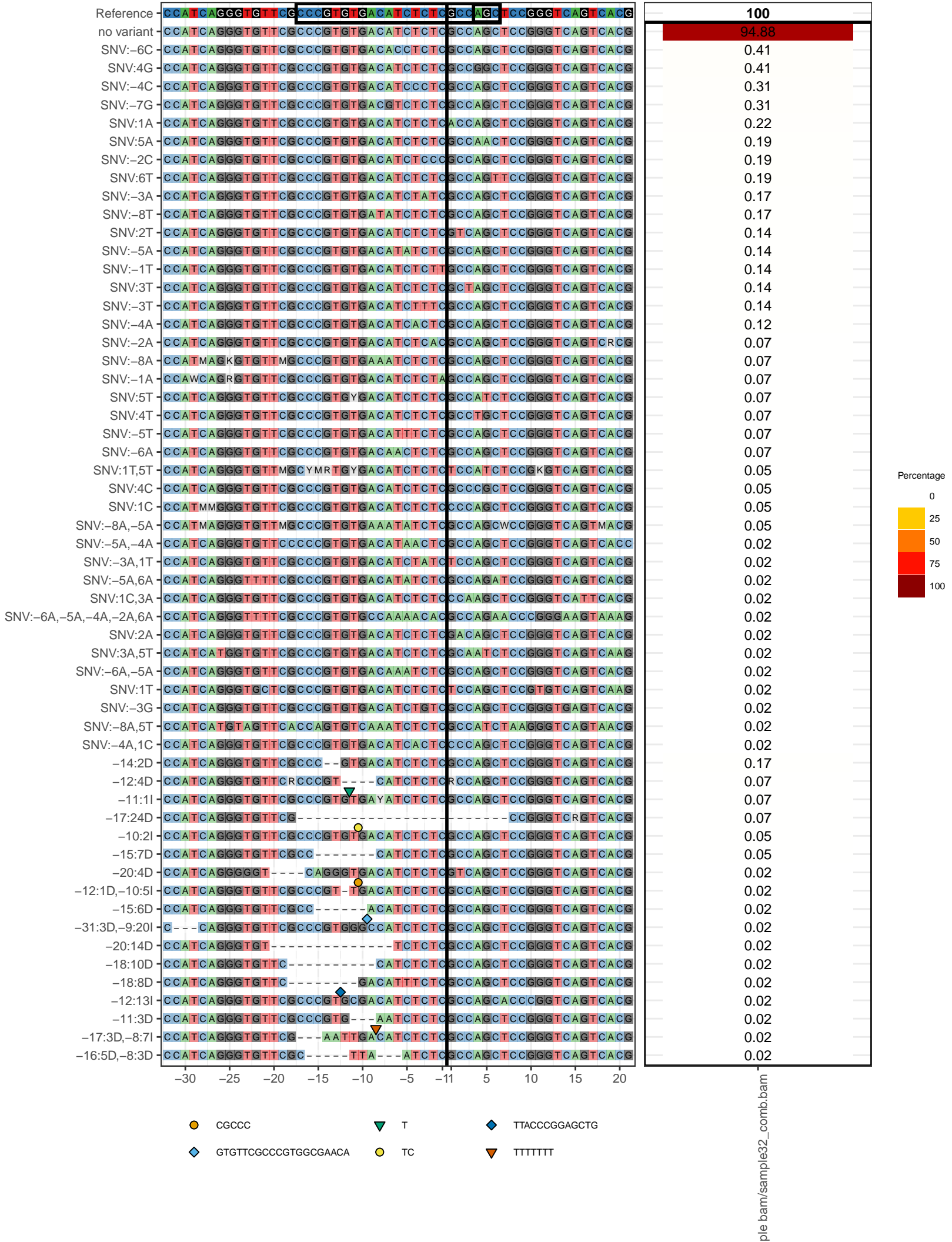

Supplement: Supplementary file 2 — Data S1. [file AUR-18-966-s002.zip › pax5.5.pdf]

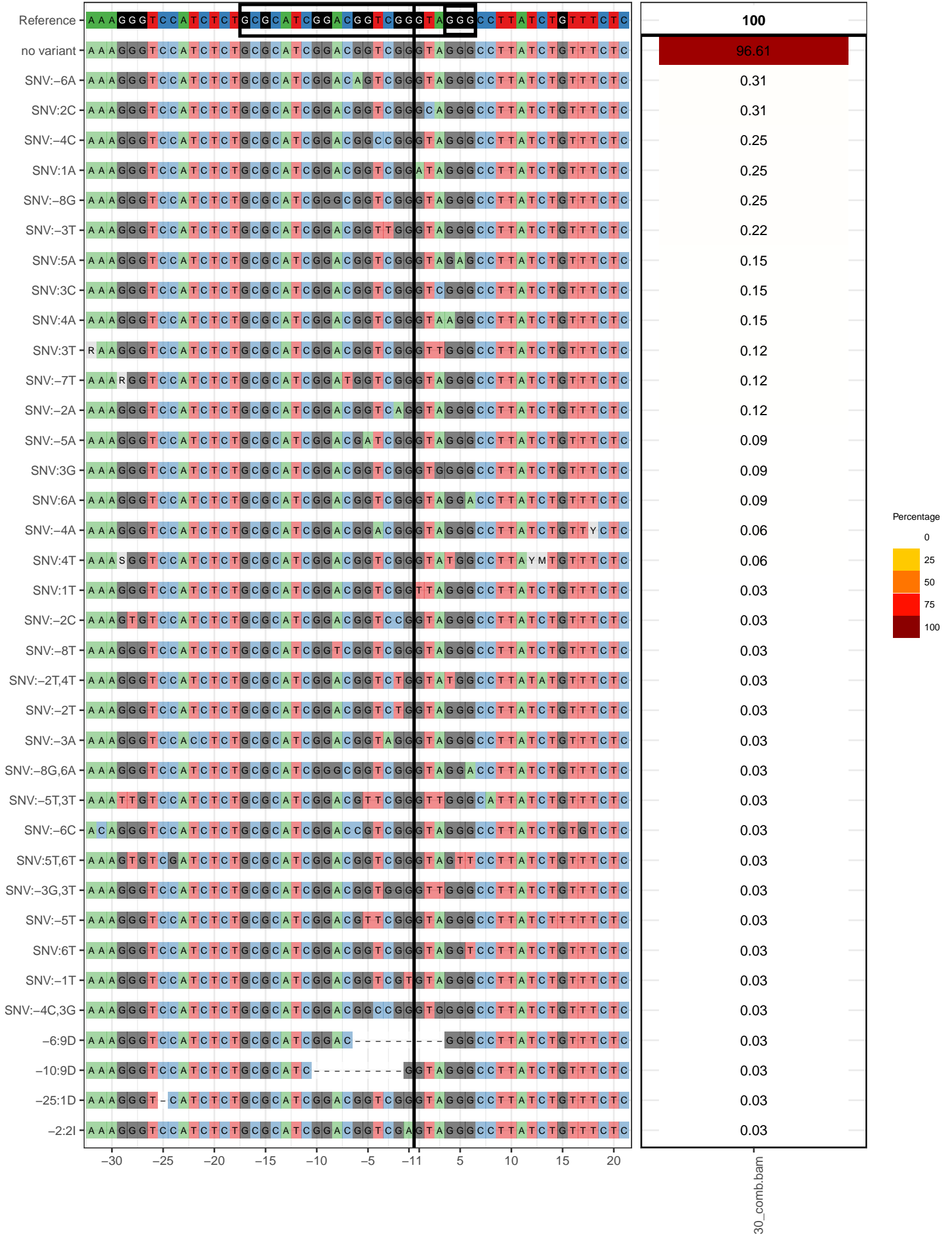

Supplement: Supplementary file 2 — Data S1. [file AUR-18-966-s002.zip › pax5.2.pdf]

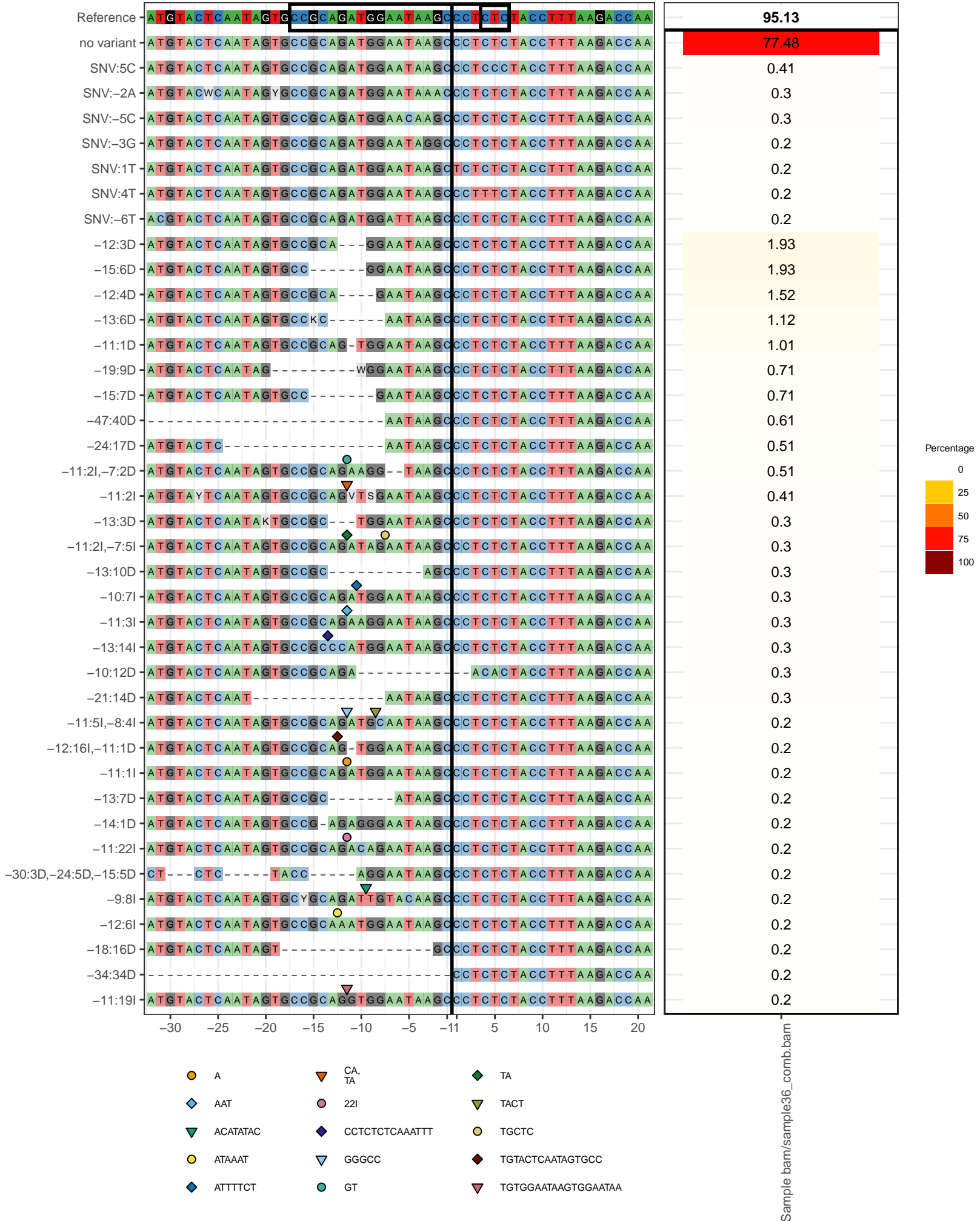

Supplement: Supplementary file 2 — Data S1. [file AUR-18-966-s002.zip › rps6ka.5.pdf]

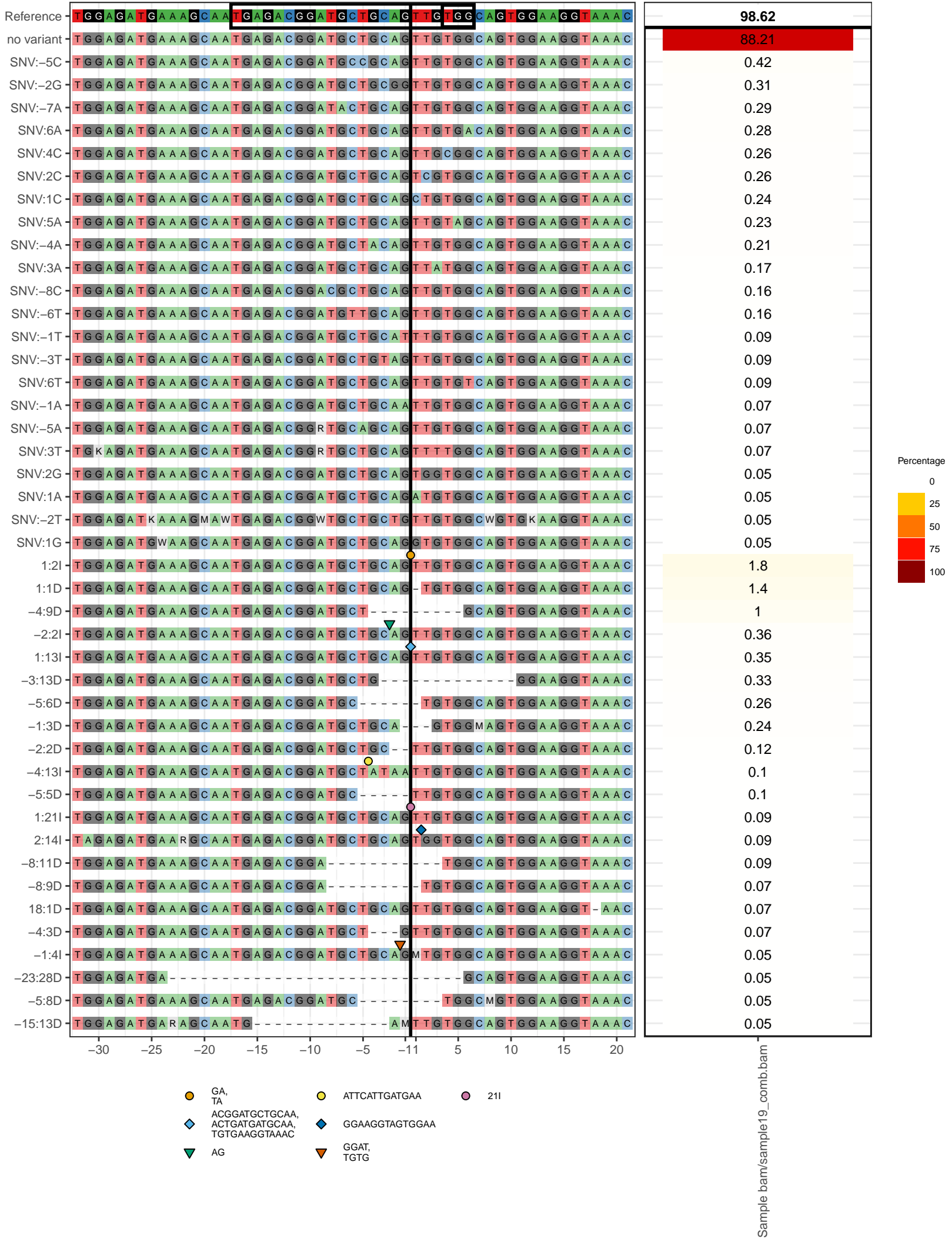

Supplement: Supplementary file 2 — Data S1. [file AUR-18-966-s002.zip › gmeb1.4.pdf]

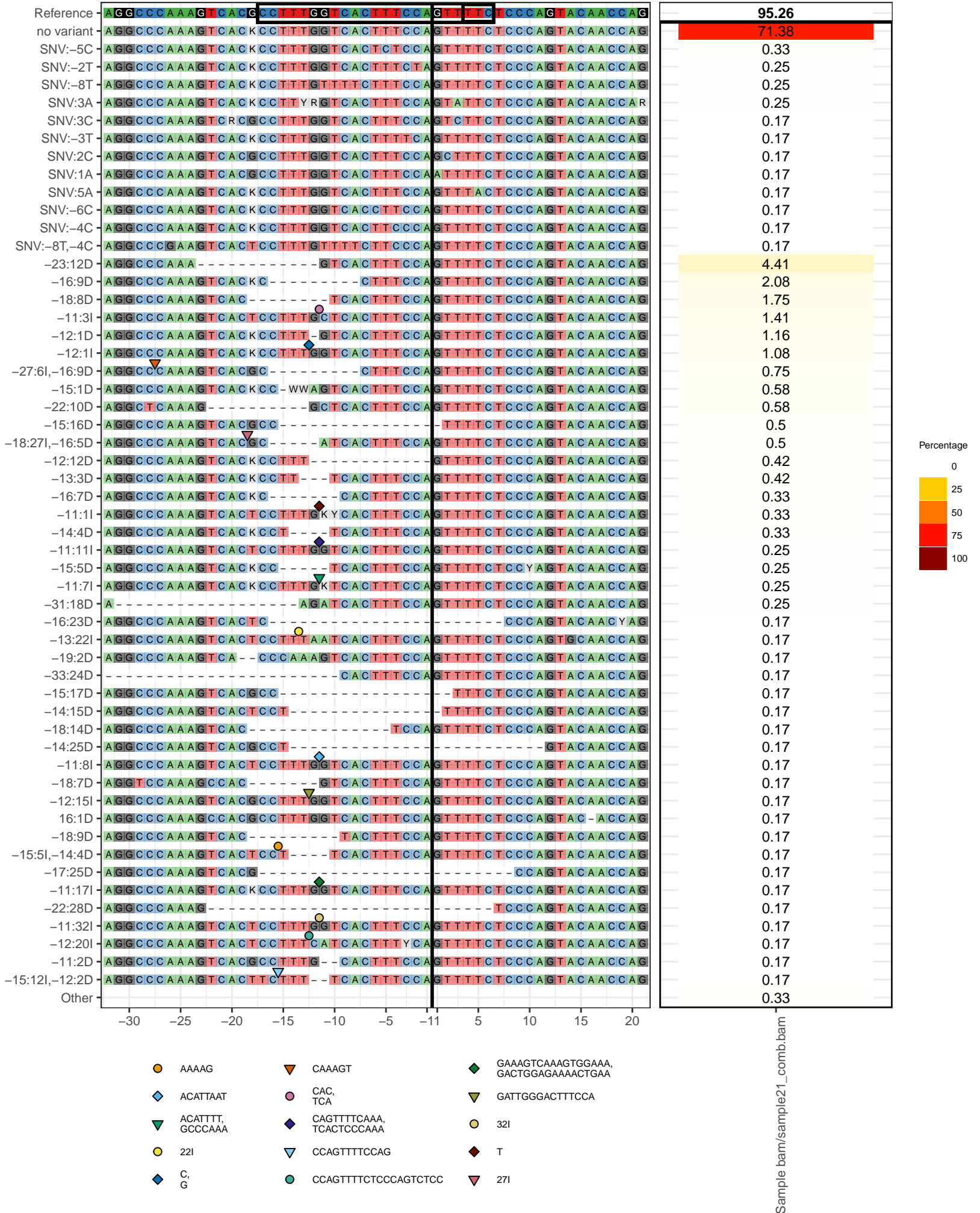

Supplement: Supplementary file 2 — Data S1. [file AUR-18-966-s002.zip › hepacam2.1.pdf]

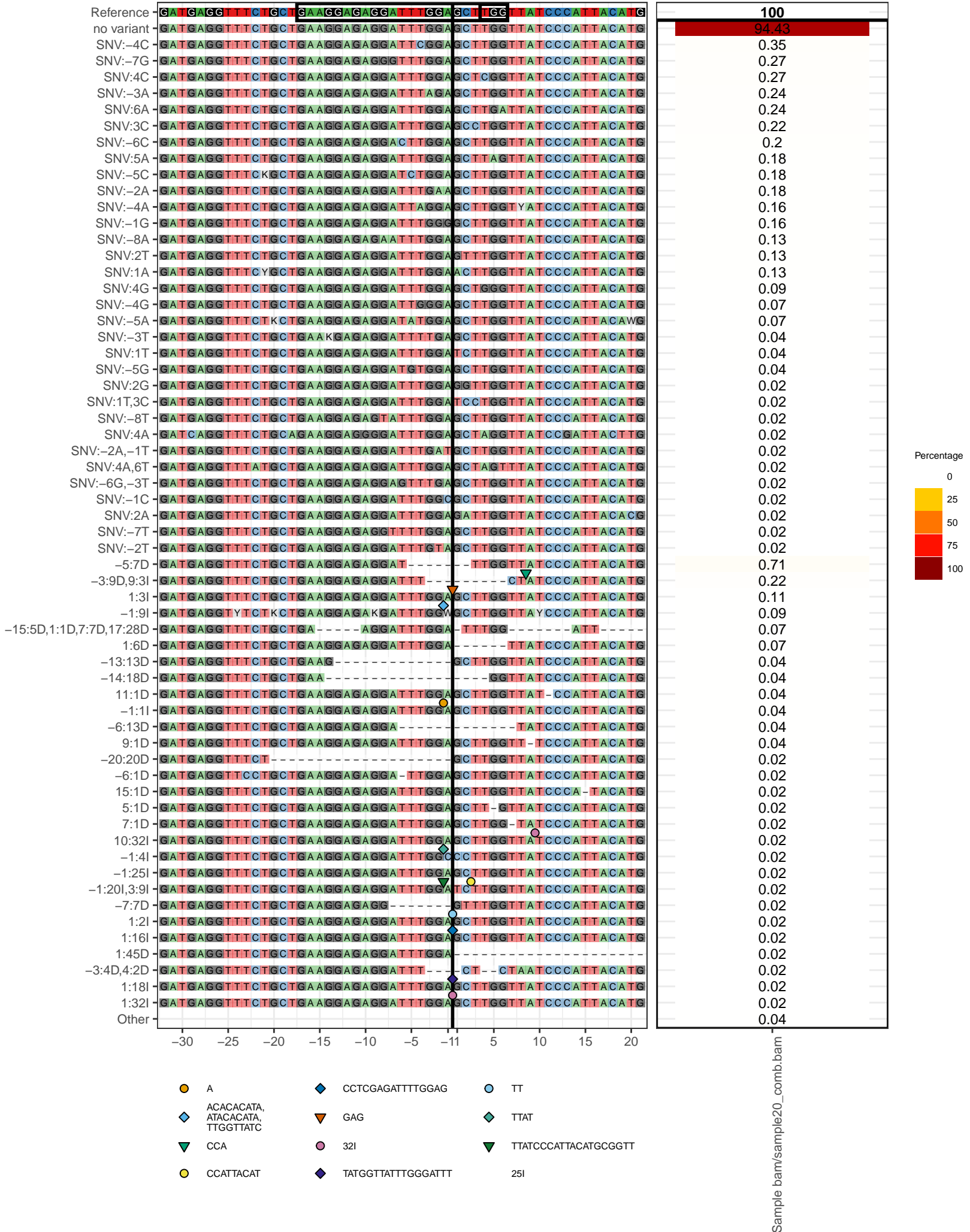

Supplement: Supplementary file 2 — Data S1. [file AUR-18-966-s002.zip › gmeb1.5.pdf]

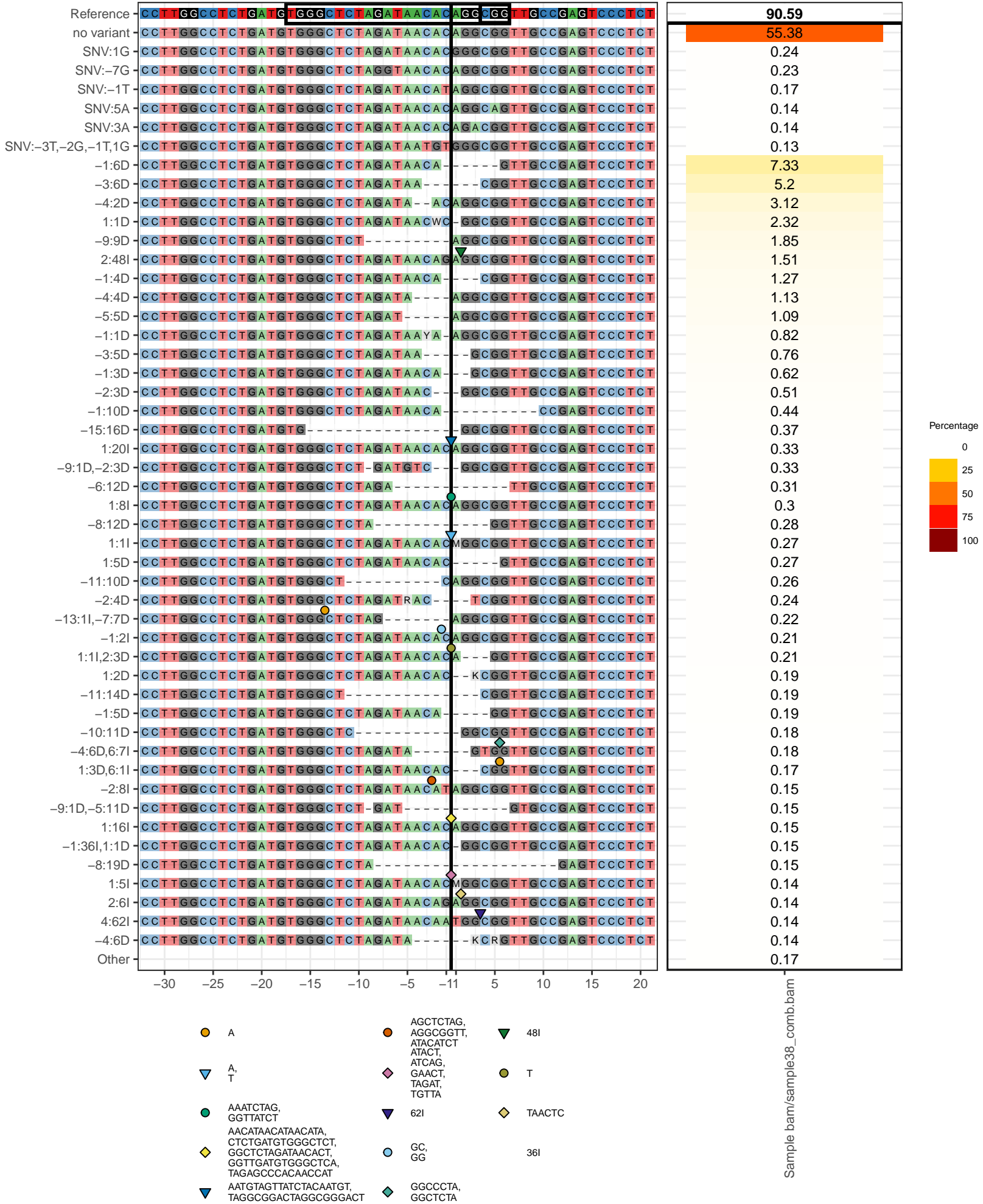

Supplement: Supplementary file 2 — Data S1. [file AUR-18-966-s002.zip › ryr3.2.pdf]

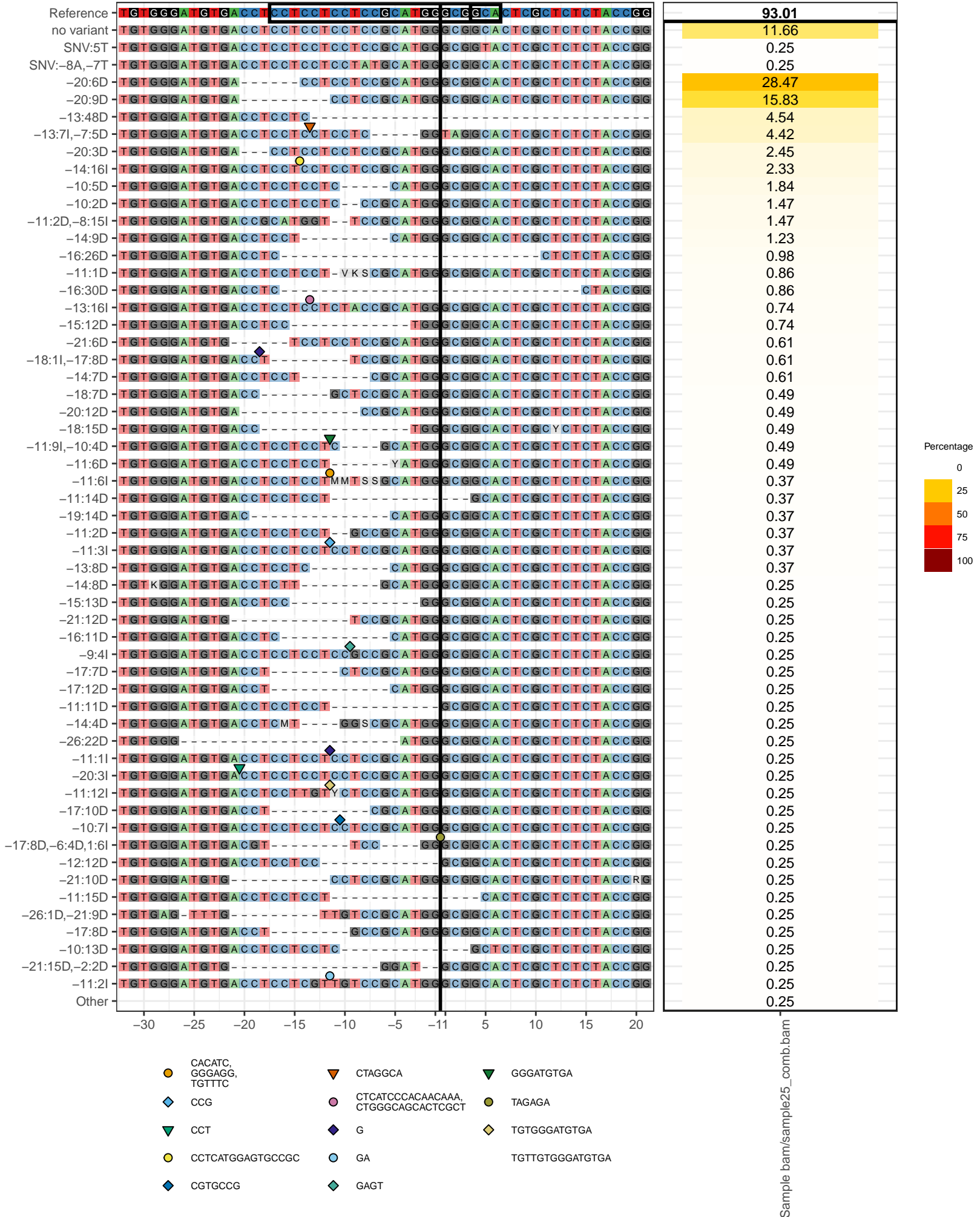

Supplement: Supplementary file 2 — Data S1. [file AUR-18-966-s002.zip › iars2.1.pdf]

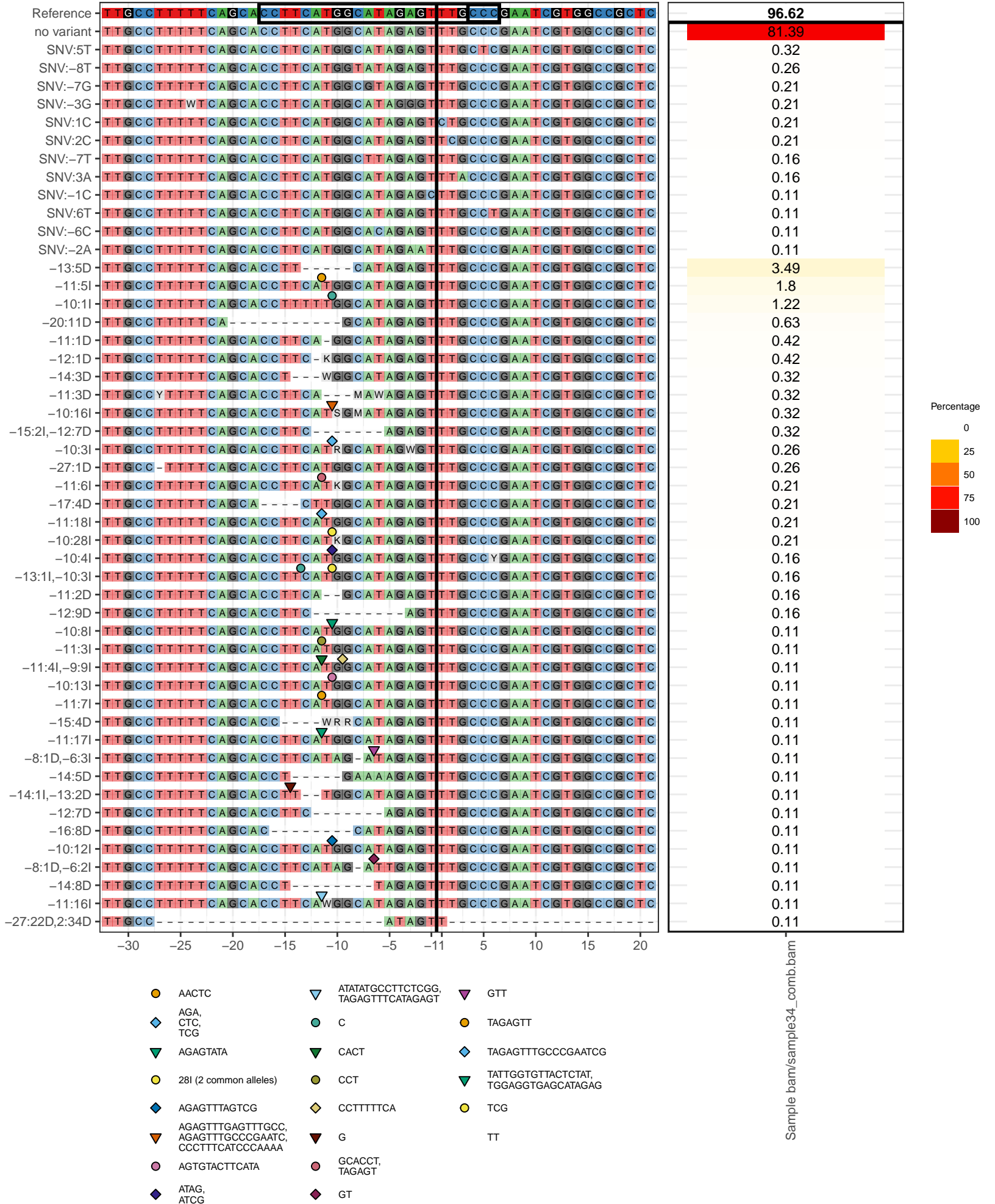

Supplement: Supplementary file 2 — Data S1. [file AUR-18-966-s002.zip › rps6ka.2.pdf]

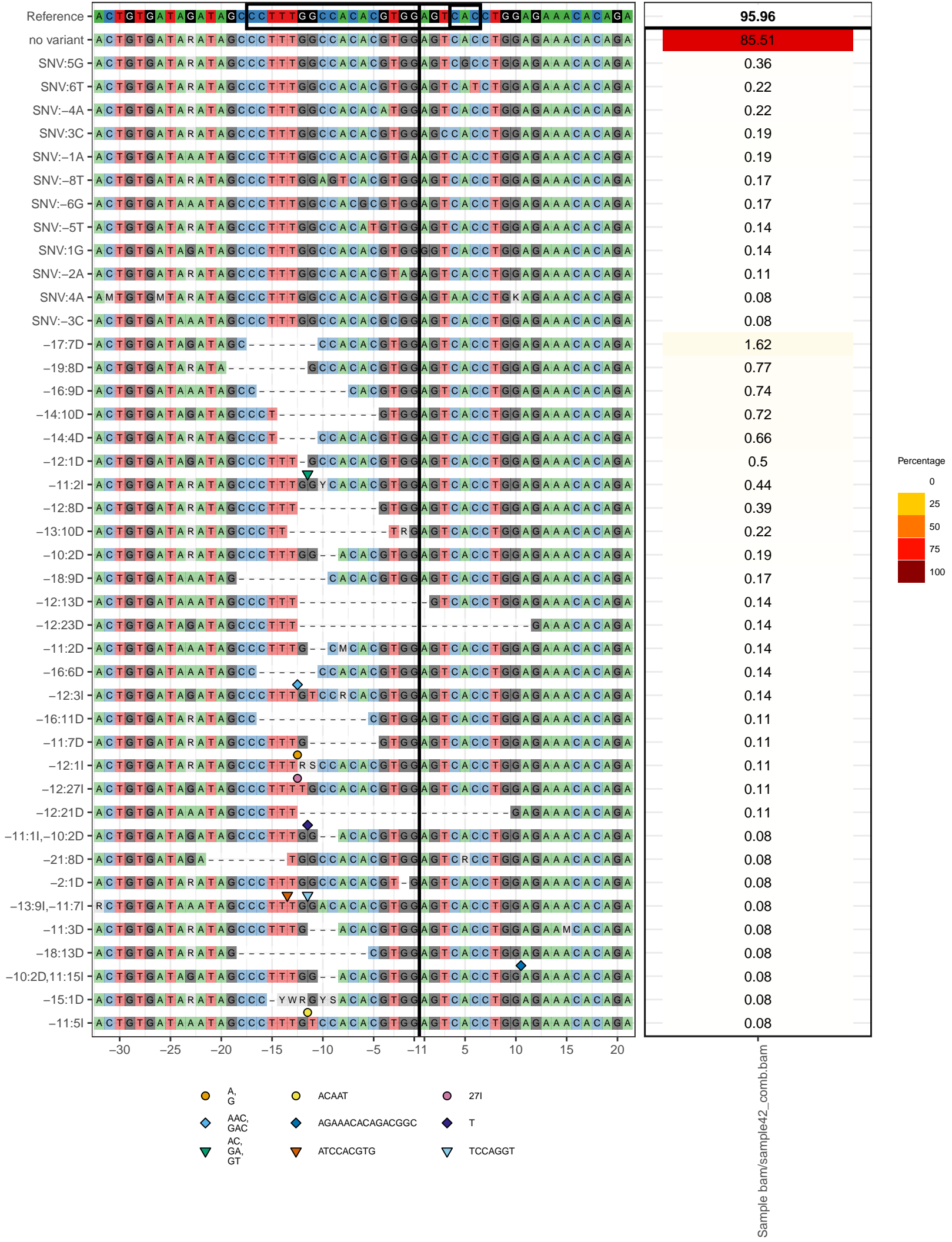

Supplement: Supplementary file 2 — Data S1. [file AUR-18-966-s002.zip › scp2a.2.pdf]

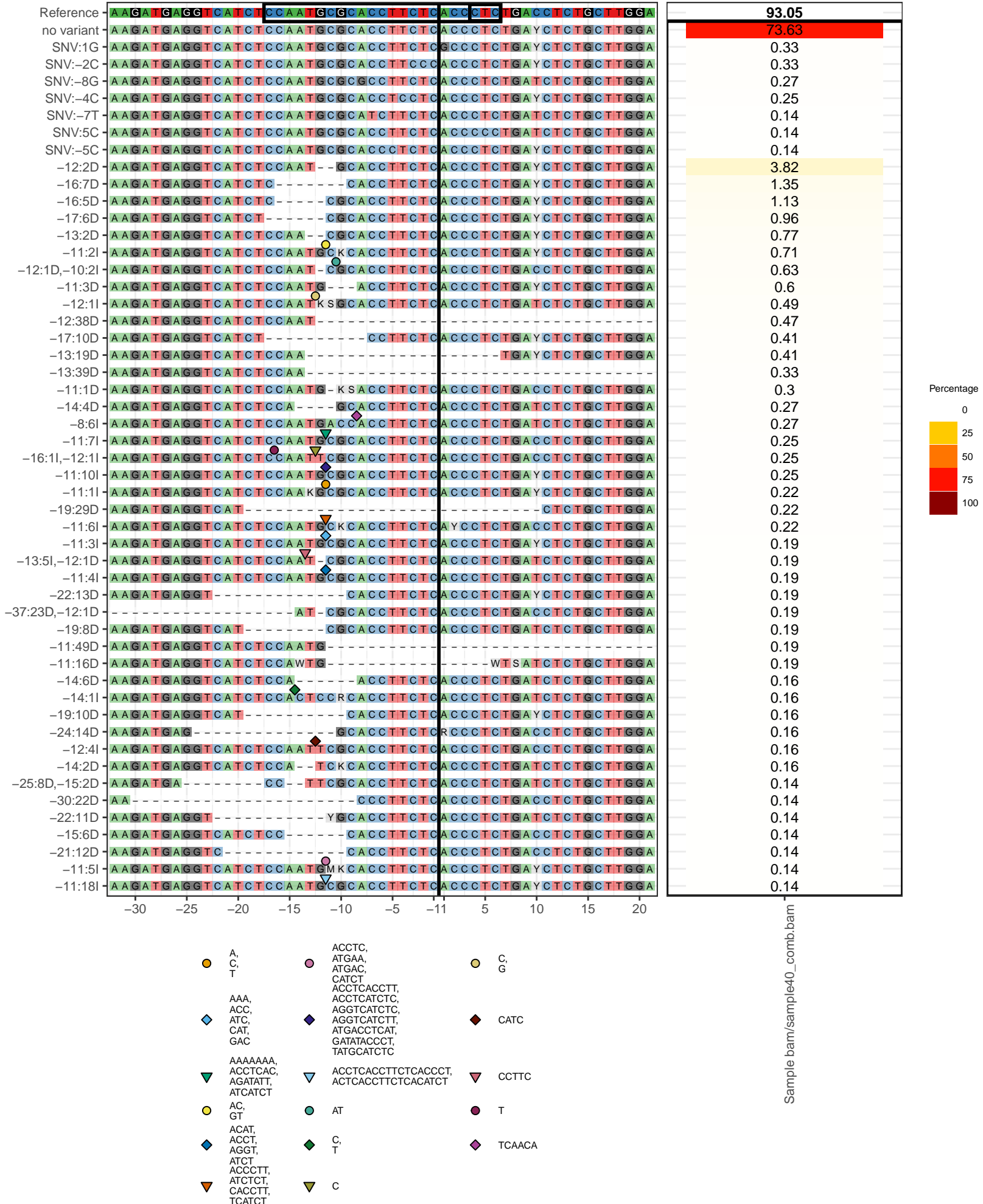

Supplement: Supplementary file 2 — Data S1. [file AUR-18-966-s002.zip › ryr3.5.pdf]

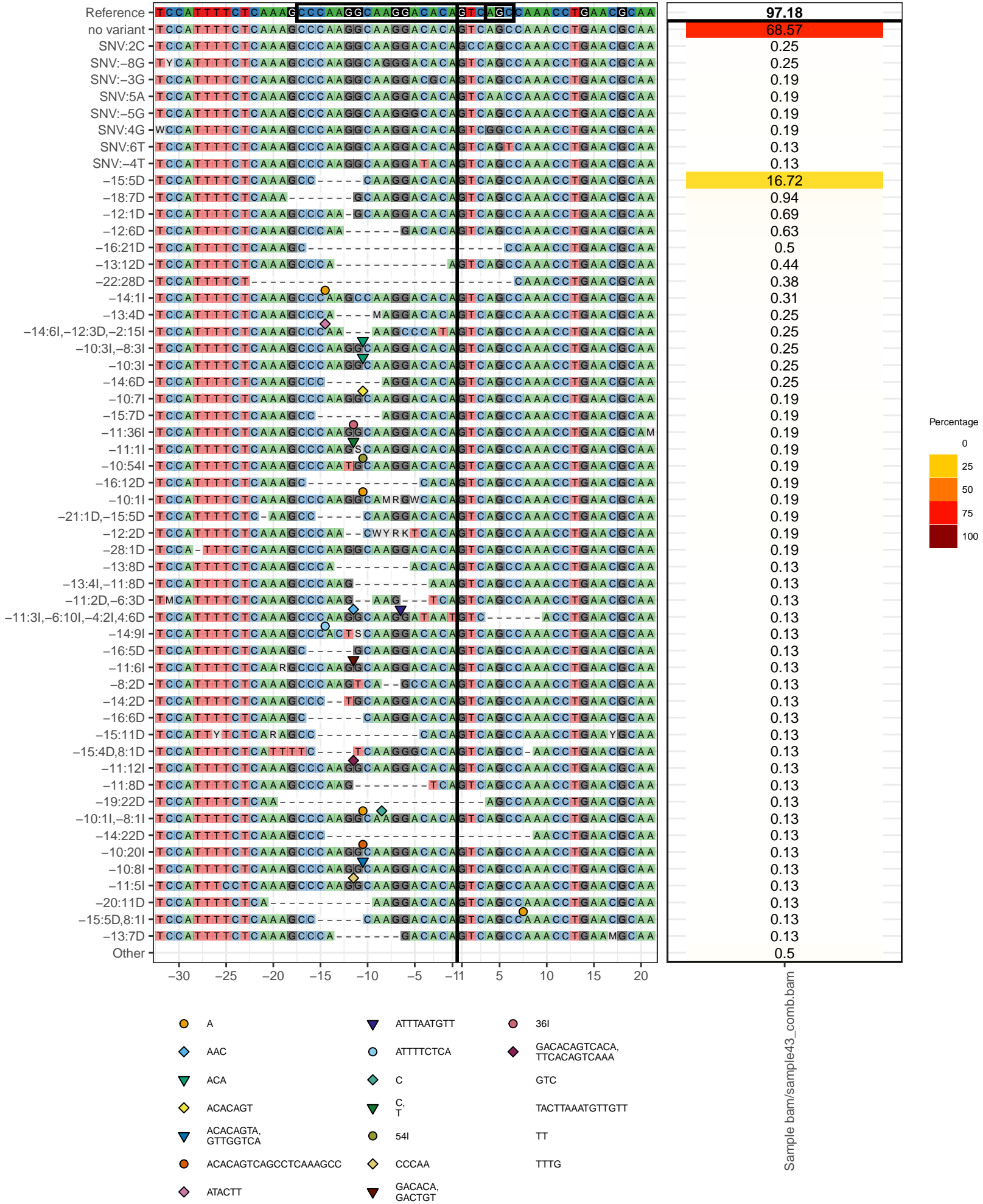

Supplement: Supplementary file 2 — Data S1. [file AUR-18-966-s002.zip › scp2a.4.pdf]

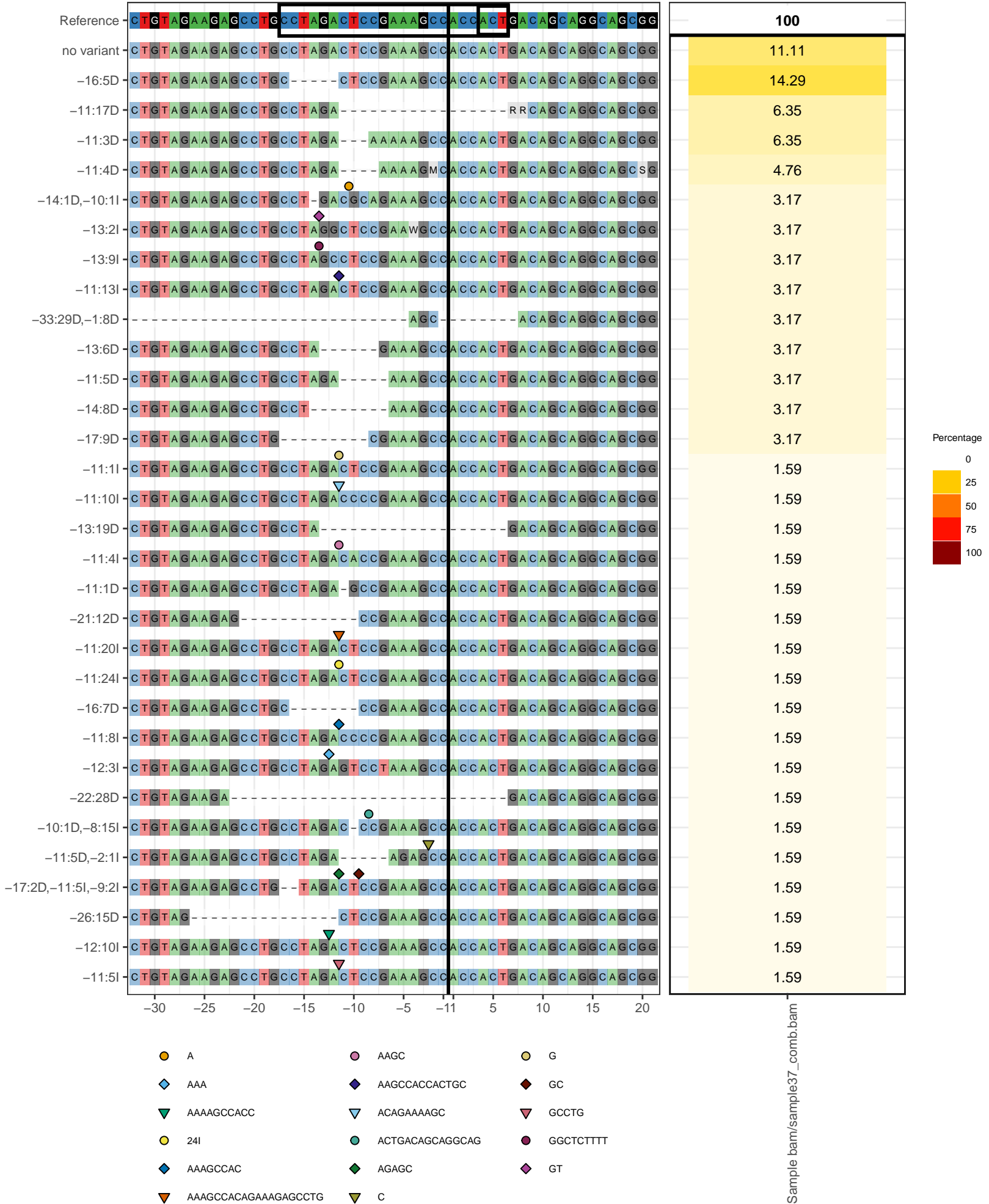

Supplement: Supplementary file 2 — Data S1. [file AUR-18-966-s002.zip › ryr3.1.pdf]

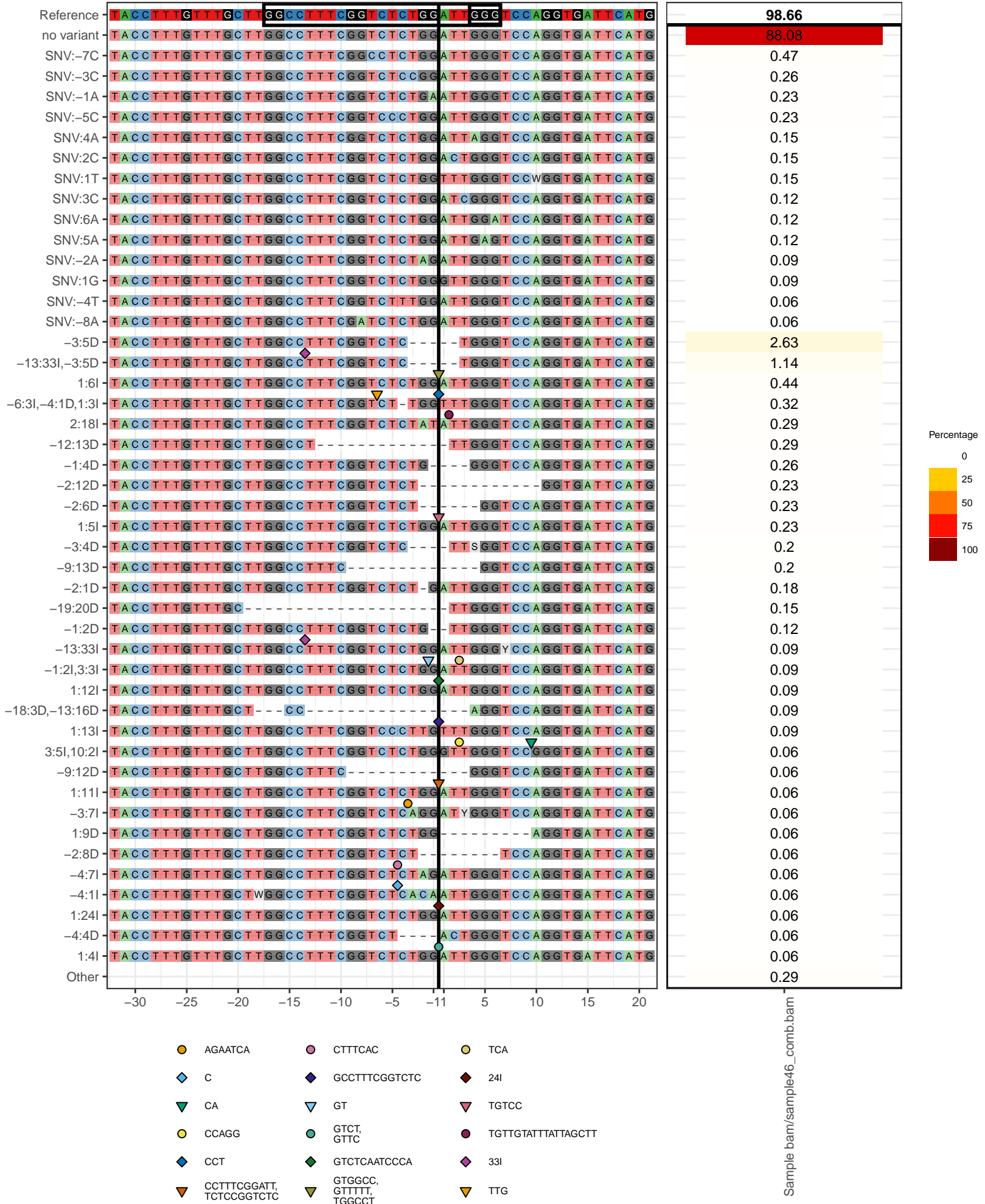

Supplement: Supplementary file 2 — Data S1. [file AUR-18-966-s002.zip › ythdf2.2.pdf]

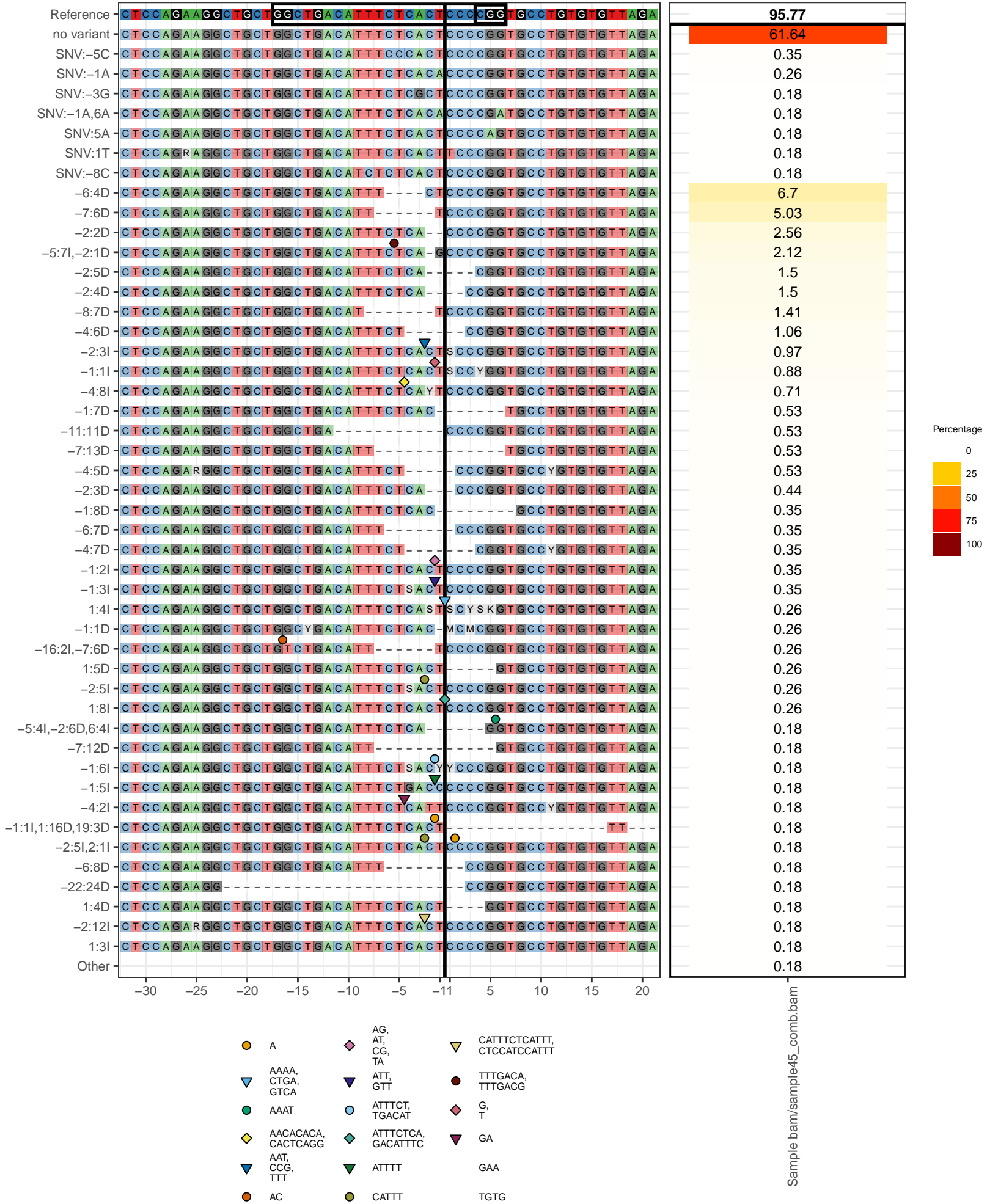

Supplement: Supplementary file 2 — Data S1. [file AUR-18-966-s002.zip › ythdf2.1.pdf]

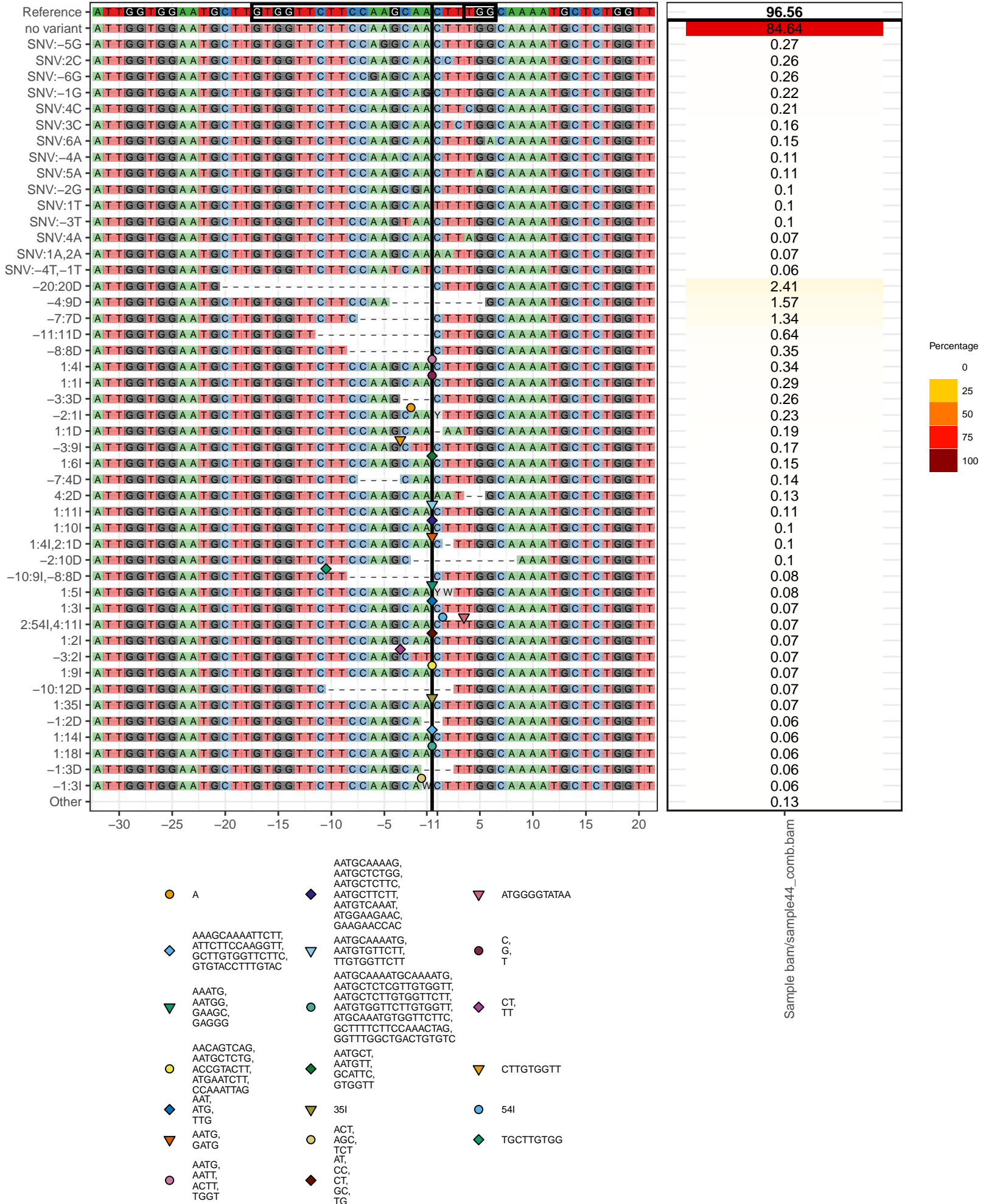

Supplement: Supplementary file 2 — Data S1. [file AUR-18-966-s002.zip › scp2a.5.pdf]

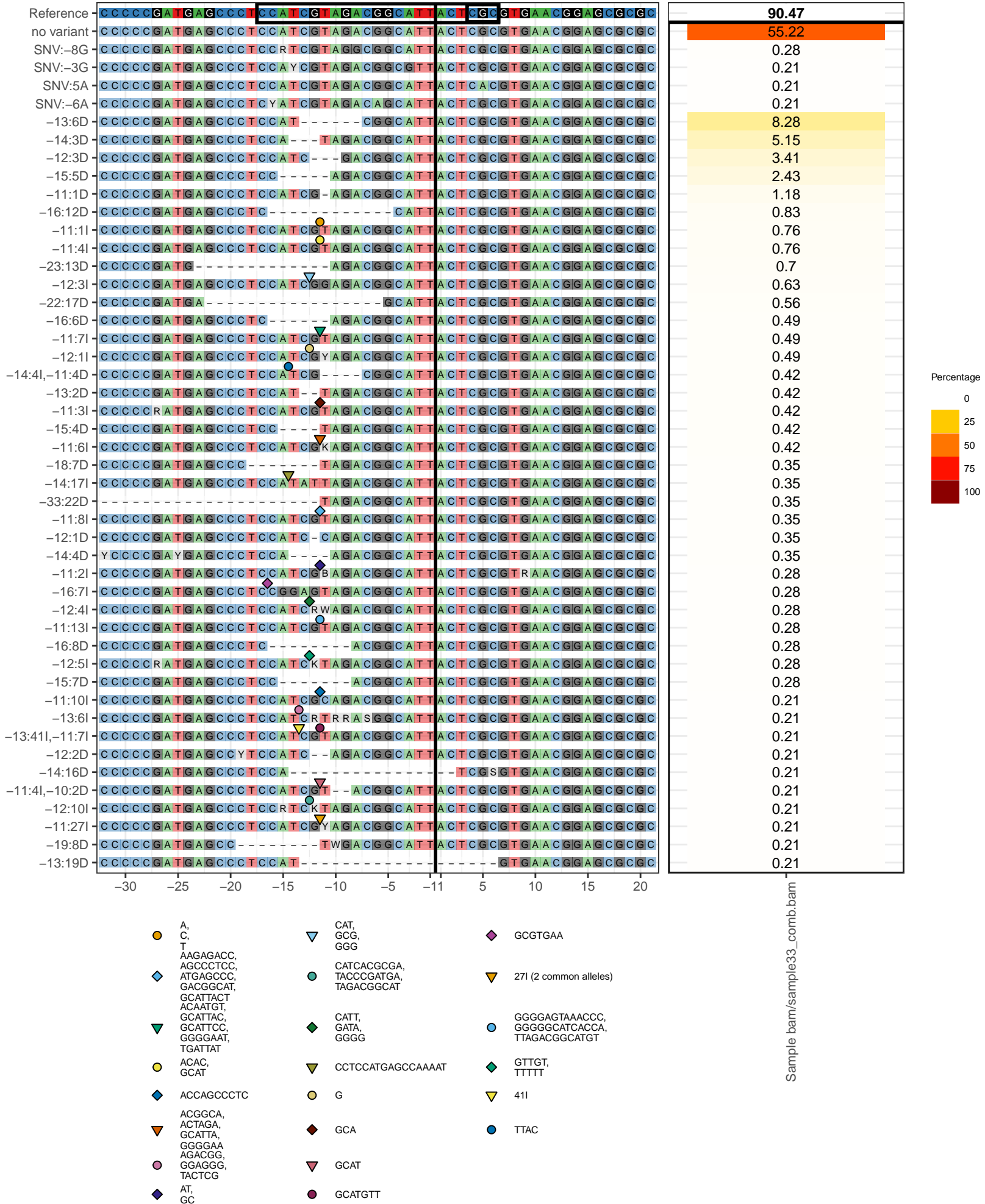

Supplement: Supplementary file 2 — Data S1. [file AUR-18-966-s002.zip › rps6ka.1.pdf]

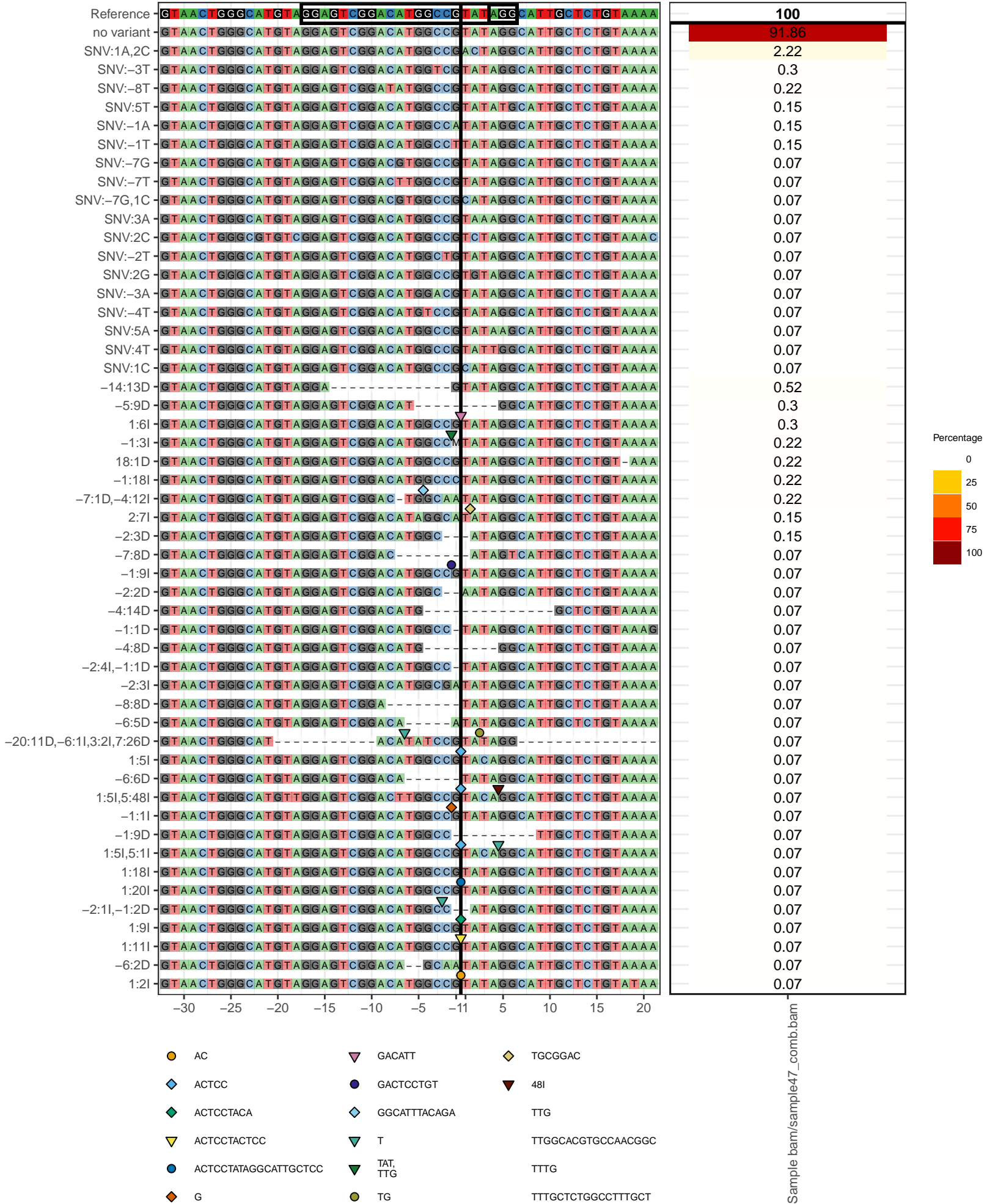

Supplement: Supplementary file 2 — Data S1. [file AUR-18-966-s002.zip › ythdf2.4.pdf]

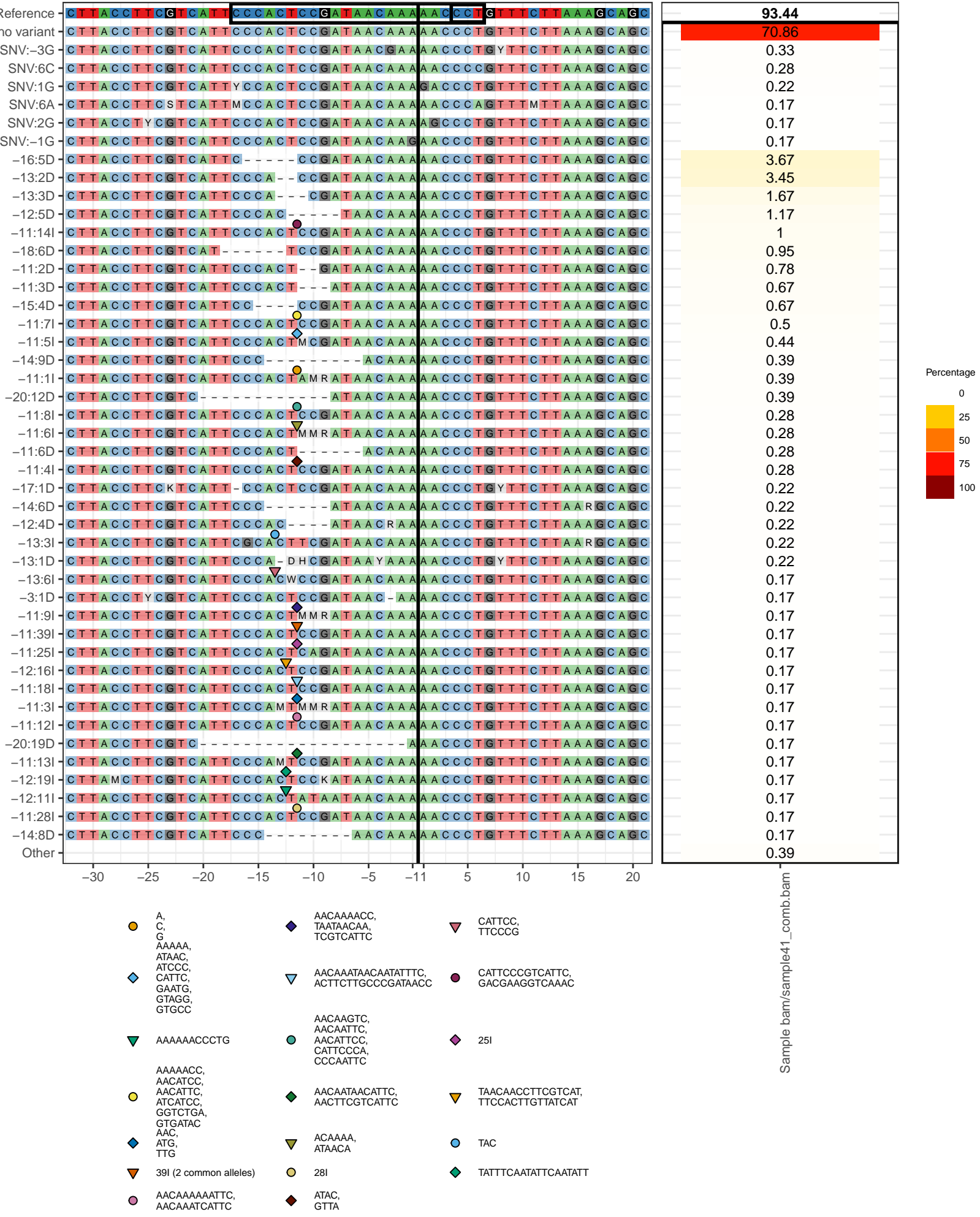

Supplement: Supplementary file 2 — Data S1. [file AUR-18-966-s002.zip › scp2a.1.pdf]

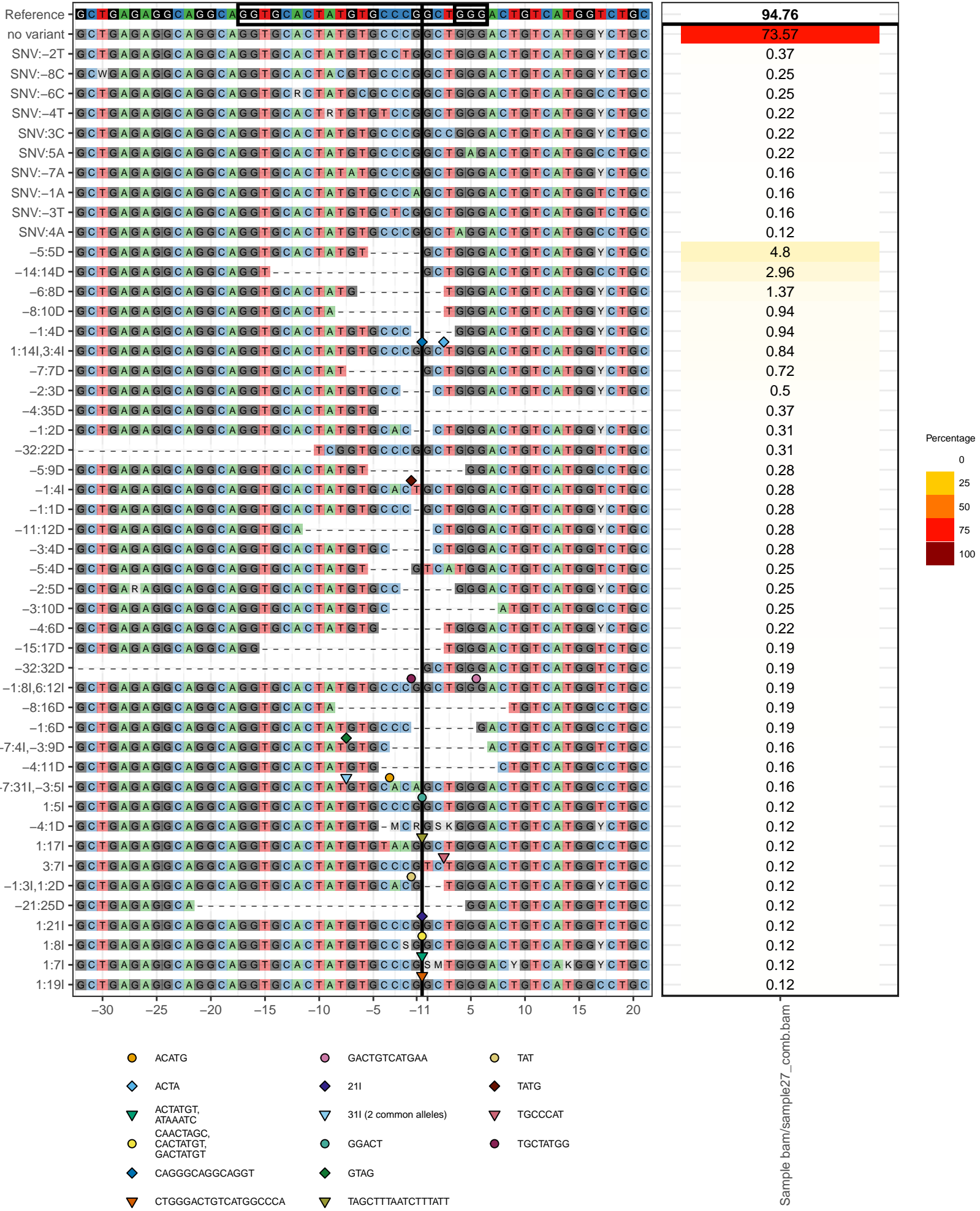

Supplement: Supplementary file 2 — Data S1. [file AUR-18-966-s002.zip › iars2.4.pdf]

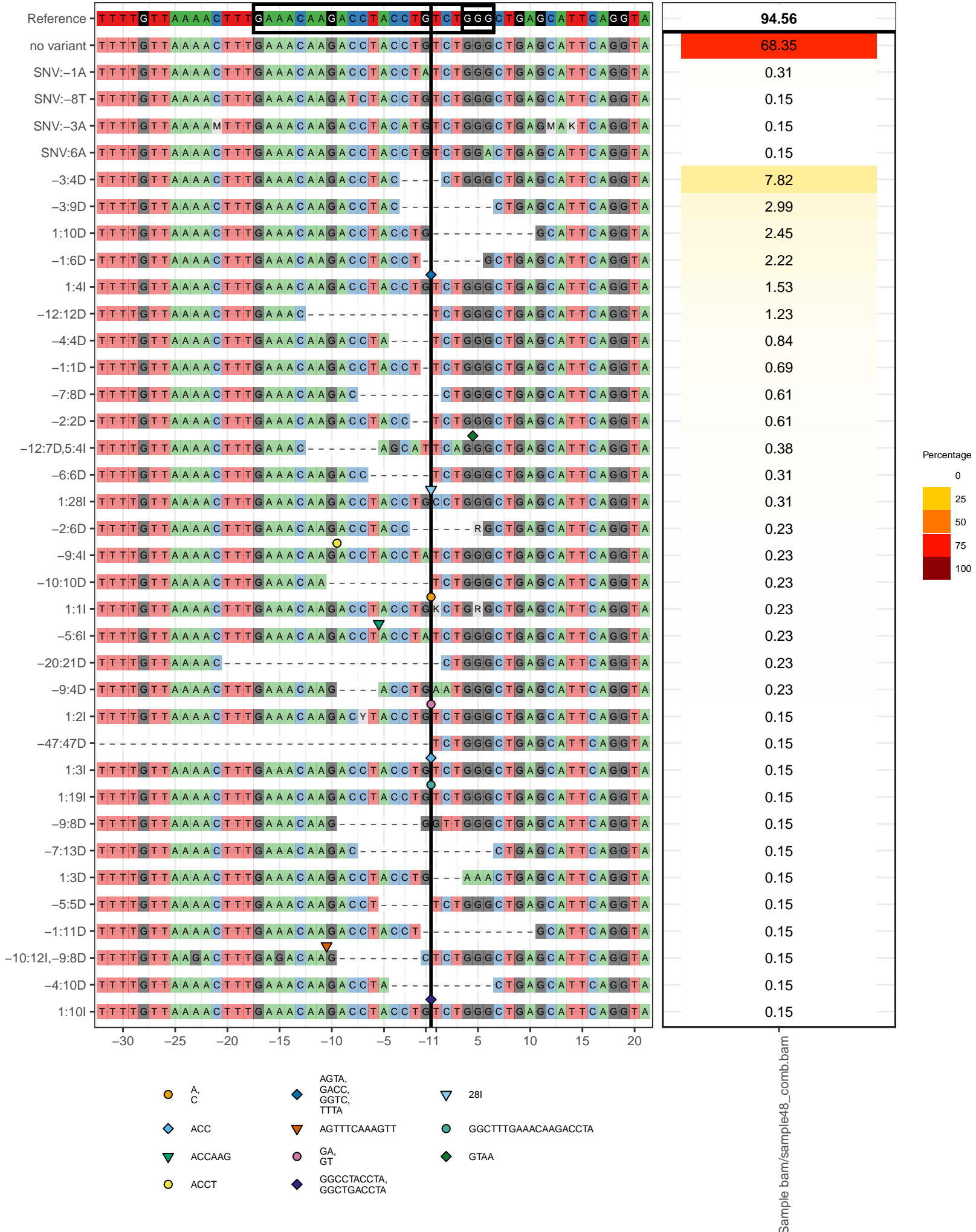

Supplement: Supplementary file 2 — Data S1. [file AUR-18-966-s002.zip › ythdf2.5.pdf]
